# Revisiting Hafner’s Azapentalenes: The Chemistry of 1,3-Bis(dimethylamino)-2-azapentalene

**DOI:** 10.1021/acs.joc.3c02564

**Published:** 2024-04-17

**Authors:** Enikő Meiszter, Tamás Gazdag, Péter J. Mayer, Attila Kunfi, Tamás Holczbauer, Máté Sulyok-Eiler, Gábor London

**Affiliations:** † MTA TTK Lendület Functional Organic Materials Research Group, Institute of Organic Chemistry, HUN-REN Research Centre for Natural Sciences, Magyar tudósok krt. 2, 1117 Budapest, Hungary; ‡ Department of Organic Chemistry and Technology, Faculty of Chemical Technology and Biotechnology, Budapest University of Technology and Economics, Műegyetem rkp. 3, H-1111 Budapest, Hungary; § Hevesy György PhD School of Chemistry, Eötvös Loránd University, Pázmány Péter Sétány 1/a, 1117 Budapest, Hungary; ∥ Chemical Crystallography Research Laboratory and Stereochemistry Research Group, Institute for Organic Chemistry, HUN-REN Research Centre for Natural Sciences, Magyar tudósok krt. 2, 1117 Budapest, Hungary; ⊥ Laboratory of Structural Chemistry and Biology, Institute of Chemistry, ELTE Eötvös Loránd University, Pázmány Péter Sétány 1/a, 1117 Budapest, Hungary

## Abstract

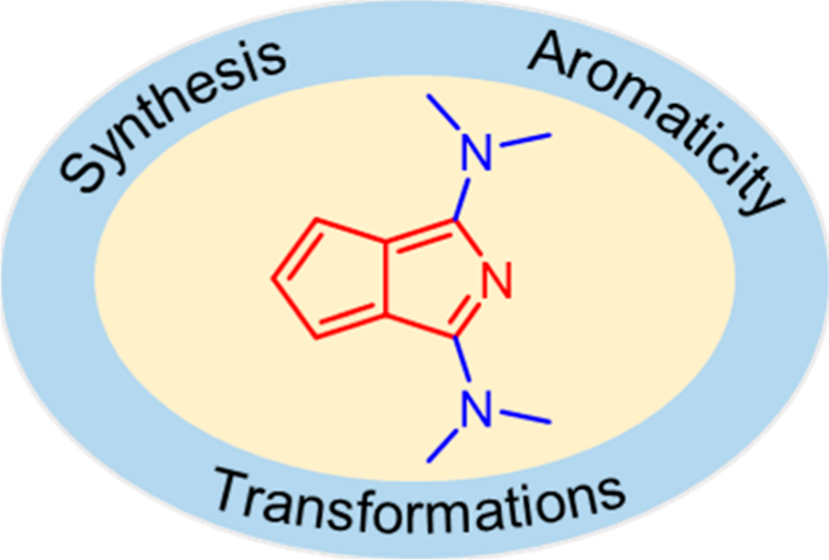

Stable azaheterocyclic derivatives of pentalene have been reported by the group of Hafner in the 1970s. However, these structures remained of low interest until recently, when they started to be investigated in the context of organic light-emitting diodes’ (OLEDs’) development. Herein, we revisit the synthesis of stable azapentalene derivative 1,3-bis(dimethylamino)-2-azapentalene and further explore its properties both computationally and experimentally. Beyond the reproduction and optimization of some previously reported transformations, such as formylation and amine substitution, the available scope of reactions was expanded with azo-coupling, selective halogenations, and cross-coupling reactions.

## Introduction

**Figure 1:**
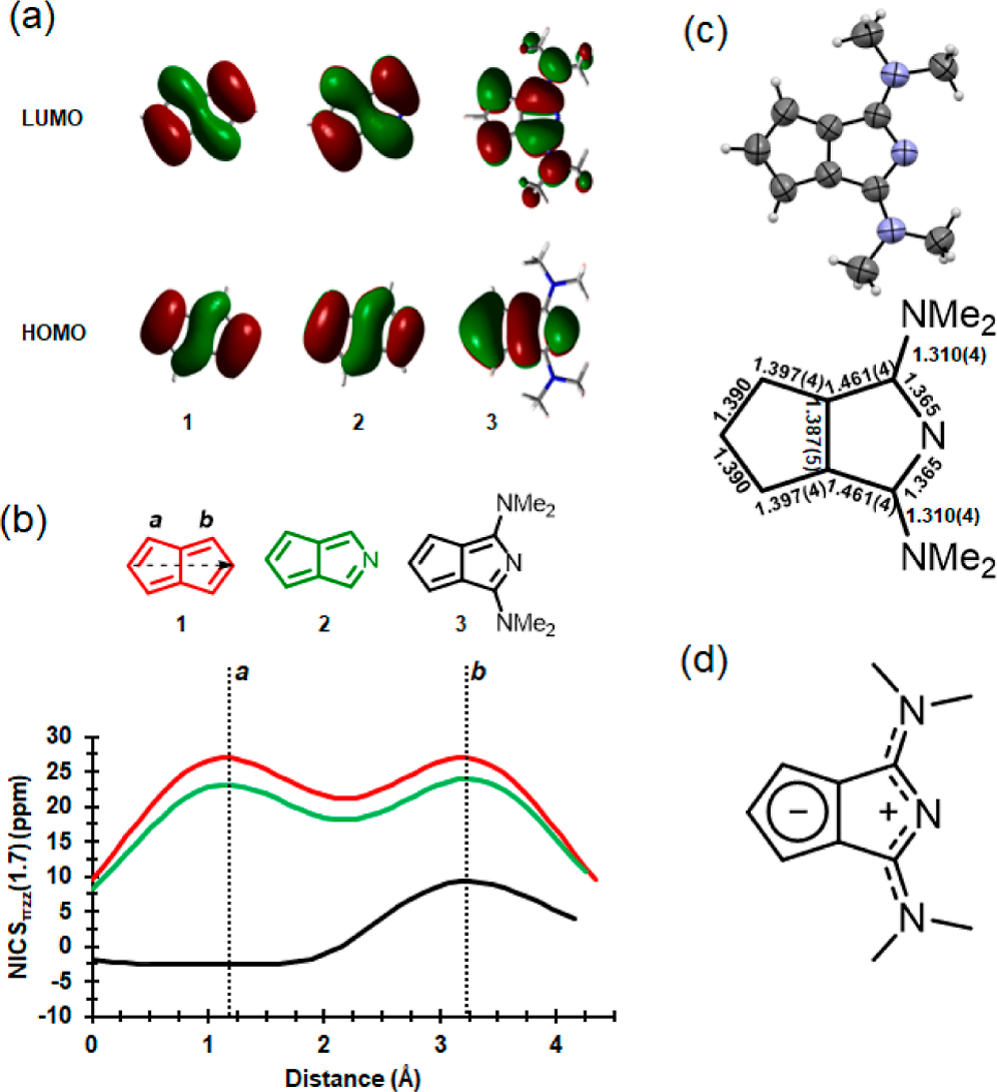
(a) Calculated HOMOs and LUMOs of **1**, **2**, and **3** (isosurface of 0.02 au is used). (b) NICS-XY scans and (c) X-ray structure of **3** and the corresponding bond lengths (Å) (ORTEP style representation is drawn at the 50% probability level). (d) Possible π-delocalized structure of **3**.

Polycyclic π-electron systems with nonbenzenoid subunits received considerable attention recently^1−6^ as tools for sharpening the fundamental understanding of conjugation effects and (anti)aromaticity rules.^7−12^ Along with fundamental studies, they opened a new chemical space for the design of advanced organic materials for optoelectronic applications.^13−17^ Klaus Hafner,^18^ a pioneer in the development of such systems,^19−21^ reported on several examples including azulenes,^22,23^*s*-indacenes,^24^ heptalenes,^25^ pentalenes,^26^ and some of their heterocyclic derivatives.^21^ Among these latter examples, azaazulenes received some attention,^28^ while azapentalenes remained neglected until recently, when they started to be investigated in the context of organic light emitting diodes’ (OLEDs’) development. Based on recent computational studies, azapentalenes and related materials might be interesting chromophores that exhibit singlet–triplet inversion (the lowest excited singlet state is below the energy of the lowest triplet state), which is beneficial for the development of next-generation OLEDs.^29,30^ Experimentally, so far, azapentalenes have been explored only in a limited number of studies. Simple azapentalenes, like 2-azapentalene (**2**), have not been isolated so far owing to their electronic similarity to the unstable parent carbocycle, pentalene (**1**) (Figure 1). However, Hafner and co-workers reported 2-azapentalene derivatives bearing electron donor groups (**3**) (Figure 1), which led to a strong stabilization of the 8π-electron framework.^27,31−34^ As only a few reports have appeared on this interesting structure, we set out to further explore the chemistry of stable azapentalene derivatives including both computational and synthetic approaches. Our goal is to highlight the properties and the reactivity through some model reactions of **3**, which might inspire further studies or lead to optoelectronic applications^29,30^ of this scaffold.

## Results and Discussion

In the following, we first present the optimized synthesis of azapentalene **3**, along with its structural and optoelectronic properties through X-ray crystallographic analysis, ^1^H NMR and UV–vis spectroscopic, and computational data. We then explored the reactivity of **3** with nucleophiles and electrophiles. The UV–vis absorption properties of the stable derivatives are also provided.

### Synthesis and Properties of Azapentalene **3**

The π-electron perimeter of azapentalene derivatives is isoelectronic with the parent carbocyclic pentalene.^35^ Indeed, on examination of the calculated frontier orbitals of azapentalene **2** (Figure 1a), the replacement of a C atom to a N heteroatom does not considerably influence the electronic system. Unlike pentalene (**1**) and 2-azapentalene (**2**), there is a cyclopentadienyl anion-type contribution to the highest occupied molecular orbital (HOMO) of **3**, which influences not only its stability but also affects the reactivity of the system. The different electron distribution in **3** contributes to a larger calculated S_0_ → S_1_ transition energy (2.46 eV) compared to pentalene (1.62 eV) and 2-azapentalene (1.77 eV) (B3LYP/6-311+G(d,p) in the CH_2_Cl_2_ solvent model; for further details, see Section S2 in the Supporting Information).

Notably, a similar substitution strategy renders pentalene itself stable as well, which was prepared by the group of Hafner,^36^ and recently new insights were reported on this structure^37^ (see also Section S2.3 in the Supporting Information). The electronic differences that underlie the stability and reactivity differences within the series are also reflected in the calculated (anti)aromaticity features of the molecules. In contrast to the strong global antiaromatic character of **1** and **2**, an NICS-XY scan^38,39^ reveals that in **3**, the 5-membered carbocycle is weakly aromatic and the heterocyclic 5-membered ring shows a weak antiaromatic character (Figure 1b). The calculated anisotropy of the induced current density (ACID) plots^40,41^ support these findings (see Section S2 in the Supporting Information). Furthermore, the harmonic oscillator model of aromaticity (HOMA)^42,43^ indicated a more preserved local aromaticity in the carbocyclic ring (0.837) compared to the heterocyclic ring (0.634) in **3**. For the π-electron perimeter of **3**, a HOMA value of 0.736 was obtained, which also supports the overall stability of the molecule. (The HOMA perimeter value for pentalene is −0.346, which is attributed to its antiaromatic character.)

**Figure 2:**
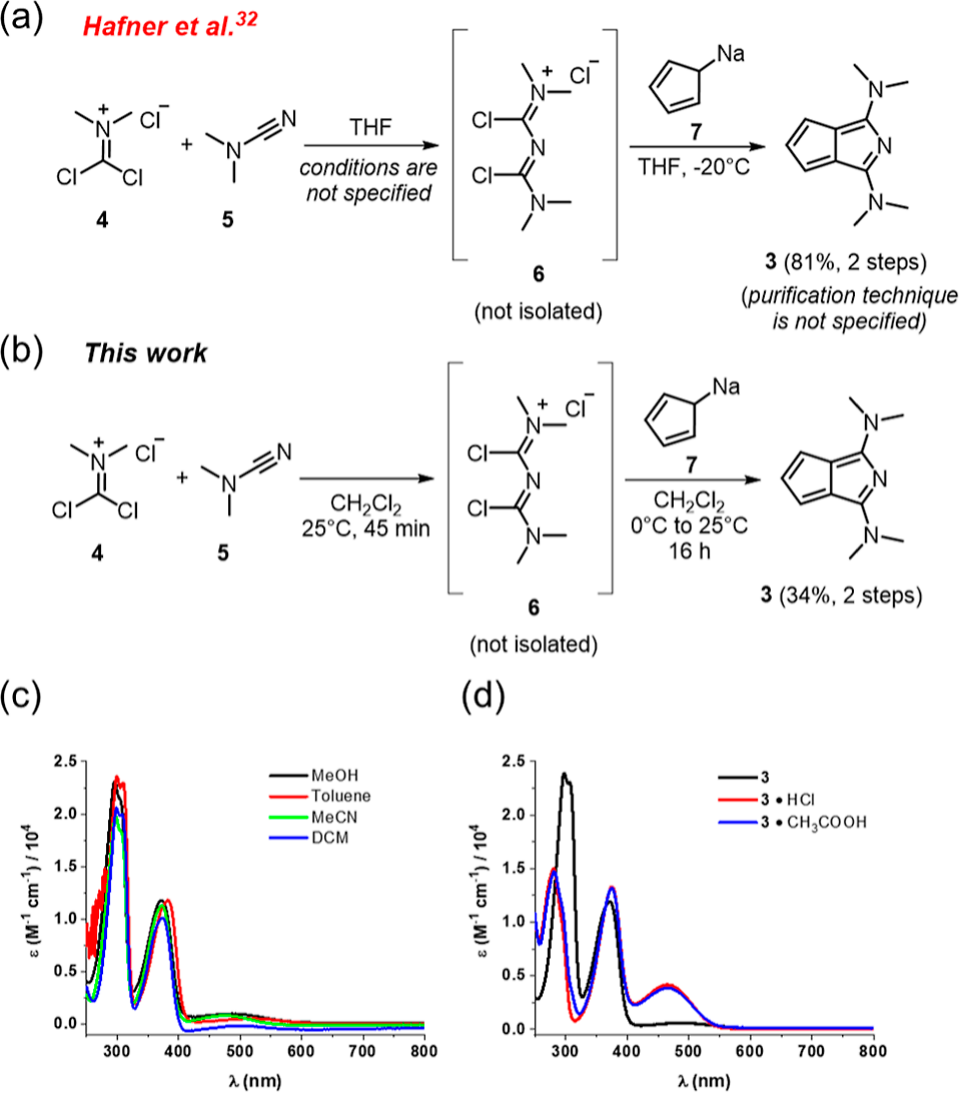
(a) Original synthesis of **3** reported by Hafner. (b) Optimized one-pot synthesis of **3**. (c) UV–vis spectra of **3** in different solvents at rt. (d) UV–vis spectra of **3** (CH_3_CN, rt) in the presence of acids.

For the synthesis of **3**, initially, we tried to follow the original report^32^ (Figure 2a) where the reaction conditions and the mode of purification were poorly detailed. Nevertheless, we attempted the reaction between **6** and **7** in line with ref (32); however, the solubility of **6** in THF at −20 °C was low, and no product could be isolated in this way. Alternatively, the formation of cyanin **6** from (dichloromethylene)dimethylammonium chloride (**4**) and dimethylcyanamide (**5**) and its subsequent reaction with **7** were carried out in CH_2_Cl_2_ at room temperature to improve the solubility of **6**. In this way, compound **3** was isolated as a bench-stable red solid in a moderate, 34% yield (Figure 2b). Regarding purification, it is noted that the reported vacuum sublimation at 150 °C^32^ is an applicable method to obtain pure product; however, we observed a considerable amount of black residue during this process, indicating thermal degradation of **3**. Column chromatography using basic alumina as the stationary phase was found to be the most efficient method for the purification of **3**.

In the ^1^H NMR spectrum (500 MHz, rt) of **3**, the chemical shifts of the Hs attached to the carbocyclic ring appeared at 6.19 and 6.05 ppm in CDCl_3_, 6.02 and 5.78 ppm in CD_3_CN, and 5.98 and 5.68 ppm in DMSO-*d*_6_. As a comparison, the chemical shifts of lithium cyclopentadienide^44^ are 5.51 and 5.32 ppm in CD_3_CN and DMSO-*d*_6_, respectively. The ^1^H NMR spectrum of **3** was also calculated, and among the different methods, the M11L functional^45^ provided the closest fit with the measured data (Table S3 in the Supporting Information). In the X-ray crystallographic structure of **3**, considerable bond-length equalization is found in the carbocycle (Figure 1c) that points toward a cyclopentadienyl (aromatic) character (Figure 1d). The equal bond lengths around the heterocyclic N support the presence of a charge-delocalized azacyanine unit (Figure 1d). The absorption spectrum of **3** was not substantially affected by the solvent used (Figure 2c). The spectrum displayed maxima around 500, 375, and 300 nm in each solvent. These absorptions could be assigned using time-dependent density functional theory (CH_2_Cl_2_ solvent model, for further details, see Section S2 in the Supporting Information) calculations as the HOMO → LUMO (*f* = 0.0049), the HOMO – 1 → LUMO (*f* = 0.2491), and the HOMO – 2 → LUMO (*f* = 0.5756) transitions. The obtained maximum at 499 nm in CH_2_Cl_2_ corresponds to an optical HOMO–LUMO gap of 2.48 eV, which agrees well with the calculated energy difference in the same solvent (2.46 eV). Azapentalene **3** can be protonated by the addition of acids such as HCl and AcOH, which results in the appearance of a band at 465 nm in the UV–vis spectrum (Figure 2d). An important practical aspect of protonation is that it strongly aids the purification of **3** and its derivatives as it leads to water-soluble salt formation in several cases. Our attempt to characterize the redox properties of **3** using cyclic voltammetry was unsuccessful, as the compound quickly degraded/polymerized under the measurement conditions (CH_2_Cl_2_, 0.1 M NBu_4_PF_6_, 0.25 V s^–1^ scan rate, glassy carbon electrode, Ar atmosphere).

### Reactivity of Azapentalene **3**

The electron density distribution induced by the N(CH_3_)_2_ groups determines the reactivity of **3**. Specifically, reactivity toward electrophiles is expected at the carbocyclic region but toward nucleophiles at the heterocyclic region.

**Scheme 1:**
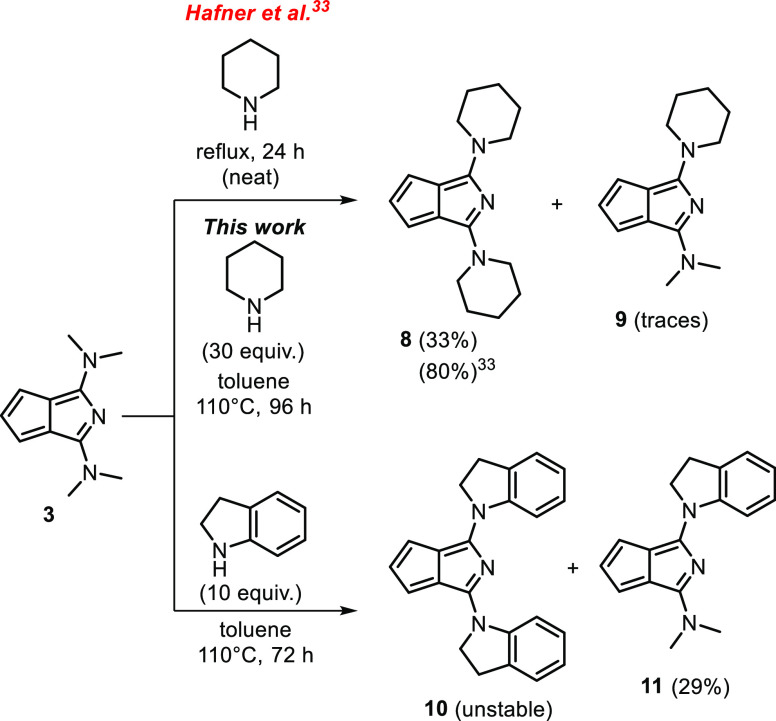
Nucleophilic Substitution Reactions of **3** with Amines

Nucleophilic substitution at the heterocyclic ring to exchange the dimethylamino groups have been described.^33^ Stable, piperidine-substituted 2-azapentalenes could be prepared in this way, which we also carried out under slightly modified conditions (Scheme 1). In the original synthesis of 1,3-bispiperidino-2-azapentalene (**8**), the heating of compound **3** in piperidine at 110 °C provided the product. This process indeed worked; however, we found the purification of compound **8** troublesome. By performing the reaction in toluene as the solvent using a large excess (30 equiv) of piperidine relative to **3**, compound **8** could be isolated in a moderate yield (33%). Furthermore, this process is expected to be applicable in cases where the reagent amine is a solid.

We attempted the extension of this chemistry to other amines, with moderate success. Indoline, having an aniline-type nitrogen, was reactive under similar conditions leading to a mixture of di- (**10**) and monosubstituted (**11**) derivatives. Upon purification, the disubstituted derivative repeatedly decomposed, while monoindoline derivative **11** was isolated and characterized. To optimize the synthesis of **11**, the reaction was repeated with only 10 equiv of indoline, which led to the isolation of **11** as a dark purple solid in 29% yield. The syntheses of compounds **8** and **11** were also carried out under microwave conditions, with similar results in shorter reaction times (6 h) (see Supporting Information). Upon using morpholine or piperazine and its derivatives (1-phenylpiperazine, 1-methylpiperazine, 1-(2-hydroxyethyl)piperazine) as the amine reagent, while the products could be detected by LC–MS, their isolation was not feasible due to decomposition.

**Scheme 2:**
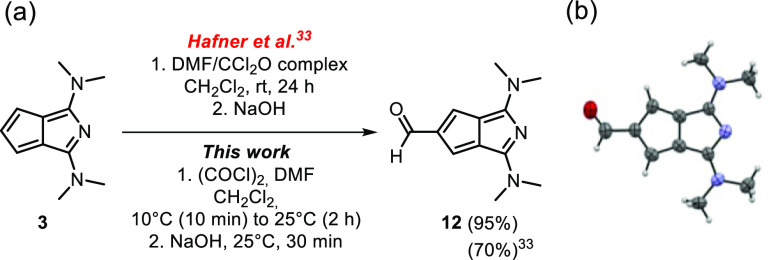
(a) Vilsmeier–Haack Formylation of **3**; (b) X-ray Structure of **12** ORTEP style representation is drawn at the 50% probability level, H atoms are omitted for clarity.

By addressing the reactivity of the carbocycle having aromatic character in **3** toward electrophiles, several new derivatives could be isolated, and the scope of tolerated reactions could be expanded compared to the initial work by Hafner and co-workers. The previously reported formyl azapentalene **12**(33,34) could be prepared (Scheme 2a). The earlier synthesis of **12** involved the Vilsmeier-complex as a reagent, which was prepared from phosgene and DMF in CHCl_3_ prior to the reaction. The reaction was carried out by the addition of a 1 M solution of the complex in CHCl_3_ to a solution of **3** in CH_2_Cl_2_ and subsequent stirring at rt for 24 h, providing **12** in 70% yield.^33^ By using a modified Vilsmeier–Haack formylation,^46^ we obtained formyl azapentalene **12** in an excellent yield (95%) as a bench-stable brownish-yellow solid, and its crystal structure could also be determined (Scheme 2b).

**Scheme 3:**
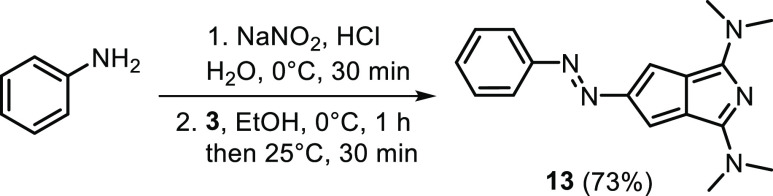
Synthesis of Azoazapentalene **13**

The reaction of **3** with benzenediazonium chloride provided the novel azoazapentalene **13** in good yield (73%) (Scheme 3) as an orange product, which slowly degraded at ambient conditions but could be stored at low temperature under an inert atmosphere. This product was formed exclusively as the monosubstituted derivative, and higher excess of the diazonium salt did not lead to multiply substituted derivatives.

**Scheme 4:**
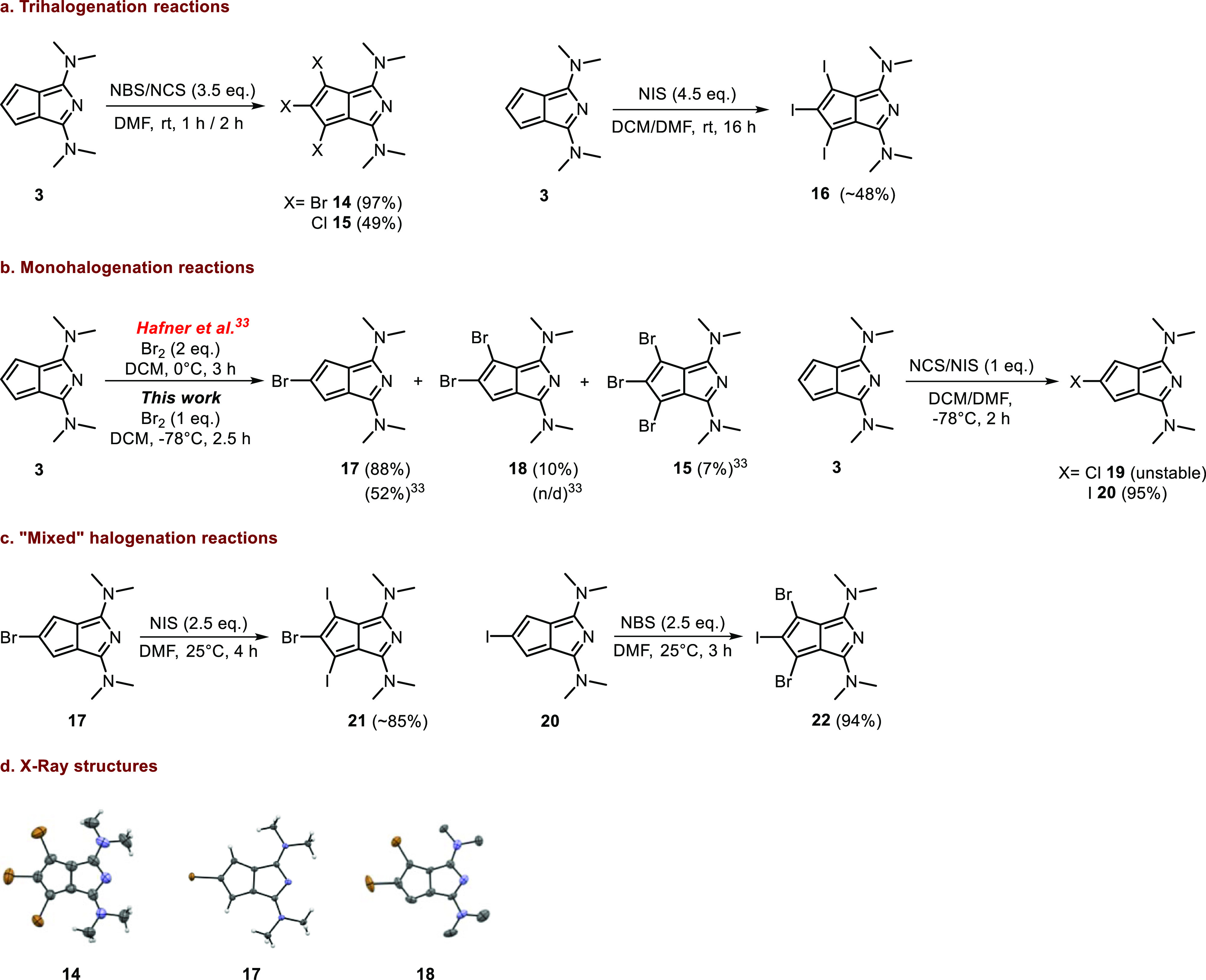
(a–c) Halogenation Reactions of Compound **3**; (d) X-ray Structures of Compounds **14**, **17**, and **18** ORTEP style representations are drawn at the 50% probability level, disordered H atoms are omitted for clarity.

Halogenation of the carbocycle of **3** could be an entry into its arylated and alkynylated derivatives through cross-coupling chemistries and could facilitate its integration into extended π-systems. Interestingly, maybe due to the less widespread applications of transition-metal-catalyzed coupling reactions in the early 1980s, Kläs and Hafner paid little attention to the halogenation of **3**. Their only attempt at bromination using Br_2_ led to a product mixture containing mono-, di-, and tribrominated derivatives, where the structure of the dibromo derivative could not be determined (Scheme 4b).^33^ Due to the synthetic importance of its halogenated derivatives, we systematically studied the selective halogenations of **3** (Scheme 4a–c). Initially, we used *N*-halosuccinimides (NXS) as halogenating agents. The reaction of **3** with NXS (1 equiv) in all cases (X = Br, Cl, I) led to the formation of product mixtures, while with excess reagent, the reactions could be driven toward the trihalogenated products (Scheme 4a). In the case of NBS, the tribrominated derivative **14** could be obtained in excellent yield (97%). Trichlorination was less efficient (49% yield), but the selectivity of the trisubstituted product **15** remained high while the conversion of **3** was lower. Additionally, under the basic workup, we observed some loss of product due to decomposition. Notably, under conditions similar to the bromination/chlorination with NXS, the triiodinated product (**16**) was not detected. When the reaction was carried out in a CH_2_Cl_2_/DMF solvent mixture, in which both NIS and the diiodinated product was soluble, triiodinated derivative **16** could be obtained. Unfortunately, **16** decomposed over time. Following trihalogenations, we turned to the synthesis of monohalogenated derivatives (Scheme 4b). As 1 equiv of NXS at rt led to product mixtures, we first adjusted the temperature to tune the selectivity. The reaction of **3** with NBS (0.7 equiv) in DMF at −35 °C (lower temperature led to the precipitation of **3**) provided a product mixture, similar to that in the rt reaction. Using NBS under different conditions (0.95 equiv, CH_2_Cl_2_/DMF, −78 °C, 2 h) led to the formation of the tribrominated compound **14** (30%) along with some di- and monobrominated derivatives (up to 5%). However, using Br_2_ and adjusting the reaction conditions (CH_2_Cl_2_, −78 °C), the monobrominated product **17** could be isolated in a high yield (88%) along with some dibrominated derivative **18** (10%). The structures of **17** and **18** could be determined crystallographically (Scheme 4d). The likely reason for this selectivity is the precipitation of the HBr salt of **17** upon the use of Br_2_, which prevents further incorporation of bromine. Further monohalogenated products could be obtained using NCS and NIS under low temperature in a CH_2_Cl_2_/DMF solvent mixture. Notably, the iodinated derivative **20** was isolable in excellent yield (95%), while the chlorinated compound **19** degraded during the workup and purification steps. The successful trihalogenation and monohalogenation processes provided an interesting perspective of the synthesis of derivatives containing different halogen atoms (Scheme 4c). The reactions, using NIS and NBS, respectively, for the transformations of **17** and **20**, were successful, leading to products **21** and **22** in high yields, although we observed degradation of **21** over time.

**Scheme 5:**
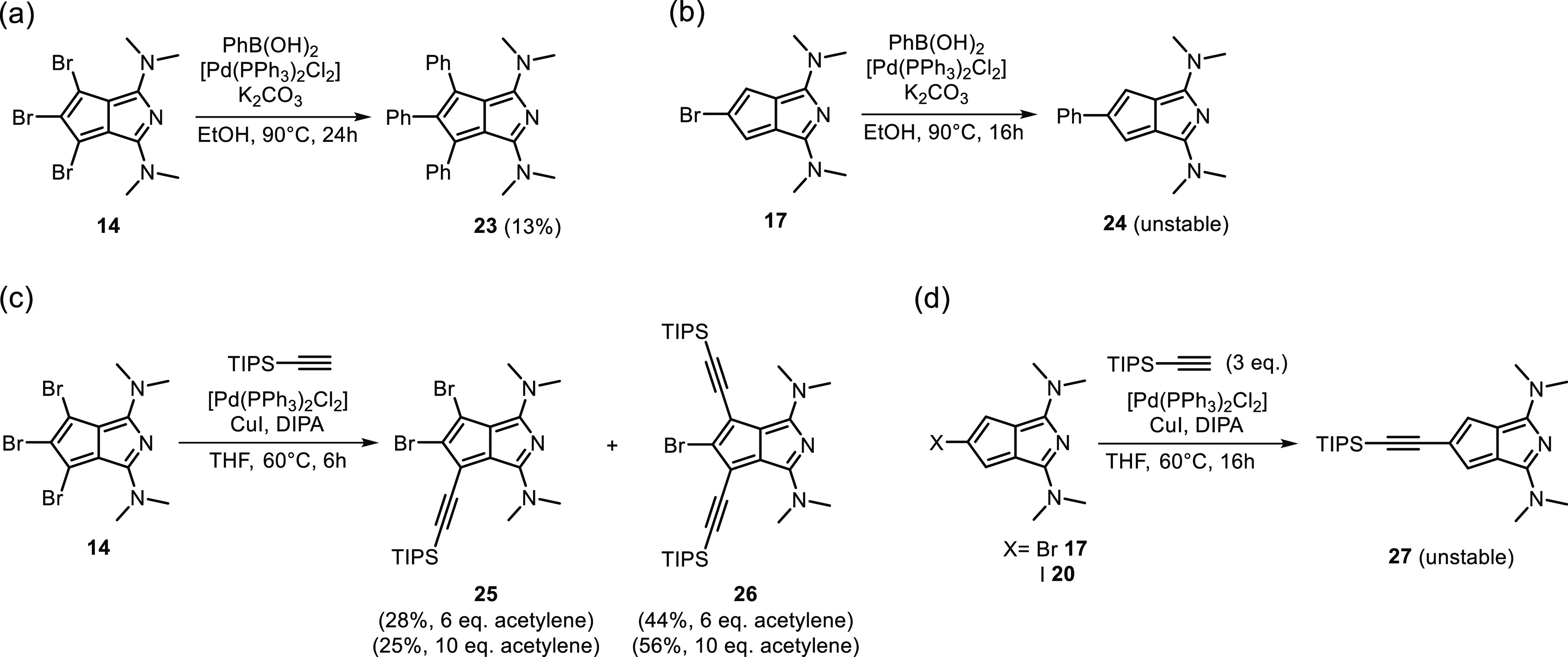
(a,b) Suzuki Reactions of Bromoazapentalenes; (c,d) Sonogashira Reactions of Different Haloazapentalenes

With the halogenated derivatives in hand, we probed their reactivity in model Suzuki and Sonogashira cross-coupling reactions (Scheme 5). Both the tribrominated (**14**) and the monobrominated (**17**) scaffolds were reactive in Suzuki coupling with phenylboronic acid in the presence of a Pd catalyst (Scheme 5a,b). In the former case, triphenyl azapentalene **23** was obtained as a stable material in low yield (13%). Under the applied conditions, disubstituted and dehalogenated products were also detected (by LC–MS) during the reaction. Monophenyl azapentalene **24** could be detected under similar reaction conditions; however, its isolation was not feasible due to rapid decomposition of the product.

The Sonogashira coupling of tribromo azapentalene **14** and monobromo azapentalene **17** with phenylacetylene was unsuccessful. While at lower temperatures (up to 50 °C) and shorter reaction times (up to 6 h) no conversion was detected, longer reaction times and higher temperature led to the disappearance of the starting material along with the formation of a complex and inseparable mixture of products, which did not contain the desired compounds. Nevertheless, by changing the acetylene reactant, productive couplings could be carried out. The reaction of **14** with TIPS-acetylene (6 equiv) led to stable mono- (**25**) and disubstituted (**26**) derivatives in low to moderate yields, while the triacetylene product was not detected (Scheme 5c). By increasing the TIPS-acetylene excess (10 equiv), compound **25** could be obtained in higher isolated yields (56%). The increased stability of some of the TIPS-acetylene derivatives compared to the phenylacetylene derivatives could be due to the steric shielding of the acetylene moiety by the bulky TIPS group that could prevent side-reactions of the triple bond.

The corresponding monofunctionalization, using either **17** or **20**, was not successful (Scheme 5d). Although the desired product (**27**) was detected in both cases (by LC–MS) during the reaction along with considerable amount of dehalogenated starting material, following the workup, it disappeared from the mixture. From the cross-coupling attempts, it seems that the monofunctionalized products are challenging substrates due to comparably low stability in most of the cases. In these derivatives, the remaining two reactive positions in the carbocyclic ring could be responsible for the decomposition. It is likely that protecting these positions will lead to more stable derivatives.

### Absorption Properties of the Stable Derivatives of **3**

**Figure 3:**
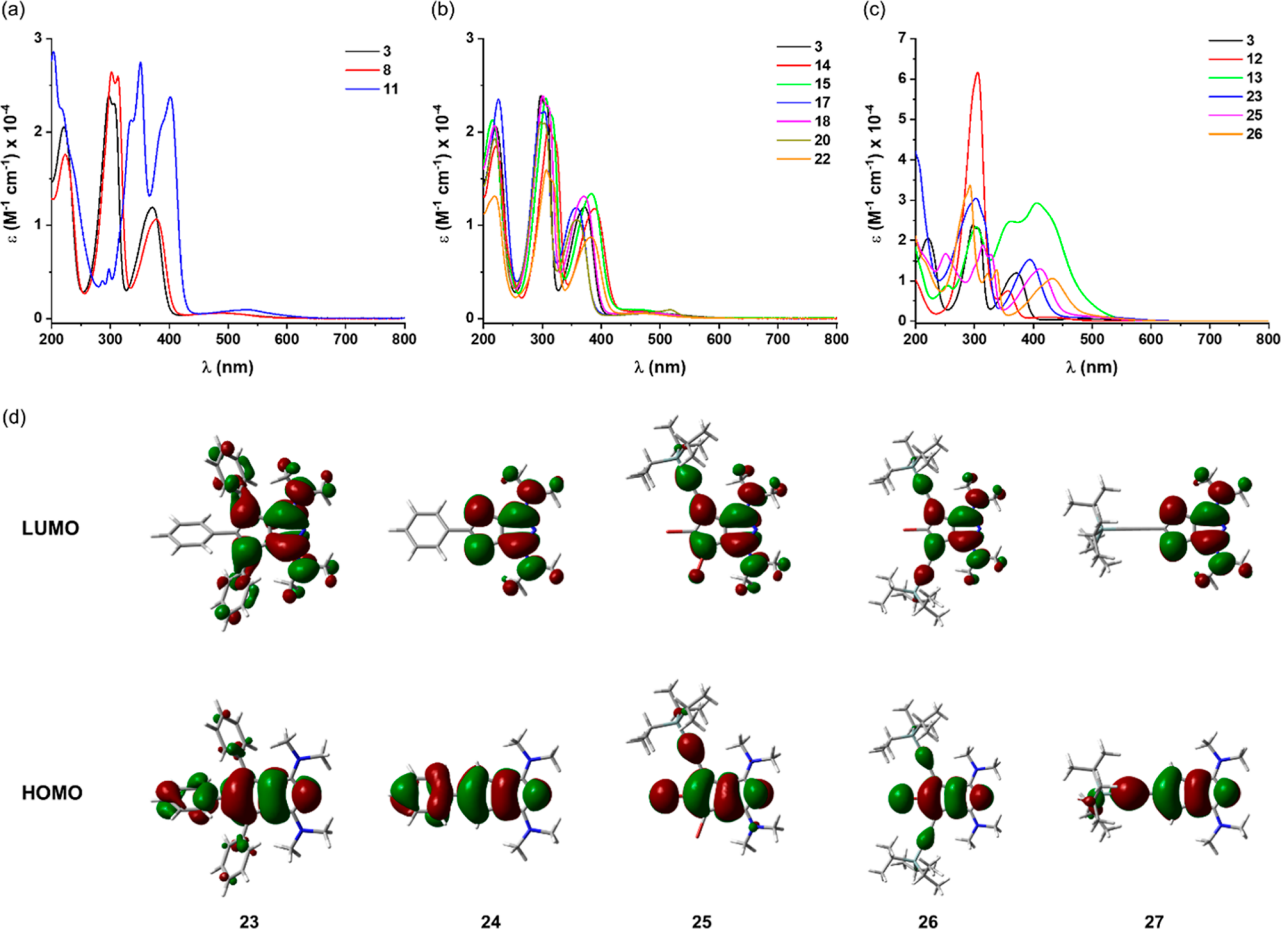
(a–c) UV–vis spectra (CH_3_CN, rt) of stable derivatives of **3**. (d) Calculated HOMOs and LUMOs of **23**–**27** (isosurface of 0.02 au is used).

We measured the UV–vis spectra of the stable derivatives of azapentalene **3** to explore the effect of the substituents on its optical properties. We first compared the NMe_2_-substituted derivatives (Figure 3a). Not surprisingly, the UV–vis spectrum of bis(piperidine) **8** was nearly identical to that of the parent compound **3**, while the introduction of the indoline substituent (**11**) considerably affected the spectrum. In this latter case, the absorption maxima shifted to longer wavelengths and their intensities increased compared to that of **3**, likely due to the conjugation with the aromatic amine. Among the halogenated derivatives (Figure 3b), the absorption maxima between 350 and 400 nm slightly red-shifted for those that contain three halogen substituents (**14**, **15**, **22**), while for those that contain one (**17**, **20**), they were slightly blue-shifted compared to **3**. The effect of the nonhalogen substituents on the carbocyclic ring was also studied (Figure 3c). The spectrum of aldehyde **12** showed similar features as that of **3**; however, the intensity of the maximum at 300 nm was considerably stronger, while the one at around 350 nm was weaker in this donor–acceptor type system. The spectrum of azoazapentalene **13** was dominated by the azophenyl chromophore and showed similarities to those of aminoazobenzenes. Stable products from the cross-coupling chemistries were also measured (Figure 3c). The longer wavelength absorptions of the triphenyl (**23**) and the TIPS-acetylene derivatives (**25**, **26**) red-shifted compared to that of **3**. The smallest shift was found for the triphenyl derivative **23** due to the noncoplanar phenyl groups, while it increased for **25** having a single and further increased for **26** having two TIPS-ethynyl groups in their structures.

As the cross-coupling products showed different stabilities, we looked at their calculated (B3LYP/6-311+G(d,p)) frontier orbitals (Figure 3d) for potential substituent effects and performed further NICS calculations. We found that the substituents in structures **23**–**27** did not considerably change the shapes of the HOMOs and lowest unoccupied molecular orbitals (LUMOs) on the azapentalene unit compared to that of unsubstituted **3** (Figure 3d). As a consequence, the calculated NICS-XY scans for these derivatives did not change much compared to that of **3** (Figure S11). Both the local weak aromatic character of the carbocycles and the local weak antiaromatic character of the heterocycles decreased slightly, leading to a somewhat stronger nonaromatic overall character. The calculated HOMO–LUMO energy gaps showed a notable difference between the highest value that was obtained for **3** (3.31 eV) and the lowest value obtained for **24** (2.98 eV). Notably, for the TIPS-ethynyl substituted isolable compounds **25** (Δ*E*_gap_ = 3.06 eV) and **26** (Δ*E*_gap_ = 2.99 eV), considerably lower LUMO levels were found compared to **3** (around 46% decrease for both **25** and **26**, see Table S6), which is a similar effect to what has been found for pentacenes.^47^ This effect could render **25** and **26** more stable toward photooxidation; however, it does not fully account for the increased stability of these derivatives. Compound **27**, with a single TIPS-ethynyl substituent, showed less pronounced decrease in its LUMO level (about 19% compared to the LUMO of **3**), while its HOMO–LUMO gap (3.02 eV) was similar to those of **25** and **26**. Compound **27** could not be isolated. On the other hand, triphenyl substituted **23** with a relatively low HOMO–LUMO gap (3.07 eV) and a weakly lowered LUMO level (15% compared to **3**) could be isolated and characterized. These findings suggest that apart from the electronic effects of individual substituents on the carbocycle, the number of substituents could also play a role in the stability of these compounds by preventing reactions on the free C–H sites of the molecules.

## Conclusions

In summary, we synthesized 1,3-bis(dimethylamino)-2-azapentalene **3** and characterized its structure and basic optoelectronic properties using both experimental techniques (^1^H NMR, X-ray crystallography, UV–vis spectroscopy) and computed aromaticity indices (NICS, ACID, HOMA). These revealed that the donor NMe_2_ groups play a key role in stabilizing the azapentalene π-system that otherwise has an antiaromatic character. Due to this electron donation, the heterocyclic ring of **3** becomes relatively electron-poor, while its carbocyclic ring is relatively electron-rich, which is also reflected in its reactivity, which we explored through model reactions. These included the reproduction and optimization of some previously reported transformations such as formylation and amine substitution and also the expansion of the previously known scope of reactions with azo-coupling, selective halogenations, and cross-coupling reactions.^48^ In future research, we plan to further expand the scope of stable derivatives of **3** and to incorporate it into π-extended frameworks and photoresponsive structures.

## Experimental Section

### General Information

Commercial reagents, solvents, and catalysts (Sigma-Aldrich, Fluorochem, and VWR) of reagent grade were purchased and used without further purification. Solvents for extraction or column chromatography were of a technical quality. The microwave reactions were carried out in an Anton Paar microwave synthesizer reactor type Microwave 300 in sealed reaction vessels. Organic solutions were concentrated by rotary evaporation at 40 °C. Thin-layer chromatography was carried out on “Merck silica gel 60 F_254_” or “Merck aluminum oxide 60 F_254_ neutral” type UV-active silica or alumina sheets. Column chromatography was performed using a Teledyne Isco CombiFlash Rf+ automated flash chromatographer with “RediSep R_f_ GOLD” silica gel or basic alumina column at 25(±1) °C. The cartridge was filled with Zeochem “ZEOprep 60 25–40 μm” silica gel or EcoChrom “MP Alumina B - Super I” basic alumina. Analytical RP-HPLC-UV/vis-MS measurements were carried out using a Shimadzu LCMS 2020 instrument applying a Gemini C18 column (100 mm × 2.00 mm I.D.) in which the stationary phase is 5 μm silica with a pore size of 110 Å. The chromatograms were recorded with a UV–vis diode array (190–800 nm) and an ESI-MS detector. The following linear gradient elution profile was applied for the LC–MS measurements: 0% → 100% B in 6.5 min then 100% → 0% B in 0.5 min, then 0% B for 1 min with eluents A (2% HCOOH, 5% CH_3_CN and 93% water) and B (2% HCOOH, 80% CH_3_CN and 18% water) at a flow rate of 0.8 mL/min at 40 °C. Preparative HPLC was performed on a ECOM HPLC system with “ECOM ECP2300” pumps, “TOY 14 DAD” detector, “ECOM S6021” injector, a TELEDYNE ISCO “Foxy R2” fraction picker, and a “GEMINI 5um NX C18 110 A” column (Product Number: 00G-4454-V0-AX). Room temperature refers to 25(±1) °C.

NMR spectra were acquired on a Varian 500 (^1^H 500 MHz, ^13^C 126 MHz) or a Varian 300 (^1^H 300 MHz, ^13^C 75 MHz) NMR spectrometer. The residual solvent peaks were used as the internal reference. Chemical shifts (δ) are reported in ppm. The following abbreviations are used to indicate the multiplicity in ^1^H NMR spectra: s, singlet; d, doublet; t, triplet; m, multiplet. ^13^C NMR spectra were acquired in a broadband decoupled mode. All NMR spectra were recorded at 30 °C.

UV–vis absorption spectroscopy was executed on a Jasco V-750 spectrophotometer. Data were collected from 700 to 200 nm using a 0.5 nm data interval and a 400 nm/s scan speed. Hellma Analytics high-precision quartz cuvettes were used with an optical path length of 1.0 cm. Spectra were recorded in 5 × 10^–5^ M solutions at 25(±1) °C. Baseline correction was used for each solvent.

High-resolution mass spectrometry measurements were performed on a Sciex TripleTOF 5600+ high-resolution tandem mass spectrometer equipped with a DuoSpray ion source. APCI or ESI ionization was applied in positive ion detection mode. Samples were dissolved in acetonitrile and flow injected into the acetonitrile/water 1:1 flow. The flow rate was 0.2 mL/min. The resolution of the mass spectrometer was 35,000.

#### Synthesis of Azapentalene **3**

*N*,*N*-Dimethylcyanamide (863 mg, 962 μL, 12.3 mmol) was added to a stirred suspension of (dichloromethylene)dimethylammonium chloride (2.00 g, 12.1 mmol) in dry CH_2_Cl_2_ (12 mL) under an inert atmosphere (N_2_) at room temperature. The reaction was stirred for 45 min until the colorless suspension became a yellowish solution. The solution was diluted with dry CH_2_Cl_2_ (150 mL) and cooled to 0 °C (ice/water bath). Subsequently, sodium cyclopentadienide (2.4 M in THF, 5.13 mL, 12.3 mmol) was added dropwise to the reaction at 0 °C. After the addition, the mixture was allowed to warm to room temperature and stirred for an additional 18 h. The resulting mixture was extracted with water (2 × 400 mL) and a 2% HCl solution (1 × 400 mL). To the combined aqueous phase, CH_2_Cl_2_ (200 mL) was added and the vigorously stirred mixture was treated with Na_2_CO_3_ in small portions until the pH of the aqueous phase turned basic. Subsequently, the organic phase was separated, and the aqueous phase was extracted with CH_2_Cl_2_ (3 × 300 mL). The combined organic phase was dried over MgSO_4_ and the solvent was evaporated under reduced pressure. The crude product was purified by column chromatography (basic alumina, eluent: CH_2_Cl_2_). The product (**3**) was obtained as a red, crystalline solid (791 mg, 34%).

^1^H NMR (500 MHz, CDCl_3_): δ 6.19 (d, *J* = 3.4 Hz, 2H), 6.05 (t, *J* = 3.4 Hz, 1H), 3.37 (s, 6H), 3.30 (s, 6H) ppm. ^1^H NMR (500 MHz, CD_3_CN): δ 6.02 (d, *J* = 3.4 Hz, 2H), 5.78 (t, *J* = 3.4 Hz, 1H), 3.26 (s, 6H), 3.21 (s, 6H) ppm. ^1^H NMR (500 MHz, DMSO-*d*_6_): δ 5.98 (d, *J* = 3.4 Hz, 2H), 5.68 (t, *J* = 3.4 Hz, 1H), 3.24 (s, 6H), 3.19 (s, 6H) ppm. ^13^C{^1^H} NMR (126 MHz, CDCl_3_): δ 170.4, 122.2, 118.3, 115.2, 39.9, 38.5 ppm. HRMS (ESI) *m*/*z*: [M + H]^+^ calcd for C_11_H_16_N_3_^+^, 190.1338; found, 190.1338.

#### Synthesis of Compound **3**·HCl

Concentrated hydrochloric acid (52.1 mg, 43.4 μL, 528 μmol, 37%) was added to a stirred solution of azapentalene **3** (50.0 mg, 264 μmol) in CH_3_CN (5.0 mL) and stirred for a further 1 min, after which it was completed. The reaction mixture was dried over MgSO_4_, filtered, and the solvent was removed under a vacuum. The product (**3**·HCl) was obtained quantitatively as an orange solid (59.5 mg, 100%).

^1^H NMR (500 MHz, CD_3_CN): δ 11.22 (s, 1H), 6.60 (d, *J* = 3.6 Hz, 2H), 6.35 (t, *J* = 3.6 Hz, 1H), 3.68 (s, 6H), 3.40 (s, 6H) ppm. ^13^C{^1^H} NMR (126 MHz, DMSO-*d*_6_/D_2_O = 4:1): δ 156.7, 122.5, 116.7, 42.9, 41.9 ppm.

#### Synthesis of Compound **8**

##### Method A

Azapentalene **3** (50.0 mg, 264 μmol) and piperidine (675 mg, 783 μL, 7.93 mmol) were dissolved in abs. toluene (3.0 mL), and the mixture was stirred at 110 °C (in an aluminum heating block) for 4 d. The reaction was monitored with LC–MS. When the reaction was completed, the volatiles were removed under reduced pressure. The residue was dried further for 1 h at 2 mbar pressure at 40 °C. The resulting dark viscous liquid was dissolved in CH_2_Cl_2_ (150 mL) and washed with NaHCO_3_ (1 × 100 mL). The organic phase was dried over MgSO_4_, and the solvent was evaporated in vacuo. The crude product was purified by column chromatography [basic Al_2_O_3_, *n*-hexane → *n*-hexane/EtOAc (70%)]. The product (**8**) was obtained as a dark purple solid (23 mg, 33%).

##### Method B

Azapentalene **3** (50.0 mg, 264 μmol) and piperidine (675 mg, 783 μL, 7.93 mmol) were dissolved in abs. toluene (3.0 mL), and the mixture was stirred in a MW reactor at 200 °C for 6 h in a sealed reaction vessel. Workup and purification steps were identical to those in Method A (40 mg, 57%).

^1^H NMR (500 MHz, CDCl_3_): δ 6.17 (d, *J* = 3.4 Hz, 2H), 6.03 (t, *J* = 3.4 Hz, 1H), 3.88 (t, *J* = 5.0 Hz, 4H), 3.75 (t, *J* = 4.6 Hz, 4H), 1.76–1.66 (m, 12H) ppm. ^13^C{^1^H} NMR (126 MHz, CDCl_3_): δ 168.6, 121.6, 117.9, 113.7, 49.2, 47.1, 47.1, 26.0, 26.0, 24.2 ppm. HRMS (ESI) *m*/*z*: [M + H]^+^ calcd for [C_17_H_24_N_3_]^+^, 270.1965; found, 270.1971.

#### Synthesis of Compound **11**

##### Method A

Azapentalene **3** (100 mg, 528 μmol) was dissolved in abs. toluene (6.0 mL) and indoline (594 μL, 5.28 mmol; filtered through a pad of silica prior to reaction to remove potential impurities) was added. The resulting mixture was stirred at 110 °C (in an aluminum heating block) for 3 days. The reaction was monitored with LC–MS. When the reaction was completed, the solvent was evaporated under reduced pressure. The crude product was a dark reddish liquid, which was purified by column chromatography (SiO_2_, *n*-hexane → *n*-hexane/EtOAc (20%)). The product (**11**) was obtained as a dark purple solid (41 mg, 29%).

##### Method B

Compound **3** (50.0 mg, 264 μmol) was dissolved in abs. toluene (3.0 mL) and indoline (891 μL, 7.93 mmol; filtered through a pad of silica prior to reaction to remove potential impurities) was added, and the mixture was stirred in a MW reactor at 200 °C for 6 h in a sealed reaction vessel. Workup and purification steps were identical to those in Method A (18.8 mg, 27%)

^1^H NMR (500 MHz, CD_2_Cl_2_): δ 8.45 (d, *J* = 8.1 Hz, 1H), 7.27–7.20 (m, 2H), 7.06 (t, *J* = 7.4 Hz, 1H), 6.17 (d, *J* = 3.1 Hz, 1H), 6.09 (d, *J* = 3.2 Hz, 1H), 5.89 (t, *J* = 3.3 Hz, 1H), 4.23 (t, *J* = 8.3 Hz, 2H), 3.41 (s, 3H), 3.32–3.27 (m, 5H) ppm. ^13^C{^1^H} NMR (126 MHz, CD_2_Cl_2_): δ 170.9, 166.0, 143.6, 134.1, 128.0, 125.4, 125.0, 124.9, 121.2, 119.3, 119.3, 118.1, 116.9, 50.9, 40.4, 39.3, 28.4 ppm. HRMS (ESI) *m*/*z*: [M + H]^+^ calcd for C_17_H_18_N_3_^+^, 264.1496; found, 264.1504.

#### Synthesis of Compound **12**

Oxalyl chloride (416 μL, 4.76 mmol) was added dropwise to a vigorously stirred mixture of dry DMF (368 μL, 4.76 mmol) and CH_2_Cl_2_ (8.0 mL) at 10 °C over 10 min. Subsequently, azapentalene **3** (450 mg, 2.38 mmol) in CH_2_Cl_2_ (15 mL) was added to the solution dropwise at room temperature under an inert atmosphere (N_2_), and the mixture was stirred for 2 h. Afterward, 1 M NaOH solution (50 mL) was added and the reaction was stirred for 30 min. The resulting mixture was diluted with CH_2_Cl_2_ (300 mL) and the separated organic phase was washed with saturated NaHCO_3_ (2 × 200 mL). The organic phase was dried over MgSO_4_ and the solvent was evaporated under reduced pressure. The product was purified by column chromatography [SiO_2_, CH_2_Cl_2_ + 0.5% TEA → CH_2_Cl_2_/CH_3_OH (10%) + 0.5% TEA]. The product (**12**) was obtained as a yellowish-brown crystalline solid (490.4 mg, 95%).

^1^H NMR (500 MHz, CDCl_3_): δ 9.54 (s, 1H), 6.73 (s, 2H), 3.37 (d, *J* = 4.3 Hz, 12H) ppm. ^13^C{^1^H} NMR (126 MHz, CDCl_3_): δ 186.1, 170.4, 135.6, 40.1, 38.9 ppm. (The signals corresponding to the 4 quaternary carbon atoms of the heterocyclic ring were not observed in the ^13^C NMR spectrum due to low intensity.) HRMS (ESI) *m*/*z*: [M + H]^+^ calcd for C_12_H_16_N_3_O^+^, 218.1987; found, 218.1985.

#### Synthesis of Compound **13**

Aniline (18.6 mg, 200 μmol) was dissolved in a mixture of concentrated hydrochloric acid (100 μL) and water (2.0 mL). A solution of NaNO_2_ (18.4 mg, 264 μmol) in water (1.0 mL) was added dropwise while keeping the temperature at 0–5 °C (ice/water bath). After the solution was stirred at 0–5 °C for 30 min, the diazonium salt solution was added to an ice-cold mixture of azapentalene **3** (37.9 mg, 200 μmol) and NaOAc (49.2 mg, 600 μmol) in EtOH (2.0 mL). Following a further 1 h of stirring at 0–5 °C, the mixture was allowed to warm to room temperature over 1 h and a 0.5 M solution of hydrochloric acid (20 mL) was added. The resulting aqueous mixture was washed with EtOAc (2 × 20 mL). Subsequently, the pH of the separated aqueous phase was adjusted to pH > 8 with 1 M NaOH solution. The resulting suspension was extracted with CH_2_Cl_2_ (2 × 20 mL), and the combined organic layer was dried over MgSO_4_. The solvent was removed by rotary evaporation, and the crude product was obtained as a dark reddish solid. The crude product was purified by preparative HPLC (SiO_2_–C18, eluent A: water/CH_3_CN = 95/5 + HCOOH 0,1%, eluent B: CH_3_CN + HCOOH 0,1%, gradient: eluent A → eluent B in 40 min). The product (**13**) was obtained as a red solid (43 mg, 73%).

^1^H NMR (500 MHz, CD_2_Cl_2_): δ 7.69–7.63 (m, 2H), 7.40 (t, *J* = 7.8 Hz, 2H), 7.24 (t, *J* = 7.3 Hz, 1H), 6.74 (s, 2H), 3.38 (s, 12H) ppm. ^13^C{^1^H} NMR (126 MHz, CD_2_Cl_2_): δ 171.3, 153.4, 129.3, 127.7, 121.7, 40.5, 39.3 ppm. (The signals corresponding to 2 quaternary carbon atoms of the heterocyclic ring were not observed in the ^13^C NMR spectrum due to low intensity.) HRMS (APCI) *m*/*z*: [M + H]^+^ calcd for C_17_H_20_N_5_^+^, 294.1713; found, 294.1714.

#### Synthesis of Compound **14**

*N*-Bromosuccinimide (846 mg, 4.75 mmol) was added in small portions to a stirred solution of azapentalene **3** (300 mg, 1.58 mmol) in DMF (25 mL) at room temperature. The reaction was monitored with LC–MS. After 2 h, the reaction was completed, and the solvent was evaporated in vacuo. The residue was dissolved in CH_2_Cl_2_ (250 mL) and was washed with 1 M NaOH (3 × 200 mL). The organic phase was dried over MgSO_4_, and the solvent was evaporated under reduced pressure. The crude product was purified by column chromatography (SiO_2_, CH_2_Cl_2_ → CH_2_Cl_2_/CH_3_OH 10%). The product (**14**) was obtained as a red crystalline solid (656 mg, 97%).

^1^H NMR (500 MHz, CDCl_3_): δ 3.63 (s, 6H), 3.26 (s, 6H) ppm. ^13^C{^1^H} NMR (126 MHz, CD_2_Cl_2_): δ 168.3, 120.6, 109.5, 99.2, 43.5, 40.6 ppm. HRMS (ESI) *m*/*z*: [M + H]^+^ calcd for C_11_H_13_N_3_Br_3_^+^, 423.8654; found, 423.8667.

#### Synthesis of Compound **15**

*N*-Chlorosuccinimide (370 mg, 2.77 mmol) was added in small portions to a stirred solution of azapentalene **3** (150 mg, 1.58 mmol) in DMF (10 mL) at room temperature. The reaction was monitored with LC–MS. After 1 h, the reaction was completed, and the solvent was evaporated under reduced pressure. The residue was dissolved in CH_2_Cl_2_ (250 mL) and was washed with 1 M NaOH (3 × 200 mL). The organic phase was dried over MgSO_4_, and the solvent was evaporated under reduced pressure. The crude product was purified by column chromatography [SiO_2_, CH_2_Cl_2_ → CH_2_Cl_2_/CH_3_OH (10%)]. The product (**15**) was obtained as a red crystalline solid (114 mg, 49%).

^1^H NMR (500 MHz, CDCl_3_): δ 3.60 (s, 6H), 3.32 (s, 6H) ppm. ^13^C{^1^H} NMR (126 MHz, CDCl_3_): δ 167.5, 114.6, 110.2, 42.9, 40.6 ppm. HRMS (ESI) *m*/*z*: [M + H]^+^ calcd for C_11_H_13_N_3_Cl_3_^+^, 292.0169; found, 292.0176.

#### Synthesis of Compound **16**

*N*-Iodosuccinimide (267 mg, 1.19 mmol) was added in small portions to a stirred solution of azapentalene **3** (50.0 mg, 264 μmol) and DMF (614 μL) in CH_2_Cl_2_ (7.0 mL) at room temperature. The reaction was monitored with LC–MS. After 16 h, the reaction was completed, and the solvent was evaporated under reduced pressure. The residue was dissolved in CH_2_Cl_2_ (100 mL) and the solution was washed with 5% NaOH (3 × 50 mL). The organic phase was dried over MgSO_4_, and the solvent was evaporated under reduced pressure. The crude product was purified by column chromatography [SiO_2_, CH_2_Cl_2_ → CH_2_Cl_2_/CH_3_OH (10%)]. The product was obtained as a red crystalline material (72.1 mg, 48%) that decomposed over time.

^1^H NMR (500 MHz, CDCl_3_): δ 3.74 (s, 6H), 3.25 (s, 6H) ppm. ^13^C NMR could not be obtained due to decomposition. HRMS (APCI) *m*/*z*: [M + H]^+^ calcd for C_11_H_13_N_3_I_3_^+^, 567.8243; found, 567.8224.

#### Synthesis of Compound **17**

Bromine (285 mg, 92.0 μL, 1.79 mmol) in CH_2_Cl_2_ (2 mL) was added dropwise to a stirred solution of azapentalene **3** (338 mg, 1.79 mmol) in CH_2_Cl_2_ (13 mL) at −78 °C (acetone/dry ice) under an inert atmosphere (N_2_). After stirring the mixture for 2 h at −78 °C, it was allowed to warm to room temperature and stirred for an additional 30 min. The reaction was monitored by LC–MS. The mixture was diluted with CH_2_Cl_2_ (250 mL) and washed with saturated Na_2_S_2_O_3_ (1 × 100 mL), saturated NaHCO_3_ (1 × 100 mL), and water (1 × 100 mL). The organic phase was dried over MgSO_4_, and the solvent was evaporated in vacuo. The crude product was purified by column chromatography [SiO_2_, CH_2_Cl_2_ → CH_2_Cl_2_/CH_3_OH (5%))]. The product (**17**) was obtained as a red crystalline solid (424 mg, 88%).

^1^H NMR (300 MHz, CDCl_3_): δ 6.12 (s, 2H), 3.34 (s, 6H), 3.26 (s, 6H) ppm. ^13^C{^1^H} NMR (126 MHz, CDCl_3_): δ 170.5, 120.9, 115.0, 101.8, 39.9, 38.8 ppm. HRMS (ESI) *m*/*z*: [M + H]^+^ calcd for C_11_H_15_N_3_Br^+^, 268.0443; found, 268.0446.

#### Synthesis of Compound **18**

This compound was obtained as a byproduct in the synthesis of compound **17**. The structure of **18** was assigned by 2D NMR techniques (HSQC, HMBC, and COSY, see the Supporting Information) and by X-ray crystallography.

^1^H NMR (300 MHz, CDCl_3_): δ 6.15 (s, 1H), 3.69 (s, 3H), 3.31 (s, 6H), 3.22 (s, 3H) ppm. ^13^C{^1^H} NMR (126 MHz, CDCl_3_): δ 170.0, 168.5, 122.7, 118.2, 115.3, 105.5, 99.7, 43.3, 40.3, 39.7, 38.9 ppm. HRMS (ESI) *m*/*z*: [M + H]^+^ calcd for C_11_H_14_N_3_Br_2_^+^, 347.9549; found, 347.9556.

#### Synthesis of Compound **20**

A solution of *N*-iodosuccinimide (56.5 mg, 251 μmol) and DMF (100 μL) in CH_2_Cl_2_ (2.0 mL) was added dropwise to a stirred solution of azapentalene **3** (50.0 mg, 264 μmol) in CH_2_Cl_2_ (6.0 mL) at −78 °C under an inert atmosphere (N_2_). The reaction was monitored with LC–MS. After 2 h, the reaction was completed and was allowed to warm to room temperature. The reaction mixture was diluted with CH_2_Cl_2_ (100 mL) and washed with 1 M NaOH (3 × 100 mL). The organic phase was dried over MgSO_4_ and the solvent was evaporated under reduced pressure. The crude product was purified by column chromatography [SiO_2_, eluent: CH_2_Cl_2_ → CH_2_Cl_2_/CH_3_OH (5%)]. The product (**20**) was obtained as a red crystalline solid (79.3 mg, 95%).

^1^H NMR (500 MHz, CD_2_Cl_2_): δ 6.14 (s, 2H), 3.31 (s, 6H), 3.23 (s, 6H) ppm. ^13^C{^1^H} NMR (126 MHz, CD_2_Cl_2_): δ 170.1, 123.5, 120.5, 66.7, 40.3, 38.9 ppm. HRMS (ESI) *m*/*z*: [M + H]^+^ calcd for C_11_H_15_N_3_I^+^, 316.0305; found, 316.0309.

#### Synthesis of Compound **21**

*N*-Iodosuccinimide (105 mg, 466 μmol) was added in small portions to a stirred solution of monobromo-azapentalene **17** (50.0 mg, 186 μmol) and DMF (700 μL) in CH_2_Cl_2_ (4.0 mL) at room temperature. The reaction was monitored with LC–MS. After 3 h, the reaction was completed, and the solvent was evaporated under reduced pressure. The residue was dissolved in CH_2_Cl_2_ (100 mL) and was washed with 5% NaOH solution (2 × 50 mL). The organic phase was dried over MgSO_4_ and the solvent was evaporated under reduced pressure. The crude product was purified by column chromatography [SiO_2_, CH_2_Cl_2_ → CH_2_Cl_2_/CH_3_OH (5%) + 0.5% TEA]. The product (**21**) was obtained as a red crystalline material (82,1 mg, 85% by ^1^H NMR) that degrades over time.

^1^H NMR (500 MHz, CDCl_3_): δ 3.74 (d, *J* = 2.7 Hz, 3H), 3.66 (d, *J* = 2.6 Hz, 3H), 3.29–3.22 (m, 6H) ppm. ^13^C{^1^H} NMR spectra could not be obtained due to degradation. HRMS (APCI) *m*/*z*: [M + H]^+^ calcd for C_11_H_13_N_3_BrI_2_^+^, 519.8382; found, 519.8371.

#### Synthesis of Compound **22**

*N*-Bromosuccinimide (64.9 mg, 365 μmol) was added in small portions to a stirred solution of monoiodo-azapentalene **20** (46.0 mg, 146 μmol) and DMF (700 μL) in CH_2_Cl_2_ (4.0 mL) at room temperature. The reaction was monitored with LC–MS. After 3 h, the reaction was completed, and the solvent was evaporated in vacuo. The residue was dissolved in CH_2_Cl_2_ (100 mL) and washed with 5% NaOH solution (2 × 50 mL). The organic phase was dried over MgSO_4_ and the solvent was evaporated under reduced pressure. The crude product was purified by column chromatography [SiO_2_, CH_2_Cl_2_ → CH_2_Cl_2_/CH_3_OH (5%) + 0.5% TEA]. The product (**22**) was obtained as a red crystalline solid (65.0 mg, 94%).

^1^H NMR (500 MHz, CDCl_3_): δ 3.66 (s, 6H), 3.28 (s, 6H) ppm. ^13^C{^1^H} NMR (126 MHz, CDCl_3_): δ 168.2, 120.5, 99.4, 43.5, 40.5 ppm. HRMS (APCI) *m*/*z*: [M + H]^+^ calcd for C_11_H_13_N_3_Br_2_I^+^, 471.8515; found, 471.8511.

#### Synthesis of Compound **23**

A mixture of tribromo-azapentalene **14** (300 mg, 704 μmol), phenylboronic acid (472 mg, 3.87 mmol), potassium carbonate (584 mg, 4.22 mmol), and Pd(PPh_3_)_2_Cl_2_ (24.7 mg, 35.2 μmol) in dry ethanol (18 mL) was stirred under an inert atmosphere (N_2_) at 90 °C in an aluminum heating block. The reaction was monitored with LC–MS. After 24 h, the reaction was completed, and the solvent was evaporated under reduced pressure. The residue was suspended in CH_2_Cl_2_ (200 mL), filtered, and washed with further portions of CH_2_Cl_2_. The organic filtrate was washed with water (3 × 150 mL) and dried over MgSO_4_ and the solvent was evaporated in vacuo. The crude product was purified by preparative HPLC [SiO_2_–C18, water → water/CH_3_CN (30%) + 0.1% HCOOH]. The product (**23**) was obtained as a brown solid (38.4 mg, 13%).

^1^H NMR (500 MHz, CD_2_Cl_2_): δ 7.16–7.09 (m, 10H), 6.90–6.85 (m, 3H), 6.74–6.70 (m, 2H), 3.26 (s, 6H), 2.61 (s, 6H) ppm. ^13^C{^1^H} NMR (126 MHz, CDCl_3_): δ 170.8, 139.9, 131.7, 131.5, 129.0, 126.9, 126.8, 125.8, 124.1, 120.6, 42.5, 39.8 ppm. HRMS (ESI) *m*/*z*: [M + H]^+^ calcd for C_29_H_28_N_3_^+^, 418.2277; found, 418.2263.

#### Synthesis of Compound **25**

Tribromo-azapentalene **14** (80.0 mg, 188 μmol), (triisopropylsilyl)acetylene (115 mg, 141 μL, 1.13 mmol), Pd(PPh_3_)_2_Cl_2_ (6.59 mg, 9.39 μmol), and copper(I) iodide (1.79 mg, 9.39 μmol) were dissolved in THF (5.0 mL). Subsequently, diisopropylamine (2.0 mL) was added dropwise while being stirred under an inert atmosphere (N_2_). The mixture was stirred for 24 h at 60 °C in an aluminum heating block. Afterward, the reaction mixture was diluted with EtOAc (50 mL) and filtered through a pad of Celite. The solvent was evaporated under reduced pressure. The crude product was purified by column chromatography [SiO_2_, *n*-hexane → *n*-hexane/EtOAc (30%)]. The product (**25**) was obtained as a brownish-red solid (28.0 mg, 28%).

^1^H NMR (500 MHz, CDCl_3_): δ 3.74 (s, 3H), 3.69 (s, 3H), 3.30 (d, *J* = 2.9 Hz, 6H), 1.10 (s, 21H) ppm. ^13^C{^1^H} NMR (75 MHz, CDCl3): δ 104.9, 43.6, 42.7, 40.7, 39.9, 18.9, and 11.7 ppm. HRMS (ESI) *m*/*z*: [M + H]^+^ calcd for C_22_H_34_N_3_Br_2_Si^+^, 526.0883; found, 526.0873.

#### Synthesis of Compound **26**

Tribromo-azapentalene **14** (150 mg, 352 μmol), (triisopropylsilyl)acetylene (642 mg, 790 μL, 3.52 mmol), Pd(PPh_3_)_2_Cl_2_ (12.4 mg, 17.6 μmol), and copper(I) iodide (3.35 mg, 17.6 μmol) were dissolved in THF (10 mL) and stirred under an inert atmosphere (N_2_). Subsequently, diisopropylamine (2.0 mL) was added dropwise to the mixture, and it was stirred for 24 h at 60 °C in an aluminum heating block. Afterward, the reaction mixture was diluted with EtOAc (100 mL) and filtered through a pad of Celite, and then the solvent was evaporated under reduced pressure. The crude product was purified by column chromatography [SiO_2_, *n*-hexane → *n*-hexane/EtOAc (30%)]. The product (**26**) was obtained as a brownish-red solid (124 mg, 56%).

^1^H NMR (500 MHz, CDCl_3_): δ 3.75 (s, 6H), 3.31 (s, 6H), 1.13–1.07 (m, 42H) ppm. ^13^C{^1^H} NMR (126 MHz, CDCl_3_): δ 168.9, 121.3, 118.6, 107.6, 96.5, 42.5, 39.7, 18.9, 11.7 ppm. HRMS (ESI) *m*/*z*: [M + H]^+^ calcd for C_33_H_55_N_3_BrSi_2_^+^, 628.3113; found, 628.3117.

## Data Availability

The data underlying this study are available in the published article and its Supporting Information. A data set collection of computational results is available in the ioChem-BD repository and can be accessed via 10.19061/iochem-bd-6-284.

## Supporting Information Available

Detailed synthetic procedures, crystallographic data, computational details and analyses, copies of ^1^H NMR, ^13^C NMR, and HRMS spectra, Cartesian coordinates of optimized geometries (PDF)
