# Supplementary material for: Revisiting Hafner’s Azapentalenes: The Chemistry of 1,3-Bis(dimethylamino)-2-azapentalene
Source: J Org Chem. 2024 Apr 17;89(9):5941–51. doi: 10.1021/acs.joc.3c02564 (PMC11077492; doi:10.1021/acs.joc.3c02564)
Supplement: Supplementary file 1 — jo3c02564_si_001.pdf [file jo3c02564_si_001.pdf]

Supporting Information

Revisiting Hafner's Azapentalenes: The Chemistry of 1,3-Bis(dimethylamino)-2-azapentalene

Enikő Meiszter,[a,b] Tamás Gazdag,[a,c] Péter J. Mayer,[a] Attila Kunfi,[a] Tamás Holczbauer,[d] Máté Sulyok-Eiler,[c,e] Gábor London*[a]

[a] MTA TTK Lendület Functional Organic Materials Research Group, Institute of Organic Chemistry, HUN-REN Research Centre for Natural Sciences, 1117 Budapest, Magyar tudósok krt. 2, Hungary

[b] Department of Organic Chemistry and Technology, Faculty of Chemical Technology and Biotechnology, Budapest University of Technology and Economics, Műegyetem rkp. 3., H-1111 Budapest, Hungary

[c] Hevesy György PhD School of Chemistry, Eötvös Loránd University, Pázmány Péter sétány 1/a, 1117 Budapest, Hungary

[d] Chemical Crystallography Research Laboratory and Stereochemistry Research Group, Institute for Organic Chemistry, HUN-REN Research Centre for Natural Sciences, 1117 Budapest, Magyar tudósok krt. 2, Hungary

[e] Laboratory of Structural Chemistry and Biology, Institute of Chemistry, ELTE Eötvös Loránd University, Pázmány Péter sétány 1/a, 1117 Budapest, Hungary

Email: london.gabor@ttk.hu

Table of contents:

S1 X-ray crystallography S3

S2 Computational characterization S7

S2.1 General methods S7

S2.2 Azapentalene **3** S8

S2.3 Comparison of azapentalenes with parent pentalene derivatives S14

S2.4 Protonation of azapentalene **3** S16

S2.5 NICS-XY scans of some derivatives of **3** S17

S2.6 FMO calculations of some derivatives of **3** S18

S2.7 Table of absolute energies for calculated structures S19

S3 NMR spectra S20

S4 HRMS spectra S40

S5 Cartesian coordinates S48

S6 References S55

S1 X-ray crystallography

Single crystals of compounds **3**, **12**, **14**, **17** and **18** were obtained from CH_2Cl_2 upon slow evaporation of the solvent. Intensity data of compounds **3**, **14** and **18** were collected on a Rigaku RAXIS-RAPID II diffractometer (using graphite monochromator; Mo-Kα radiation, λ = 0.71075Å and Cu-Kα, 1.54187Å) at normal and low temperature. Crystal Clear[1] (developed by Rigaku Company) software were used for data collection and refinement in all the case. Numerical absorption corrections[2] were applied to the data. Intensity data of compounds **12** and **17** were collected on a Rigaku Synergy-R diffractometer (using mirror monochromator; Cu-Kα, λ = 1.54187Å). Data reduction was carried out using CrysAlisPro program (v. 1.171.42.58a, Rigaku Oxford Diffraction, 2022) provided with the diffractometer. All the structures were solved by direct methods. Anisotropic full-matrix least-squares refinements were performed on F^2 for all non-hydrogen atoms. Hydrogen atoms bonded to C atoms were placed in calculated positions and refined in a riding-model approximation. The computer programs used for the structure solution, refinement and analysis of the structures were Shelx,[3,4] Wingx,[5] Platon,[6] and Olex2.[7] Program Mercury[8] was used for the graphical representation. Details of crystallographic data, data collection and refinement for crystals of **3**, **12**, **14**, **17** and **18** are collected in Table S1. Crystal structures are shown on Figure S1.

Deposition Numbers 2295069-2295072 and 2296808-2296809 contain the supplementary crystallographic data for this paper. These data are provided free of charge by the joint Cambridge Crystallographic Data Centre and Fachinformationszentrum Karlsruhe Access Structures service www.ccdc.cam.ac.uk/structures.

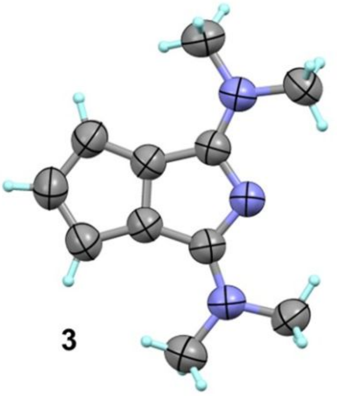

3

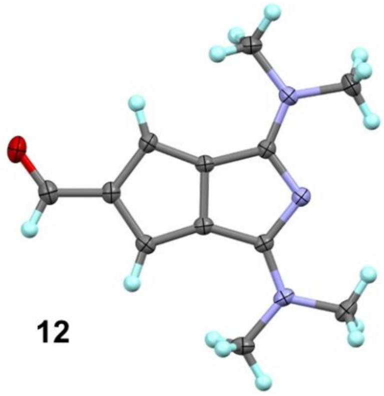

12

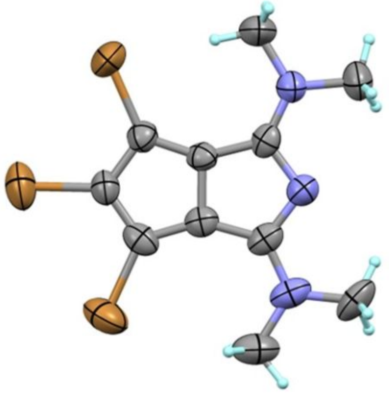

14

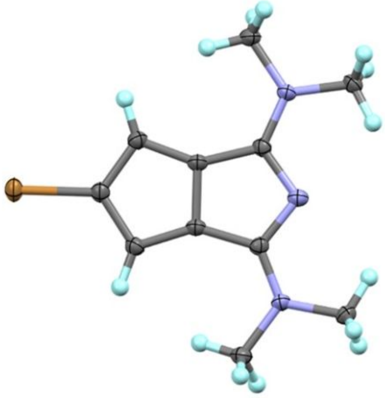

17

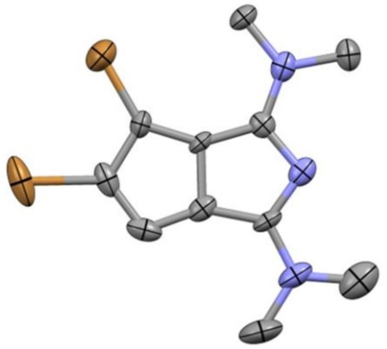

18

Figure S1. X-ray crystal structures of compounds **3**, **12**, **14**, **17** and **18** (ORTEP style representations are drawn at the 50% probability level, disordered H atoms are omitted for clarity).

Table S1. Crystal data and structure refinement of **3**, **12**, **14** and **18**.

Number	**3**	**12**
CCDC	2295071	2296808
Empirical formula	$C_{11}H_{15}N_3$	$C_{12}H_{15}N_3O$
Formula weight	189.26	217.27
Temperature	294(2)	100.0(1)
Radiation and wavelength	Cu-Kα,	Cu-Kα,
	λ =1.54187Å	λ =1.54187Å
Crystal system	monoclinic	orthorhombic
Space group	*I*2/a	*P n m a*
Unit cell dimensions	*a* =11.2382(13)Å	*a* = 9.5149(2) Å
	b =8.4235(10)Å	*b* = 6.7606(2) Å
	c =11.282(2)Å	*c* = 17.3333(3) Å
	α =90°	α =90°
	β =105.651(11)°	β =90°
	γ =90°	γ =90°
Volume	1028.4(3)$Å^3$	1114.99(4) $Å^3$
Z, Z'	4, 0.5	4, 0.5
Density (calculated)	1.222 Mg/m^3	1.294 Mg/m^3
Absorption coefficient, μ	0.590 mm^{-1}	0.686 mm^{-1}
F(000)	408	464
Crystal colour, description	red, chunk	red, block
Crystal size	0.50 x 0.50 x 0.50mm	0.24 x 0.07 x 0.05 mm
Absorption correction	numerical	numerical
Max. and min. transmission	0.149518 and 0.317123	0.829 and 1.000
θ–range for data collection	4.929 ≤ θ ≤ 68.189°	5.103 ≤ θ ≤ 75.298 °
Index ranges	-13 ≤ *h* ≤13;	-11 ≤ *h* ≤11;
	-10≤ *k* ≤10;	-8≤ *k* ≤6;
	-13 ≤ *l* ≤13	-21 ≤ *l* ≤21
Reflections collected	13343	12047
Completeness to 2θ	0.981	0.986
Independent reflections	927 [*R*(int) =0.0538]	1235 [*R*(int) = 0.0467]
Reflections *I*>2σ(*I*)	879	1146
Data / restraints / parameters	927 /0 /68	1235 /0 /101
Goodness-of-fit on F^2	1.065	1.078
Final *R* indices [*I*>2σ(*I*)]	R_1 =0.0666,	R_1 =0.0416,
	wR^2 =0.1707	wR^2 =0.1034

R indices (all data)	R_1 =0.0701,	R_1 =0.0446,
	wR^2 =0.1830	wR^2 =0.1060
Max. and mean shift/esd	0.001;0.000	0.000;0.000
Largest diff. peak and hole	0.33;-0.16 e.Å^{-3}	0.192;-0.389 e.Å^{-3}

Table S1 (cont.). Crystal data and structure refinement of **3**, **12**, **14** and **18**.

Number	**17**	**18**	**14**
CCDC	2296808	2295072	2295070
Empirical formula	$C_{11}H_{14}BrN_3$	$C_{11}H_{13}Br_2N_3$	$C_{11}H_{12}Br_3N_3$
Formula weight	268.16	347.06	425.97
Temperature	100.0(1)	110.68(12)	294(2)
Radiation and wavelength	Cu-Kα, λ =1.54184Å	Cu-Kα, λ =1.54184Å	Mo-Kα, λ =0.71075Å
Crystal system	monoclinic	monoclinic	monoclinic
Space group	*I 2/a*	*C* 2/*m*	*P* 2_1/c
Unit cell dimensions	*a* =9.92737(17)	*a* =17.3221(4)Å	*a* =7.2932(6)Å
	b =10.40876(18)	*b* =6.85739(17)Å	*b* =16.8495(11)Å
	c =10.86135(19)	*c* =10.8362(3)Å	*c* =11.4284(8)Å
	α =90°	α =90°	α =90°
	β =98.5424(16)°	β =93.574(2)°	β =101.662(7)°
	γ =90°	γ =90°	γ =90°
Volume	1109.87(3)	1284.67(5)$Å^3$	1375.41(18)$Å^3$
Z, Z'	4, 0.5	4, 1	4, 1
Density (calculated)	1.605 1.794 Mg/m^3	1.794 Mg/m^3	2.057 Mg/m^3
Absorption coefficient, μ	4.763	7.820 mm^{-1}	8.781 mm^{-1}
F(000)	544	680	816
Crystal colour, description	red, irregular	colourless, prism	red, block
Crystal size	0.11 x 0.09 x 0.07 mm	0.5 x 0.05 x 0.05 mm	0.40 x 0.15 x 0.10 mm
Absorption correction	numerical	gaussian	numerical
Max. and min. transmission	0.744 and 0.884	0.049 and 0.878	0.278798 and 0.640550
θ–range for data collection	5.920≤ θ ≤ 75.569	4.088 ≤ θ ≤ 68.241°	3.026 ≤ θ ≤ 25.317°
Index ranges	-11 ≤ *h* ≤12;	-20 ≤ *h* ≤20;	-8 ≤ *h* ≤8;
	-12≤ *k* ≤12;	-8≤ *k* ≤8;	-20≤ *k* ≤20;
	-13 ≤ *l* ≤13	-13 ≤ *l* ≤11	-13 ≤ *l* ≤13
Reflections collected	13427	11159	40929
Completeness to 2θ	0.992	0.979	0.999
Independent reflections	1146 [*R*(int) =0.0463]	1260 [*R*(int) =0.0466]	2500 [*R*(int) =0.1257]
Reflections *I*>2σ(*I*)	1117	1229	1683
Data / restraints / parameters	1146 / 0 / 72	1260 /0 /106	2500 /0 /171
Goodness-of-fit on *F*2	1.090	1.143	1.034
Final *R* indices [*I*>2σ(*I*)]	R_1 =0.0317, wR^2 =0.0907	R_1 =0.0987, wR^2 =0.2294	R_1 =0.0544, wR^2 =0.0992

R indices (all data)	R_1 =0.0320, wR^2 =0.0909	R_1 =0.0999, wR^2 =0.2299	R_1 =0.0945, wR^2 =0.1108
Max. and mean shift/esd	0.000;0.000	0.002;0.001	0.008;0.000
Largest diff. peak and hole	1.020;-0.873 e.$Å^{-3}$	1.53;-1.45 e.$Å^{-3}$	0.73;-0.81 e.$Å^{-3}$

S2 Computational characterization

S2.1 General methods

Calculations were performed with the Gaussian 09 rev E.01[9] package. In a recent report by Haley and Wu, it was shown that calculations with the B3LYP hybrid functional lead to systematic errors, which can be avoided by using the M11 functional.[10] Therefore, we tested several DFT functionals (B3LYP,[11] CAM-B3LYP,[12] wB97XD,[13] M06-2X,[14] M11,[15] and M11-L[16]) for our systems. For the basis set, we used 6-311+G(d,p).[17] Analytical hessians were computed to confirm that the structures are minima.

To choose the best-fitting DFT method, we compared the calculated UV-Vis spectra and the ^{1}H NMR shifts of **3** using the above mentioned functionals with the experimental data. We found that B3LYP is gives the best approximation of the UV-Vis data, while M11L gives the best fitting for the chemical shifts. This shows that there is no ultimate method for the computational description of these systems. For further calculations, we only used the B3LYP functional.

Several indices are available for the computational evaluation of (anti)aromaticity, including those based on magnetic,[18] geometric,[19] electronic,[20] reactivity,[21,22] and orbital localization properties.[23] Since the magnetic indices are arguably the most popular descriptors we used those for our systems. As magnetic indicators, the anisotropy of the induced current density (ACID) plots[24,25] and vertical and horizontal NICS scans[26,27] were used. ACID plots give a qualitative picture of the ring-currents so that clockwise and anticlockwise ring-currents indicate aromatic and antiaromatic character, respectively. NICS scans, which consider only π-contributions by employing the σ-model, provide quantitative information about the diatropicity (negative values) and paratropicity (positive values) of the different circuits. The NICS scans were calculated with the Aroma package.[28] For azapentalene **3** HOMA values were also calculated to characterize the (anti)aromatic character of its rings.[29] Aromatic rings are characterized by HOMA values in the range 0.5−1.0, while negative HOMA values are indicative of antiaromaticity.

The input and output files for all calculations are available in the ioChem-BD - The Computational Chemistry Results Repository.[30]

S2.2 Azapentalene 3

The structure of azapentalene **3** was calculated with 6 different DFT methods and these structures were used to calculate UV-Vis spectra with modelled DCM at the TD-DFT//DFT and TD-B3LYP//DFT levels (Figure S2). TD-B3LYP is known to have a good estimation for the HOMO-LUMO transitions.[31] The qualitative picture shows that TD-B3LYP//B3LYP provides the best fit . For a quantitative analysis the first 3 absorption peaks are compared in Table S2, which also shows that TD-B3LYP//B3LYP fits best the measured results.

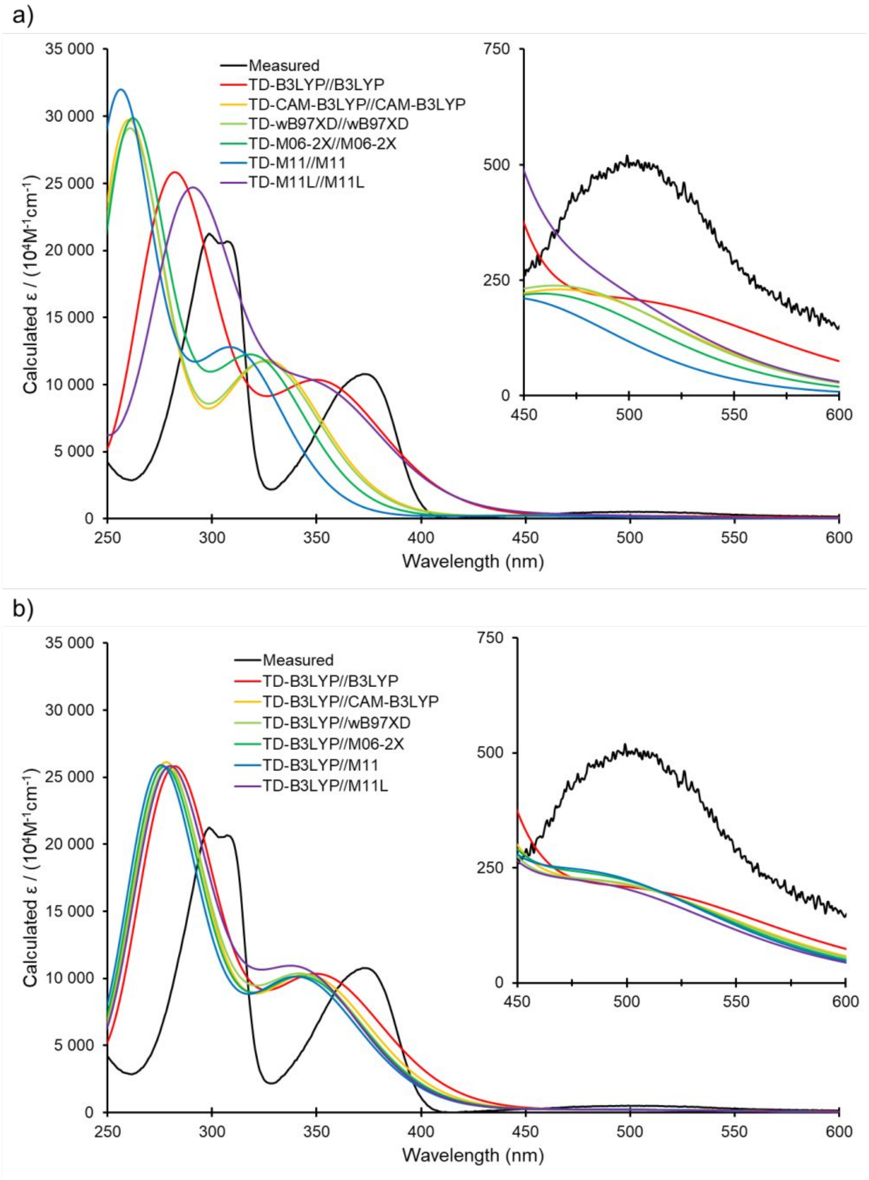

Figure S2. Calculated UV-Vis spectra of **3** at the a) TD-DFT/6-311+G(d,p)/IEFPCM(DCM) //DFT/6-311+G(d,p) level and at the b) TD-B3LYP/6-311+G(d,p)/IEFPCM(DCM)//DFT/6-311+G(d,p) level.

Table S2. First three absorbance peaks of **3** measured in DCM and calculated at the TD-DFT/6-311+G(d,p)/IEFPCM(DCM) //DFT/6-311+G(d,p) level.

Entry	DFT	TD-DFT	λ_{abs1} (nm)	λ_{abs2} (nm)	λ_{abs3} (nm)
1	*Measured*		500	375	300
2	B3LYP		504	352	283
3	CAM-B3LYP		470	327	261
4	wB97XD		468	326	261
5	M06-2X		461	320	262
6	M11		445	311	256
7	M11L		468	353	292
8	CAM-B3LYP	B3LYP	492	347	279
9	wB97XD	B3LYP	490	345	279
10	M06-2X	B3LYP	485	344	278
11	M11	B3LYP	482	342	276
12	M11L	B3LYP	484	343	281

The NMR calculations were performed in a similar manner to that used in the paper by Wu and Haley.[10] The chemical shifts were calculated at B97-2/6-311+G(d,p) level. The proton chemical shifts (δ_H) were calculated with the following equation:

$$\delta_H = (\sigma_{Benzene} - \sigma_H) + \delta_{Benzene}$$

where δ_H is the calculated chemical shift, $\delta_{Benzene}$ is the chemical shift of benzene's protons, σ_H is the calculated shielding for the proton, $\sigma_{Benzene}$ is the calculated shielding for benzene's protons. The chemical shift and shielding for benzene were taken from the above mentioned reference.[10] Based on the calculated chemical shifts M11L is somewhat better than the other functionals. However, the UV-vis spectra calculated with B3LYP fits better than those with M11L, which is why we used B3LYP for further calculations.

Table S3. Comparison of calculated ^{1}H NMR shifts of **3** with the measured data.

Entry	Method	Chemical Shifts H	H
1	*Measured*	6.19	6.05
2	B3LYP	5.90	5.63
3	CAM-B3LYP	5.84	5.56
4	wB97XD	5.82	5.66
5	M062X	5.82	5.69
6	M11	5.84	5.76
7	M11L	5.94	5.66

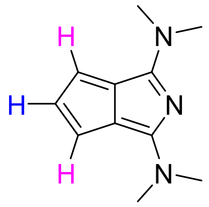

HOMO-LUMO transition energies for **3** were also calculated with modelled MeCN solvent at the TD-B3LYP/6-311+G(d,p) level of theory. The frontier molecular orbitals are shown in Figure S3, further information about the excitation can be found in Table S4, and calculated UV-Vis spectra compared to the measured ones are shown in Figure S4 with the specific transitions indicated.

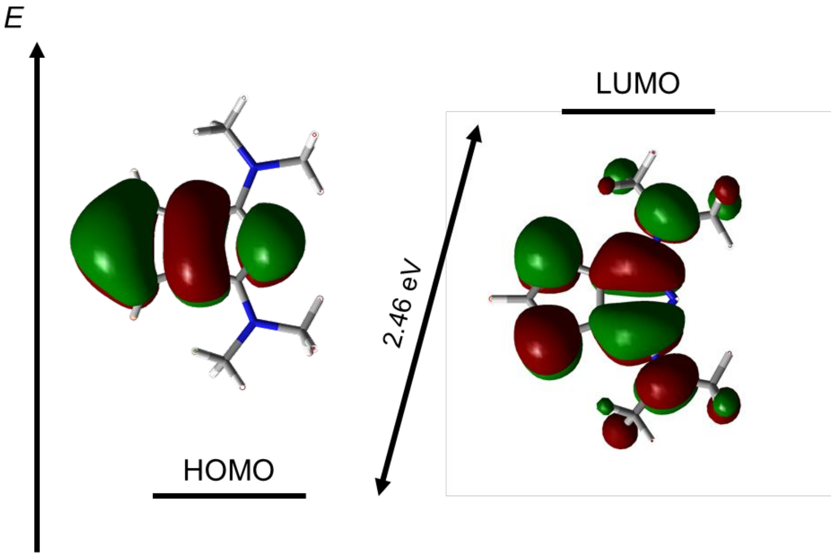

Figure S3. Calculated frontier orbitals for **3** (at the 0.02 a.u. isosurface) and calculated HOMO-LUMO energy difference in eV at the B3LYP/6-311+G(d,p)/IEFPCM(MeCN)//B3LYP /6-311+G(d,p) level.

Table S4. Calculated excitations, contributions, energies, and oscillator strengths (*f*) for molecule **3** with TDDFT at the B3LYP/6-311+G(d,p)/IEFPCM(MeCN)// B3LYP /6-311+G(d,p) and at B3LYP /6-311+G(d,p)/IEFPCM(DCM)// B3LYP /6-311+G(d,p) level of theory.

MeCN solvent model					
Excitation	Contributing transitions	Coefficient	Excitation energy		Oscillator strength
			eV	nm	
1	51 (HOMO) → 52 (LUMO)	0.702	2.49	497	0.0045
2	50 → 52	0.701	3.55	350	0.2391
3	48 → 52	-0.185	4.38	283	0.5651
	49 → 52	0.676			
4	48 → 52	0.681	4.57	271	0.0622
	49 → 52	0.183			
5	51 → 53	0.682	4.67	265	0.0047
	51 → 55	0.122			
	51 → 57	-0.112			

DCM solvent model					
Excitation	Contributing transitions	Coefficient	Excitation energy		Oscillator strength
			eV	nm	
1	51 (HOMO) → 52 (LUMO)	0.703	2.46	504	0.0049
2	50 → 52	0.702	3.52	352	0.2491
3	48 → 52	-0.192	4.38	283	0.5756
	49 → 52	0.674			
4	48 → 52	0.679	4.56	272	0.0678
	49 → 52	0.189			
5	51 → 53	0.682	4.63	268	0.0043
	51 → 55	0.122			

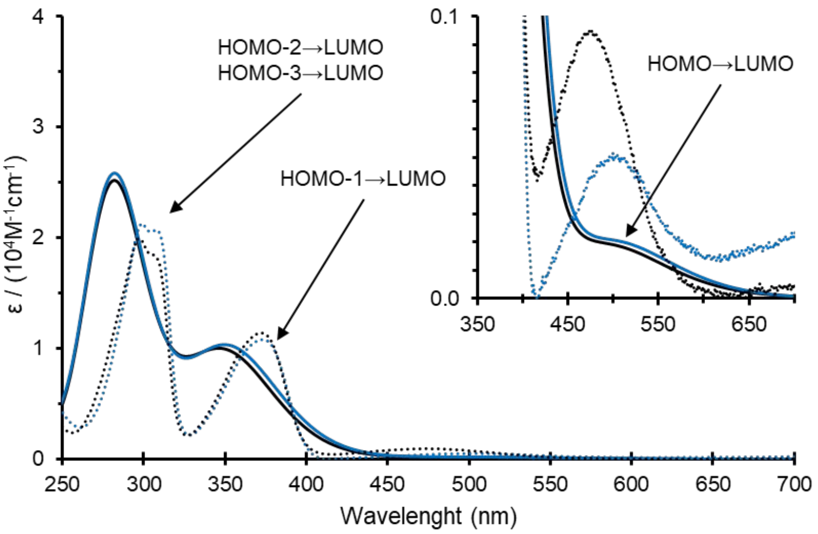

Figure S4. Calculated UV-Vis spectra for **3** at B3LYP/6-311+G(d,p)/IEFPCM(MeCN)//B3LYP /6-311+G(d,p) (black line), and at the B3LYP/6-311+G(d,p)/IEFPCM(DCM)//B3LYP /6-311+G(d,p) (blue line) level of theory with the corresponding transition pointed out. Measured UV-Vis spectra for **3** in acetonitrile (dotted black line) and in dichloromethane (dotted blue line).

The aromatic character of azapentalene **3** was evaluated by NICS, ACID, HOMA indices, and bond length alternation. All of these methods show weak aromatic character in the carbocyclic '*a*' ring of **3** and weak antiaromatic character in the heterocyclic '*b*' ring of **3**.

$NICS(1)_{\pi ZZ}$ values above the center of the rings were calculated and show weak aromatic character in ring '*a*' (small negative values) and antiaromatic character in ring '*b*' ($NICS(1)_{\pi ZZ}$=24.5) (Figure S5 a). Calculated ACID plots show a small aromatic (clockwise) induced ring circuit in ring '*a*' and a stronger antiaromatic (anti-clockwise) circuit in ring '*b*' (Figure S5 b,c).

The bond length alternation in **3** for ring '*a*' is small while for ring '*b*' is larger, which is also reflected in the calculated HOMA values (Figure S6).

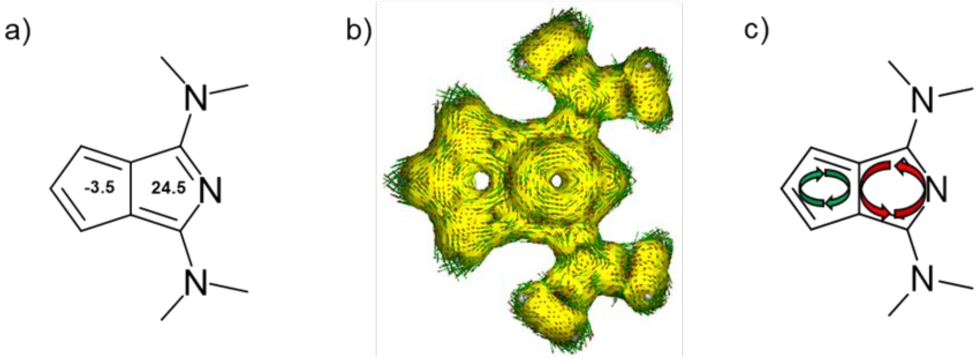

Figure S5. Magnetic aromatic indices calculated for **3** at the B3LYP /6-311+G(d,p) level. a) NICS(1)$_{\pi zz}$ values above the ring centers; b) ACID plot calculated with isosurface value 0.025; c) Simplified interpretation of the ACID plot (green circle represents aromatic, red circle represents antiaromatic induced ring current).

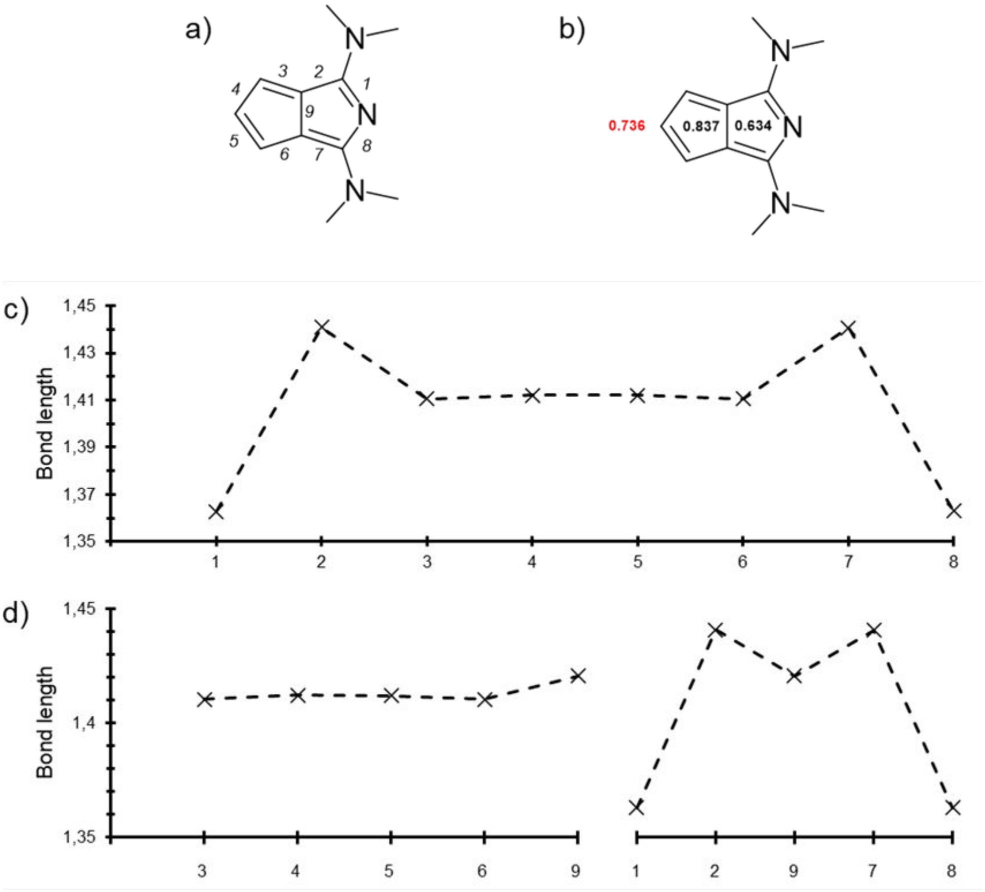

Figure S6. HOMA values and bond length alteration for **3** based on the structure calculated at the B3LYP /6-311+G(d,p) level. a) Numbering of the bonds; b) HOMA values for the rings (black) and for the perimeter (red); c) Bond length alteration through the perimeter; d) Bond length alteration in ring '*a*' (left) and ring '*b*' (right).

S2.3 Comparison of azapentalenes with parent pentalene derivatives

For comparison in aromatic character pentalene (**1**), unsubstituted 2-azapentalene (**2**), and bis(dimethylamino) substituted pentalene (**S1**) (derivatives of which was reported in a recent study[32]) was studied with the same indices as **3**.

The ACID plots of the molecules show strong global antiaromatic induced ring circuits for both **1** and **2** while showing similar local antiaromatic circuits for **3** and **S1** (Figure S7).

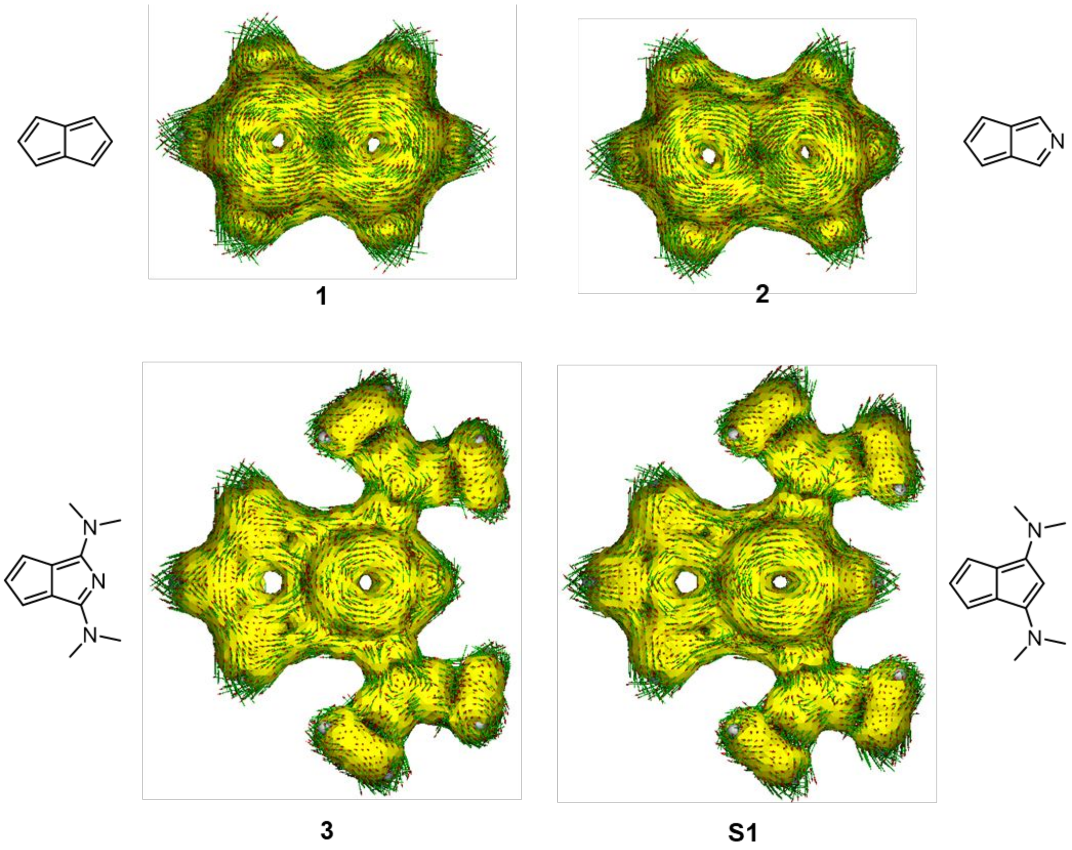

Figure S7. ACID plots of **1**, **2**, **3** and **S1** calculated at the B3LYP /6-311+G(d,p) level of theory with isosurface value 0.025.

The HOMA values (Table S5) show global antiaromatic character for **1**, non-aromatic character for **2**, and weak aromatic character for **3** and **S1**. While showing that incorporation of a N has the largest effect on the ring into which it is incorporated. However, the donor substituents affect the other, carbocyclic ring more (‘*a*’).

The above-mentioned substituent effect can also be seen in the NICS(1)$_{\pi ZZ}$ values (Table S5). However, the effect of N incorporation is opposite compared to what is seen in HOMA. However, the general tendency of pentalene derivatives having lower antiaromaticity can be seen on the NICS-*XY* scans (Figure S8).

Table S5. Calculated HOMA and NICS(1)$_{\pi ZZ}$ values for **1**, **2**, **3** and **S1** at the B3LYP/6-311+G(d,p) level of theory.

Entry	Molecule	X=	R=	HOMA			NICS(1)$_{\pi ZZ}$	
				ring *a*	ring *b*	perimeter	ring *a*	ring *b*
1	**1**	CH	H	-0.322	-0.322	-0.346	56.5	56.5
2	**2**	N	H	-0.008	0.213	0.074	50.5	54.4
3	**S1**	CH	NMe_2	0.833	0.481	0.635	10.5	38.0
4	**3**	N	NMe_2	0.837	0.634	0.736	-3.5	24.5

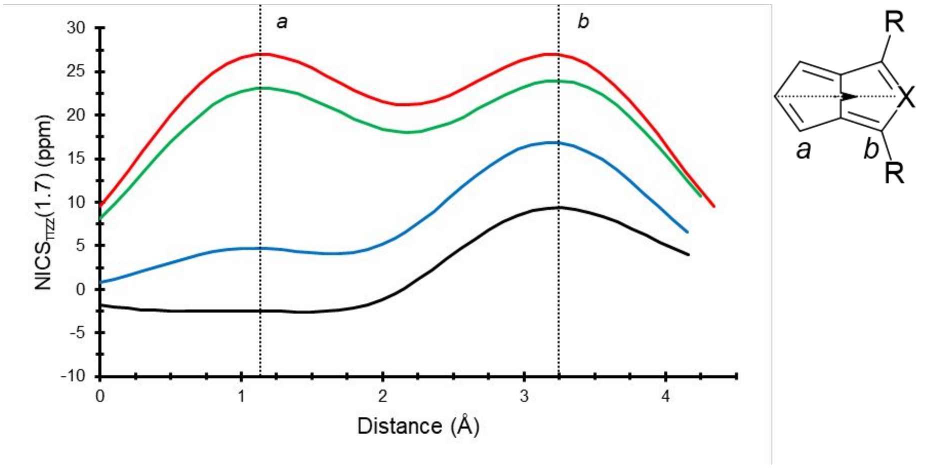

Figure S8. NICS-*XY* scans for **1** (red line), **2** (green line), **3** (black line), and **S1** (blue line) calculated at the B3LYP /6-311+G(d,p) level of theory.

Compound **3** was compared to its parent pentalene derivatives, and two effects could be concluded from these comparisons. The (1) incorporation of nitrogen to the pentalene ring system and the (2) electron donating effect of the dimethylamino groups both lower the antiaromatic character of such derivatives. These two effects add up in the case of **3**.

S2.4 Protonation of azapentalene 3

To evaluate the effect of protonation of azapentalene derivatives we calculated both potential protonated forms (Figure S9), compared their energies, and also look at how the protonation affects the aromatic character of the compounds.

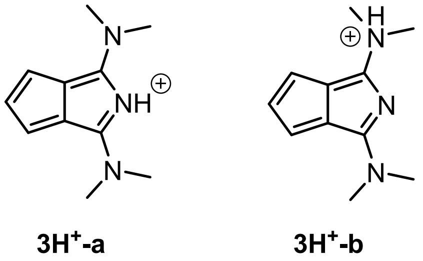

Figure S9. The possible protonated forms of **3**.

Both **3H⁺-a** and **3H⁺-b** were calculated at the B3LYP/6-311+G(d,p) level of theory in gas phase, and with modeled water solvent (SMD[33]) the energy difference was found to be in favor of the protonation on the ring nitrogen by ~26.7 kcal/mol and ~25.5 kcal/mol, respectively for the gas and solvent models.

The effect of both protonation modes was also studied from an aromaticity viewpoint. It was found that if the protonation occurs on the ring nitrogen the aromatic character of the heterocyclic ring '*b*' increases, but in case it is on the dimethylamino group it leads to an overall antiaromaticity increase (Figure S10). This could be explained as if the protonation would occur on the dimethylamino group its π donating character would be eliminated, hence its stabilizing effect would diminish. These results are in good agreement with the energetic considerations, and both support the experimental findings.

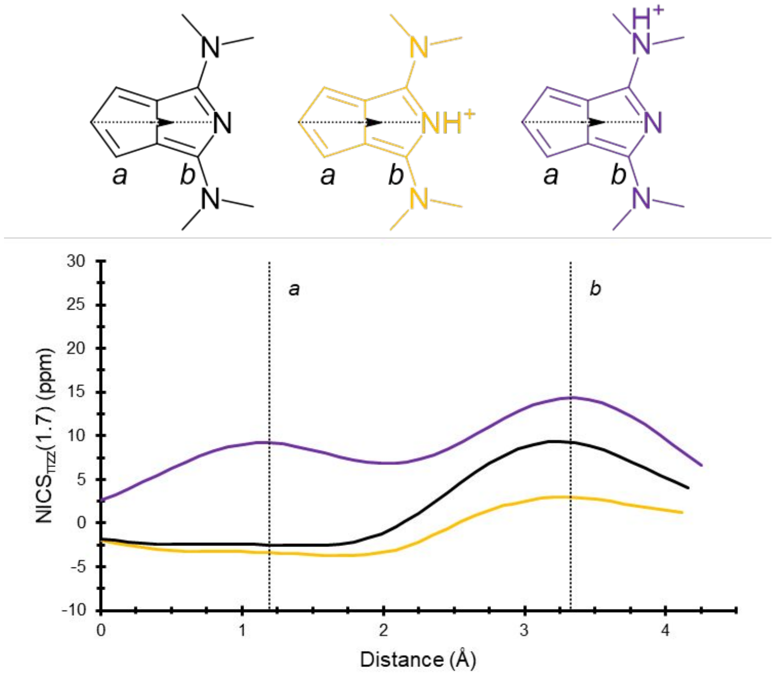

Figure S10. NICS-XY scans of **3** (black), **3H$^+$-a** (orange), and **3H$^+$-b** (purple) calculated at the B3LYP/6-311+G(d,p) level of theory.

S2.5 NICS-XY scans of some derivatives of 3

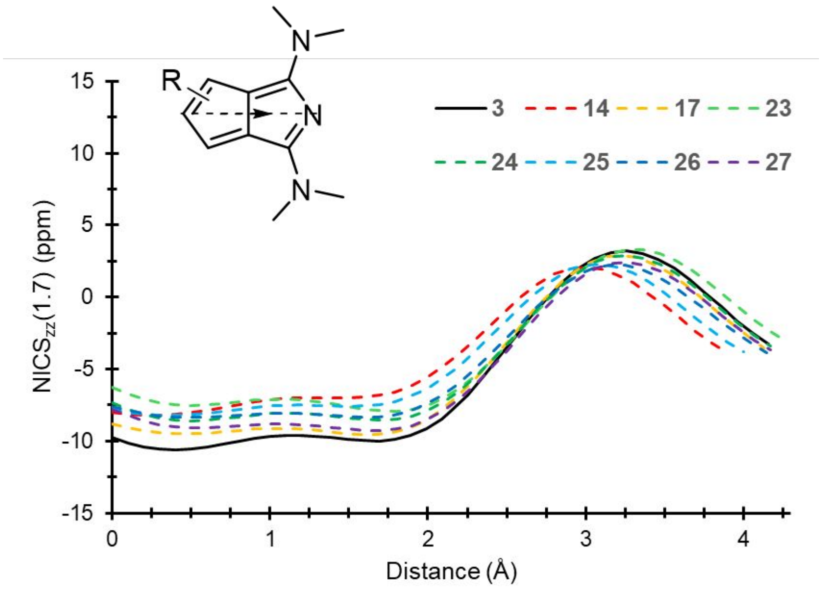

Figure S11. NICS-*XY* scans of derivatives of **3**.

The NICS-*XY* scans were studied for the different substituted derivatives of **3**. No substantial changes in the (anti)aromatic nature of the rings were obtained.

S2.6 FMO calculations of some derivatives of 3

Table S6. Calculated (B3LYP/6-311+G(d,p)) orbital energies and energy gaps for some derivatives of compound **3**.

**Compound**	E_{HOMO} / eV	E_{LUMO} / eV	ΔE_{gap} / eV
**3**	-4.88499	-1.57091	3.314076
**23**	-4.87247	-1.80466	3.067813
**24**	-4.72499	-1.74017	2.984818
**25**	-5.34677	-2.29038	3.056384
**26**	-5.2986	-2.30916	2.989444
**27**	-4.88227	-1.8648	3.017472

S2.7 Table of absolute energies for calculated structures

Table S7. Table of calculated absolute electronic energies for molecule **1-3** and **S1**, **$3H^+$-a** and **$3H^+$-b** with different methods. The structures can be found in the ioChem-BD repository.[30]

Entry	Molecule	Method	Basis set	Electronic energies (Hartee)	Number of imaginary frequencies
1	**1**	B3LYP	6-311+G(d,p)	-308.44761	0
2	**2**	B3LYP	6-311+G(d,p)	-324.49235	0
3	**3**	B3LYP	6-311+G(d,p)	-592.56830	0
4	**3**	CAM-B3LYP	6-311+G(d,p)	-592.24059	0
5	**3**	wB97XD	6-311+G(d,p)	-592.36672	0
6	**3**	M06-2X	6-311+G(d,p)	-592.30884	0
7	**3**	M11	6-311+G(d,p)	-592.20943	0
8	**3**	M11L	6-311+G(d,p)	-592.43676	0
9	**S1**	B3LYP	6-311+G(d,p)	-576.49448	0
10	**$3H^+$-a**	B3LYP	6-311+G(d,p)	-592.60212	0
11	**$3H^+$-b**	B3LYP	6-311+G(d,p)	-592.55925	0
12	**14**	B3LYP	6-311+G(d,p)	-8313.18498	0
13	**17**	B3LYP	6-311+G(d,p)	-3166.11351	0
14	**23**	B3LYP	6-311+G(d,p)	-1285.89219	0
15	**24**	B3LYP	6-311+G(d,p)	-823.68306	0
16	**25**	B3LYP	6-311+G(d,p)	-6460.49295	0
17	**26**	B3LYP	6-311+G(d,p)	-4607.80165	0
18	**27**	B3LYP	6-311+G(d,p)	-1313.41823	0

S3 NMR spectra

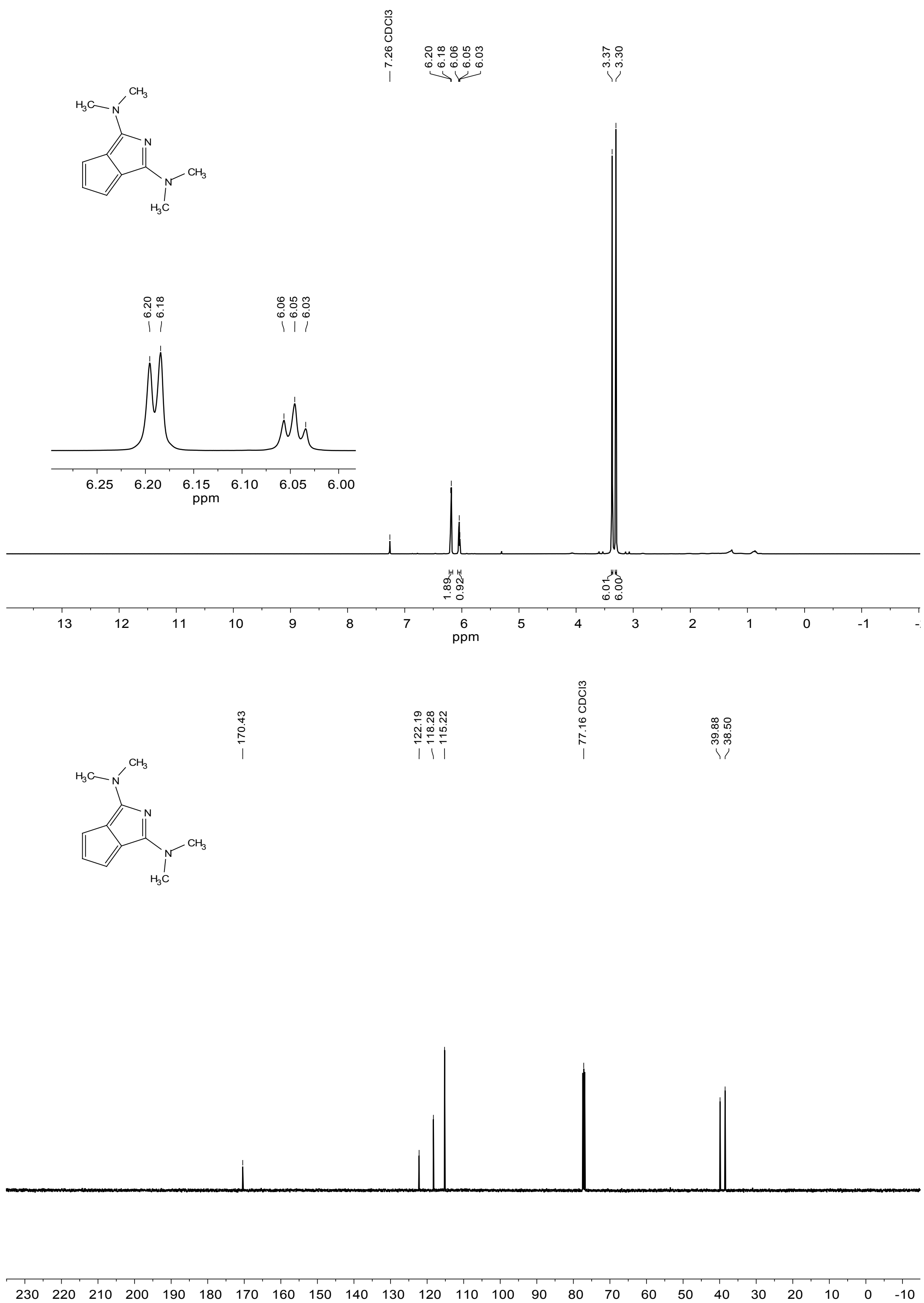

Figure S12. ^{1}H NMR (top, $CDCl_3$, 500 MHz) and $^{13}C\{^{1}H\}$ NMR (bottom, $CDCl_3$, 126 MHz) spectra of **3**.

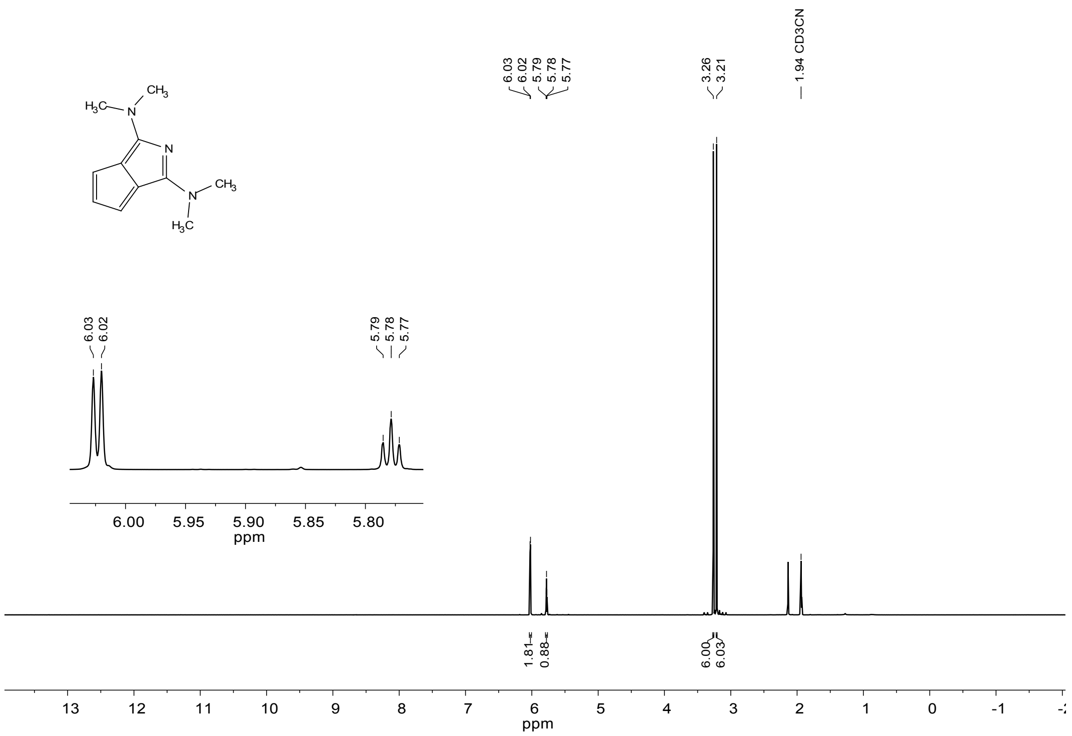

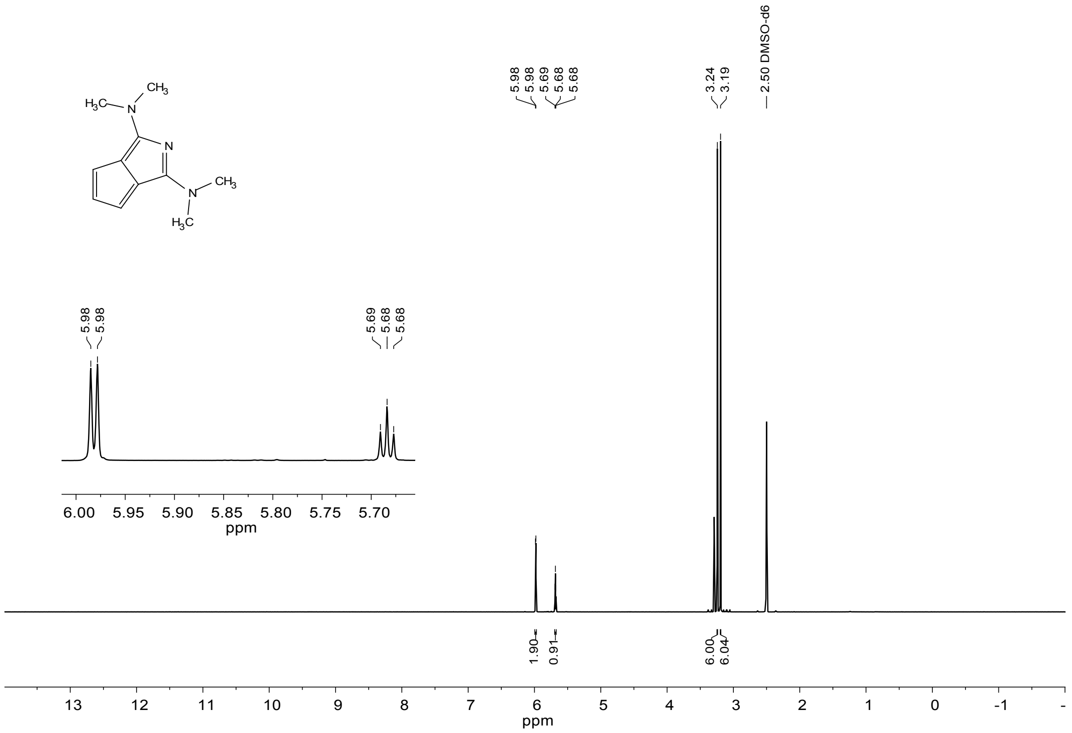

Figure S13. ^{1}H NMR (top, CD_3CN, 500 MHz) and ^{1}H NMR (bottom, DMSO-d_6, 500 MHz) spectra of **3**.

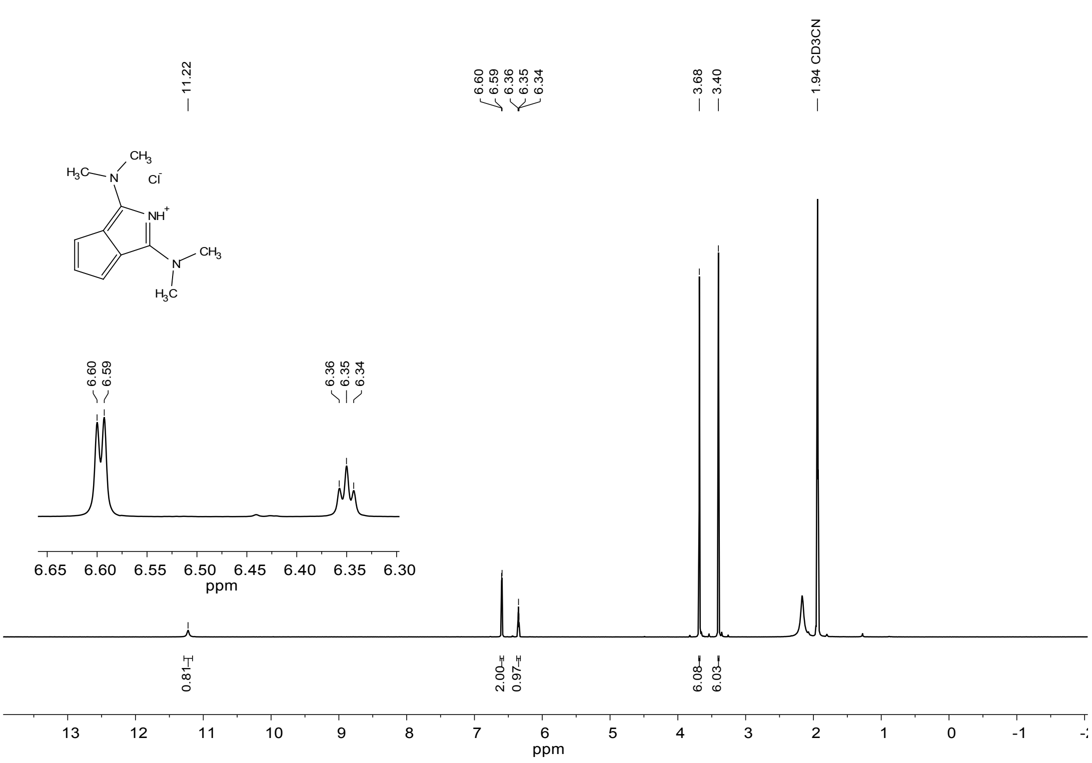

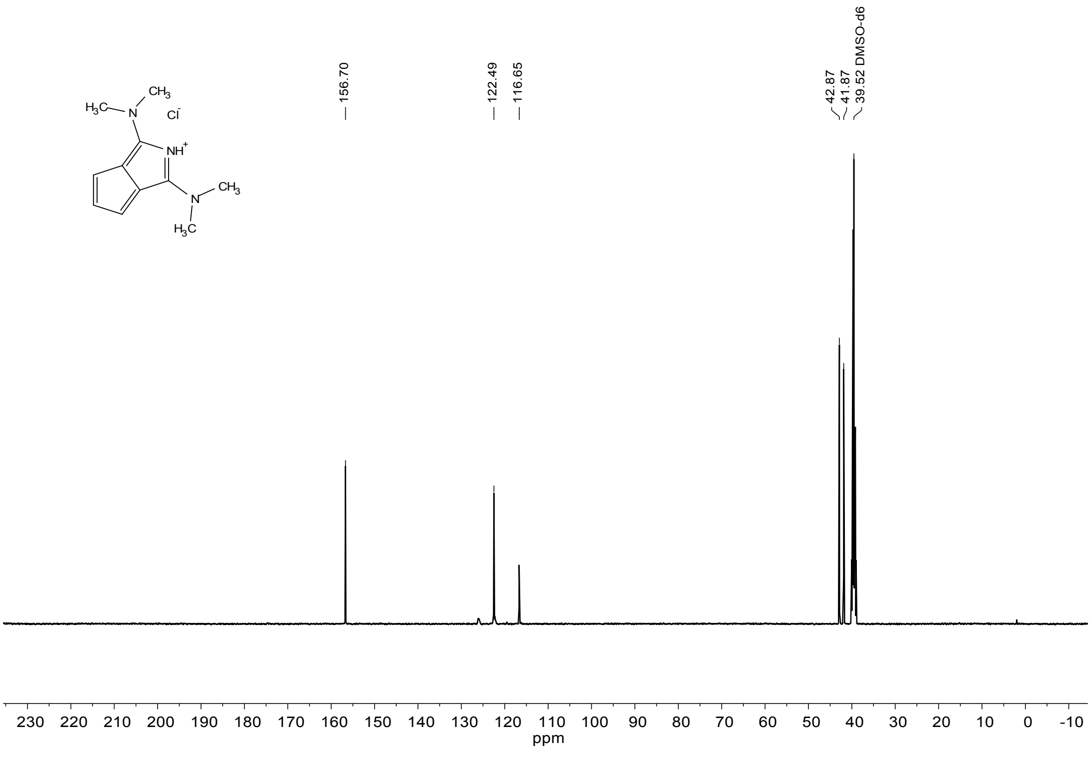

Figure S14. ^{1}H NMR (top, CD_3CN, 500 MHz) and $^{13}C\{^1H\}$ NMR (bottom, DMSO-d_6: D_2O = 4 : 1, 126 MHz) spectra of **3·HCl**.

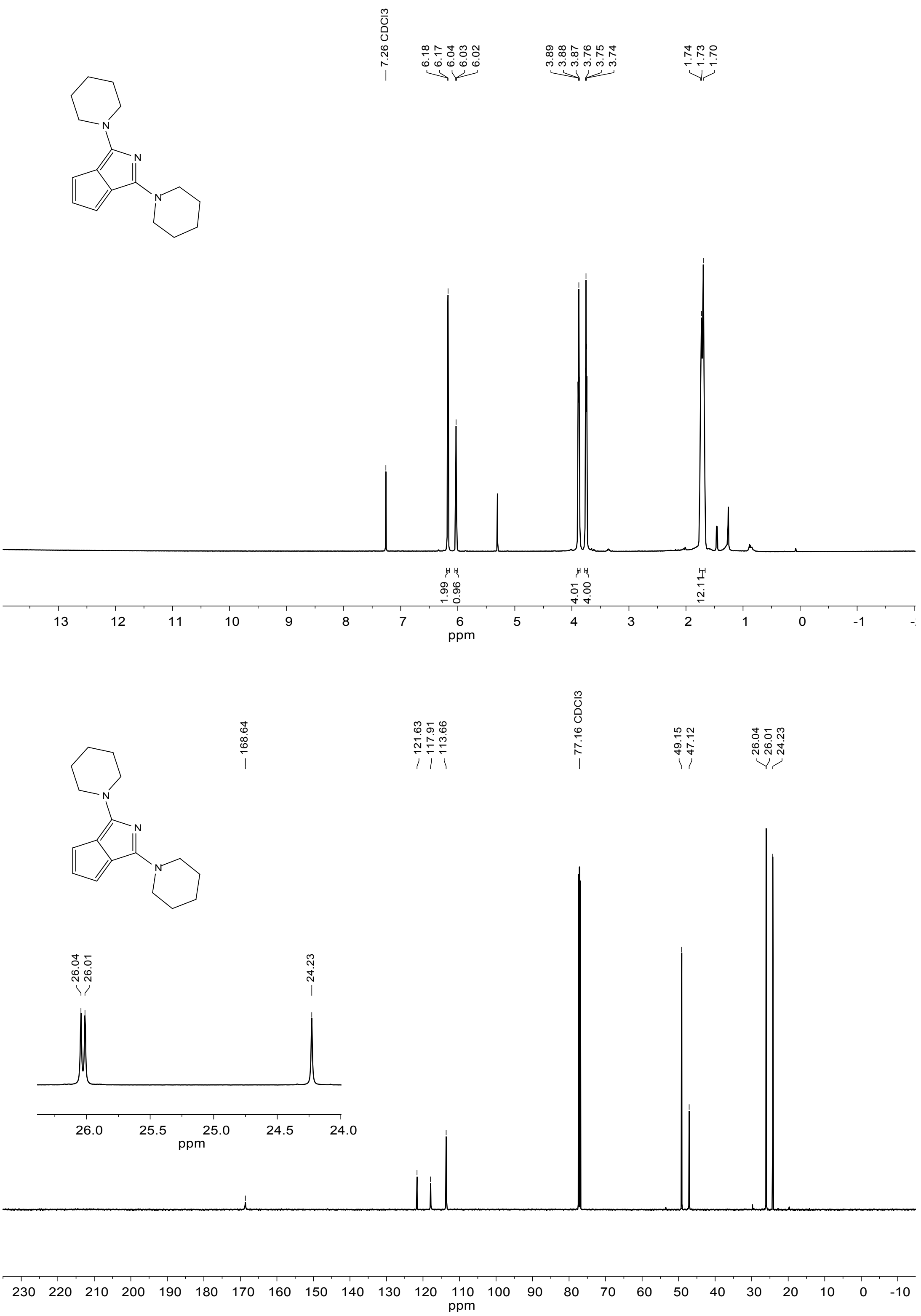

Figure S15. ^{1}H NMR (top, $CDCl_3$, 500 MHz) and $^{13}C\{^{1}H\}$ NMR (bottom, $CDCl_3$, 126 MHz) spectra of **8**.

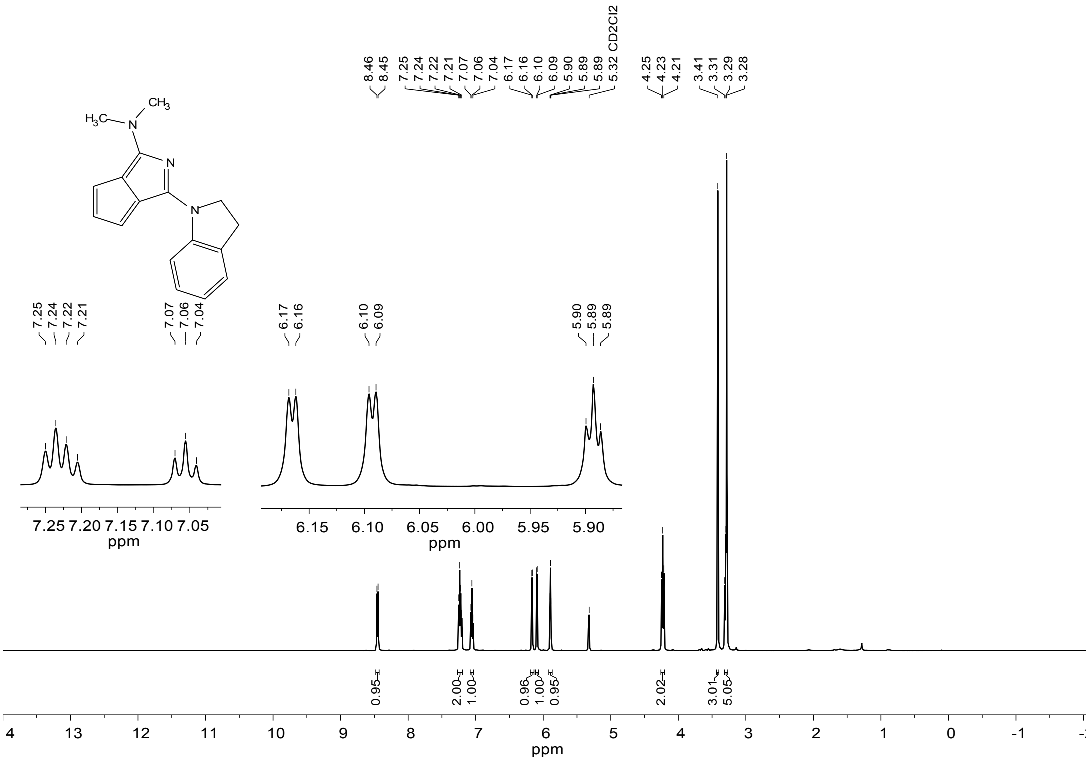

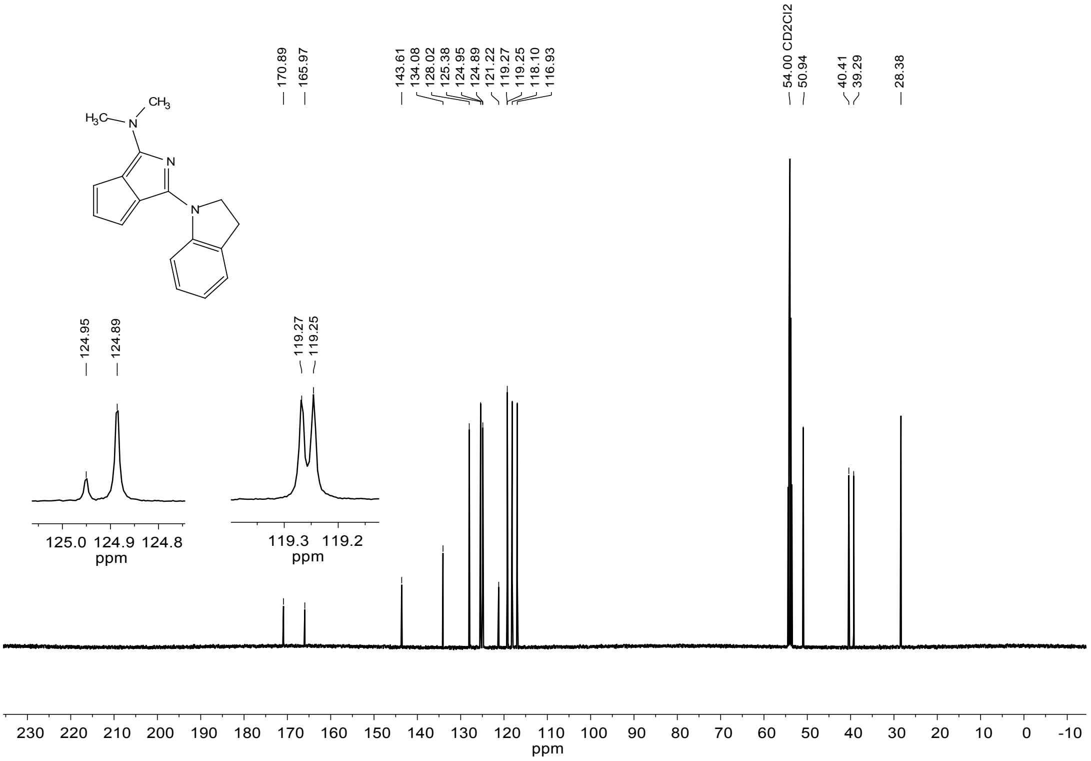

Figure S16. ^{1}H NMR (top, CD_2Cl_2, 500 MHz) and $^{13}C\{^{1}H\}$ NMR (bottom, CD_2Cl_2, 126 MHz) spectra of **11**.

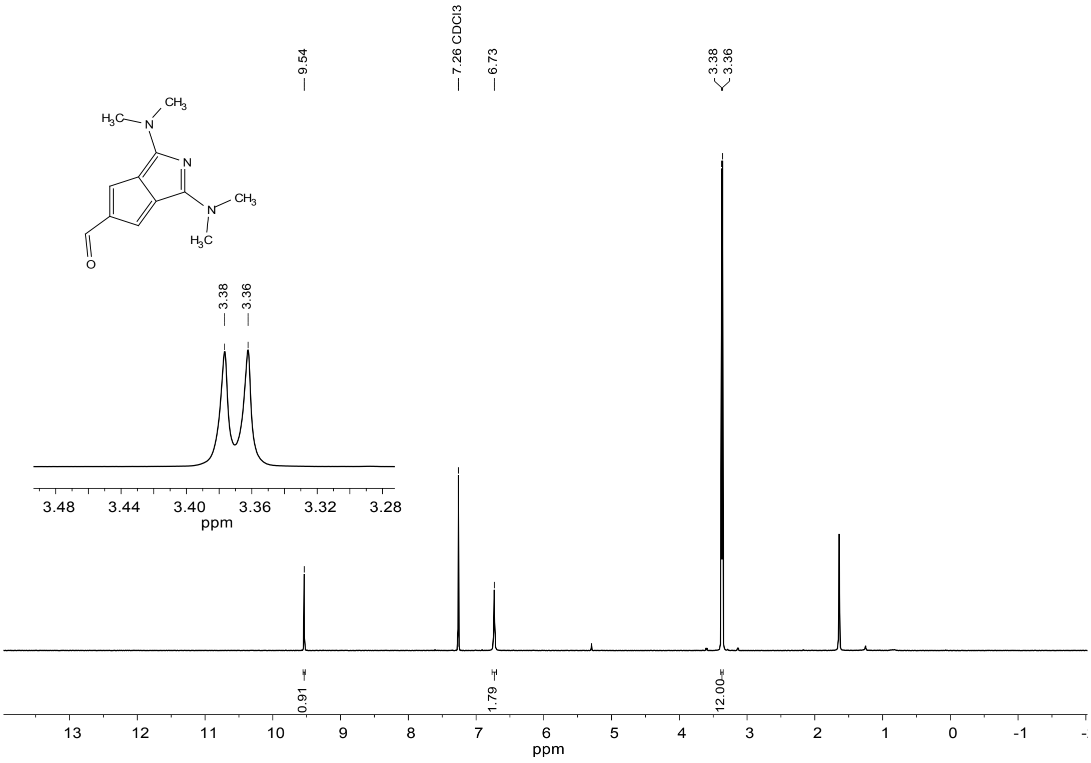

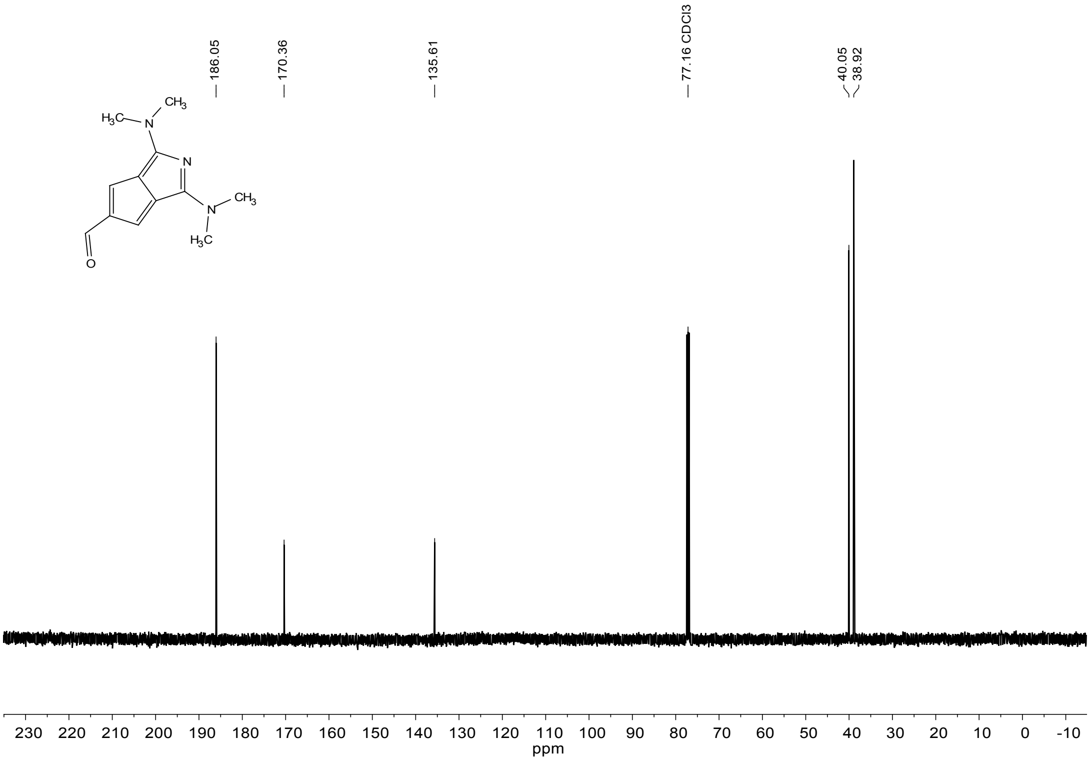

Figure S17. ^{1}H NMR (top, $CDCl_3$, 500 MHz) and $^{13}C\{^{1}H\}$ NMR (bottom, $CDCl_3$, 126 MHz) spectra of **12**.

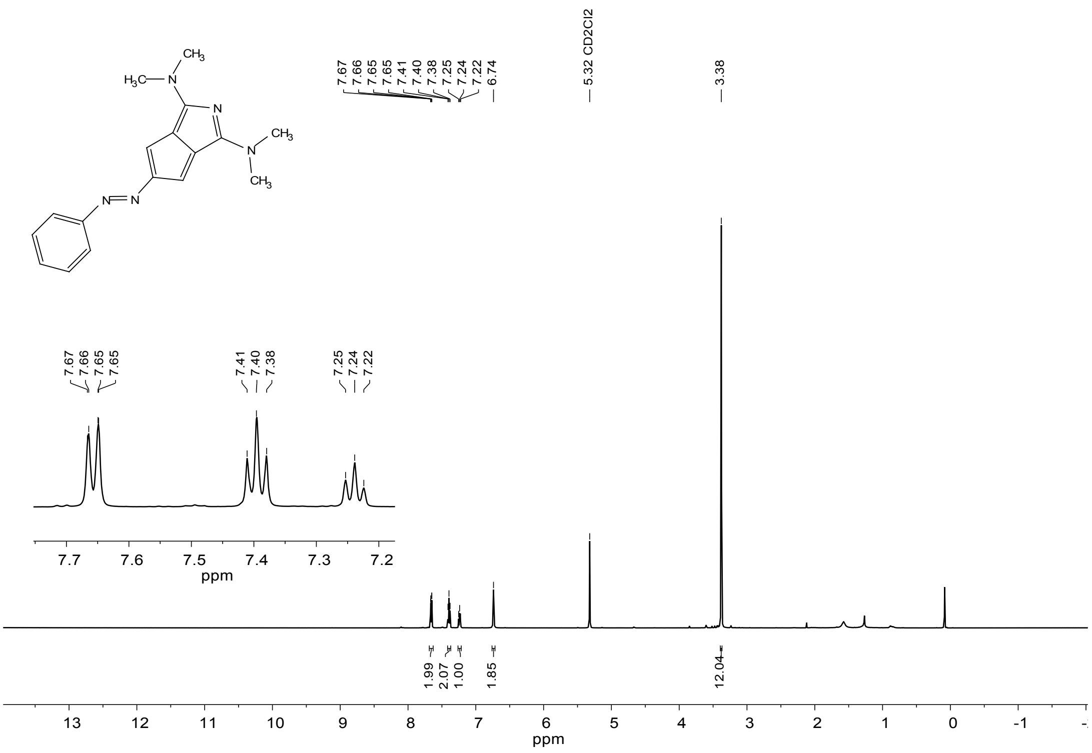

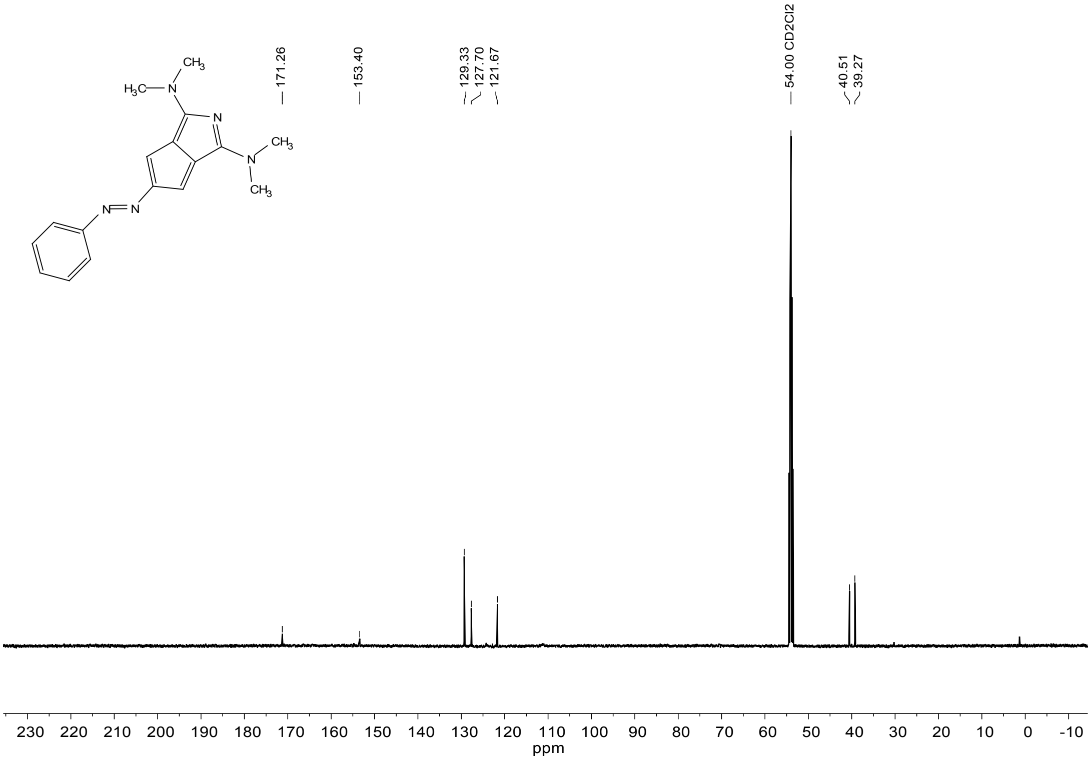

Figure S18. ${}^{1}H$ NMR (top, CD_2Cl_2, 500 MHz) and ${}^{13}C\{{}^{1}H\}$ NMR (bottom, CD_2Cl_2, 126 MHz) spectra of **13**.

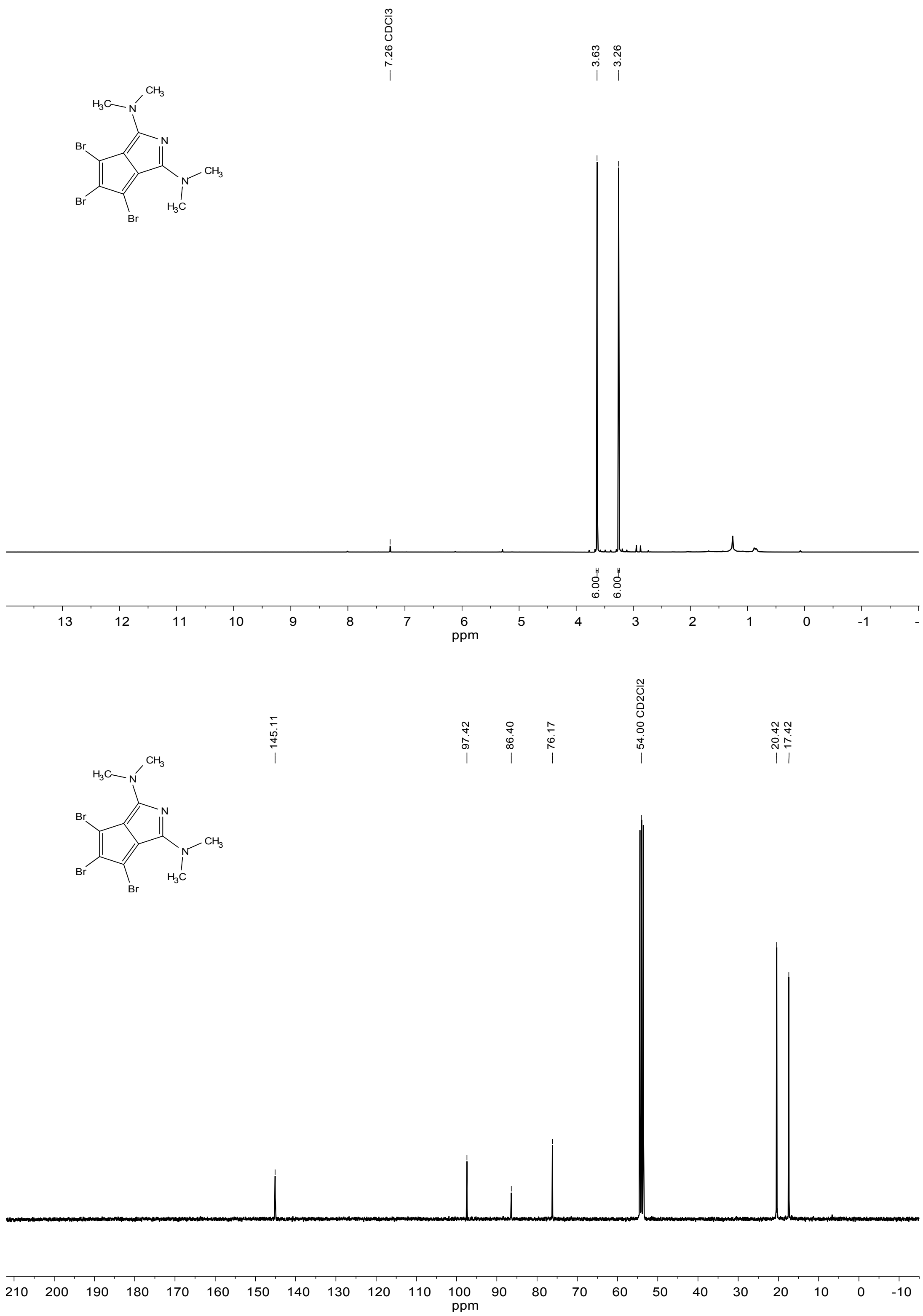

Figure S19. ^{1}H NMR (top, $CDCl_3$, 500 MHz) and $^{13}C\{^{1}H\}$ NMR (bottom, CD_2Cl_2, 126 MHz) spectra of **14**.

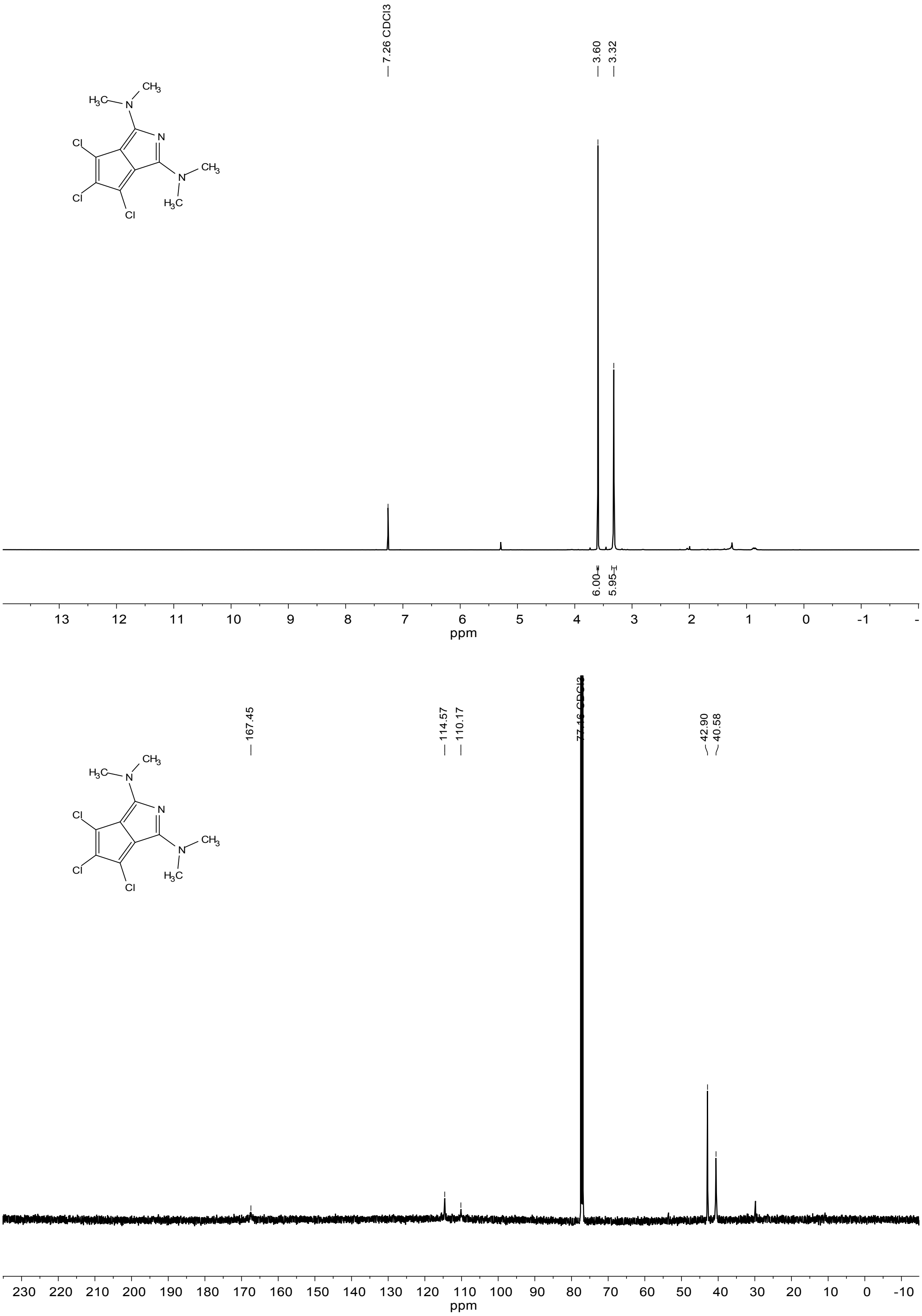

Figure S20. ^{1}H NMR (top, $CDCl_3$, 500 MHz) and $^{13}C\{^{1}H\}$ NMR (bottom, $CDCl_3$, 126 MHz) spectra of **15**.

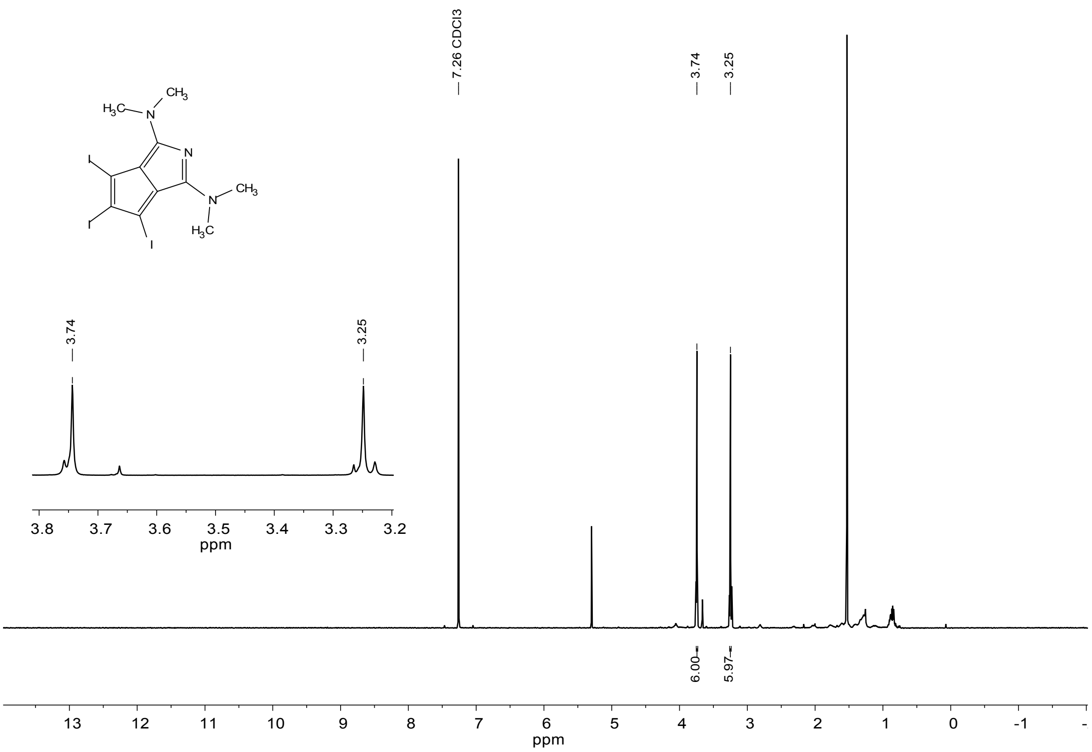

Figure S21. ^{1}H NMR ($CDCl_3$, 500 MHz) spectrum of **16**.

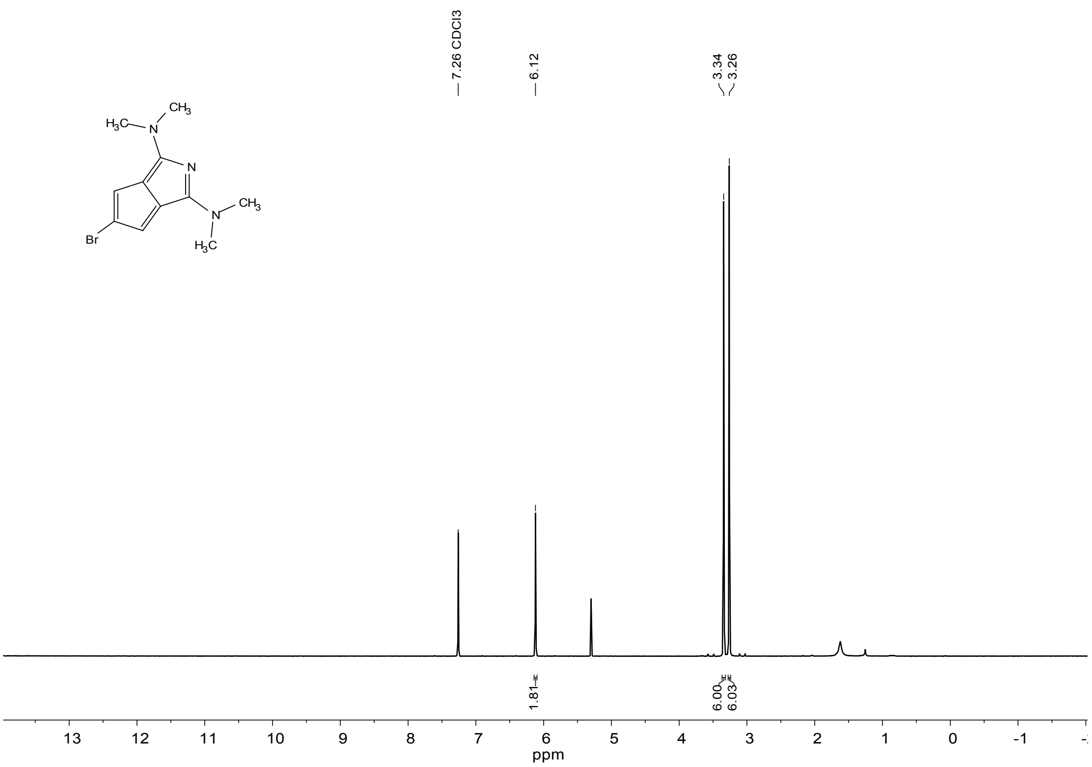

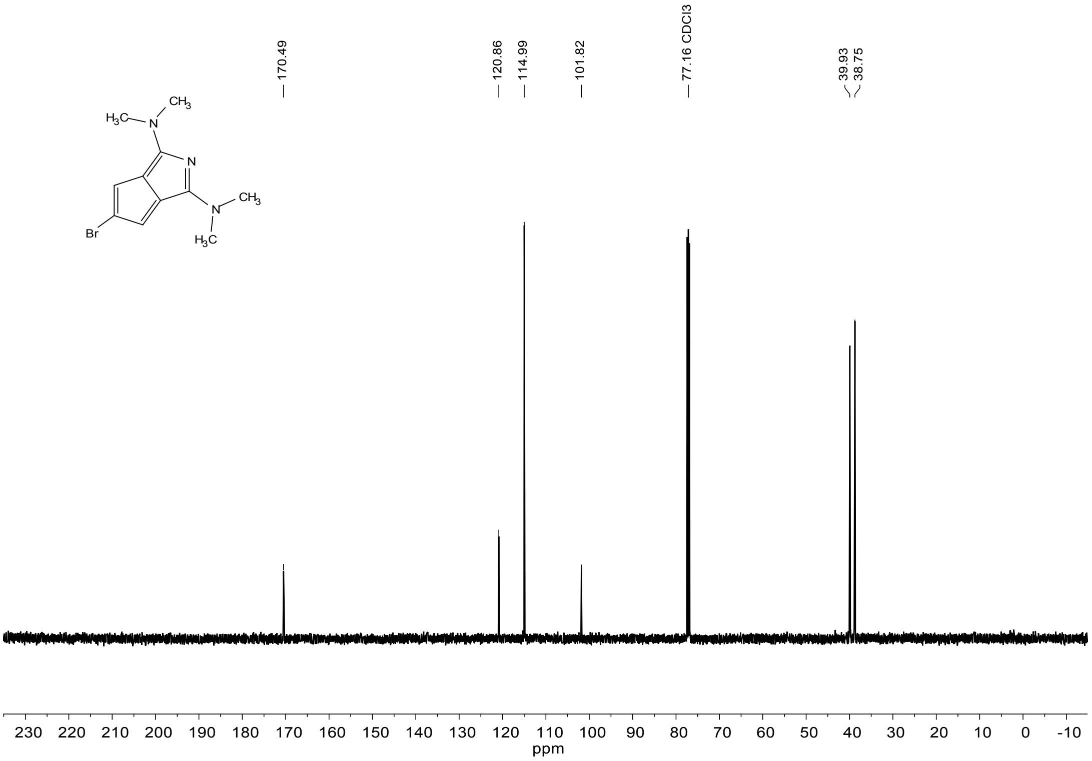

Figure S22. ^{1}H NMR (top, $CDCl_3$, 300 MHz) and $^{13}C\{^1H\}$ NMR (bottom, $CDCl_3$, 126 MHz) spectra of **17**.

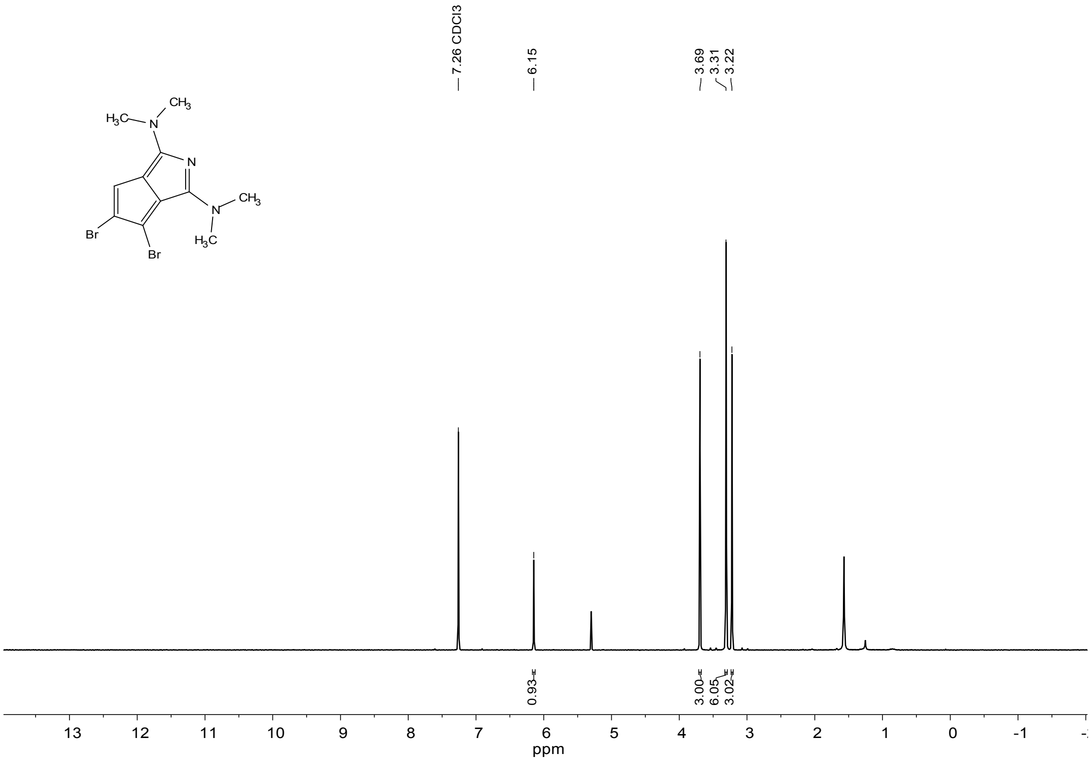

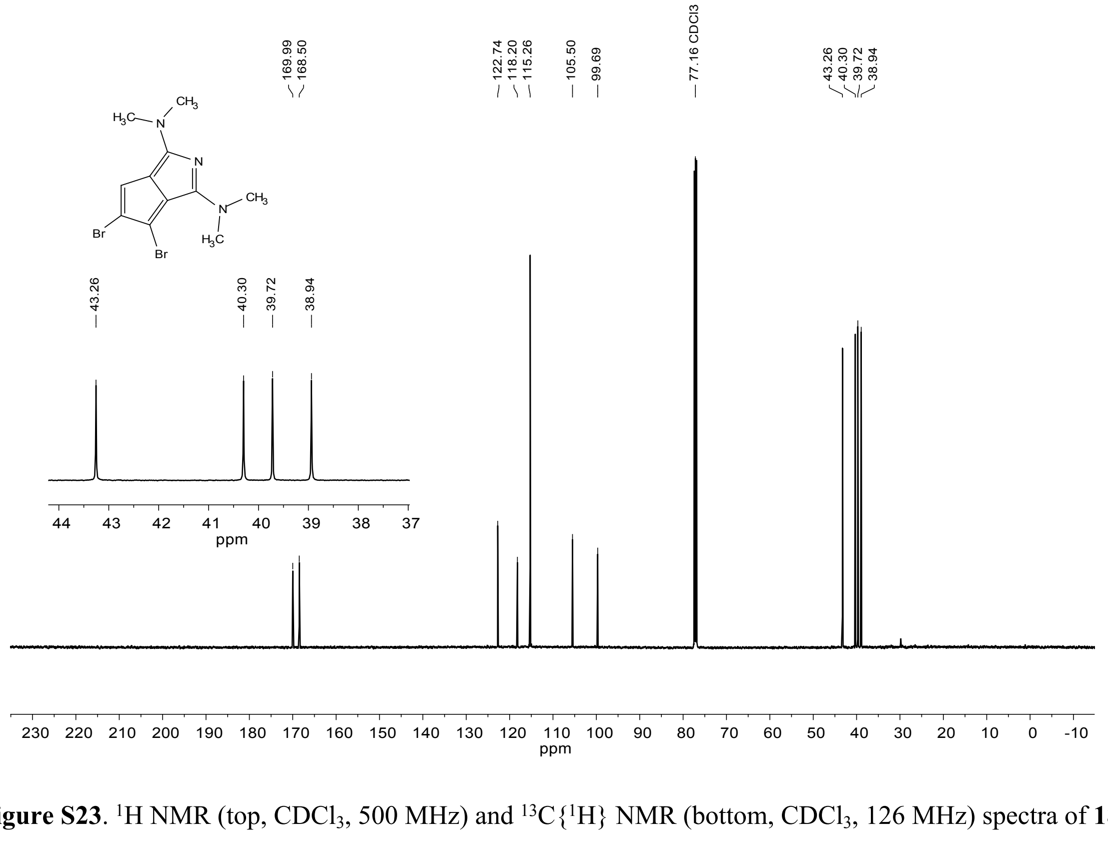

Figure S23. ^{1}H NMR (top, $CDCl_3$, 500 MHz) and $^{13}C\{^{1}H\}$ NMR (bottom, $CDCl_3$, 126 MHz) spectra of **18**.

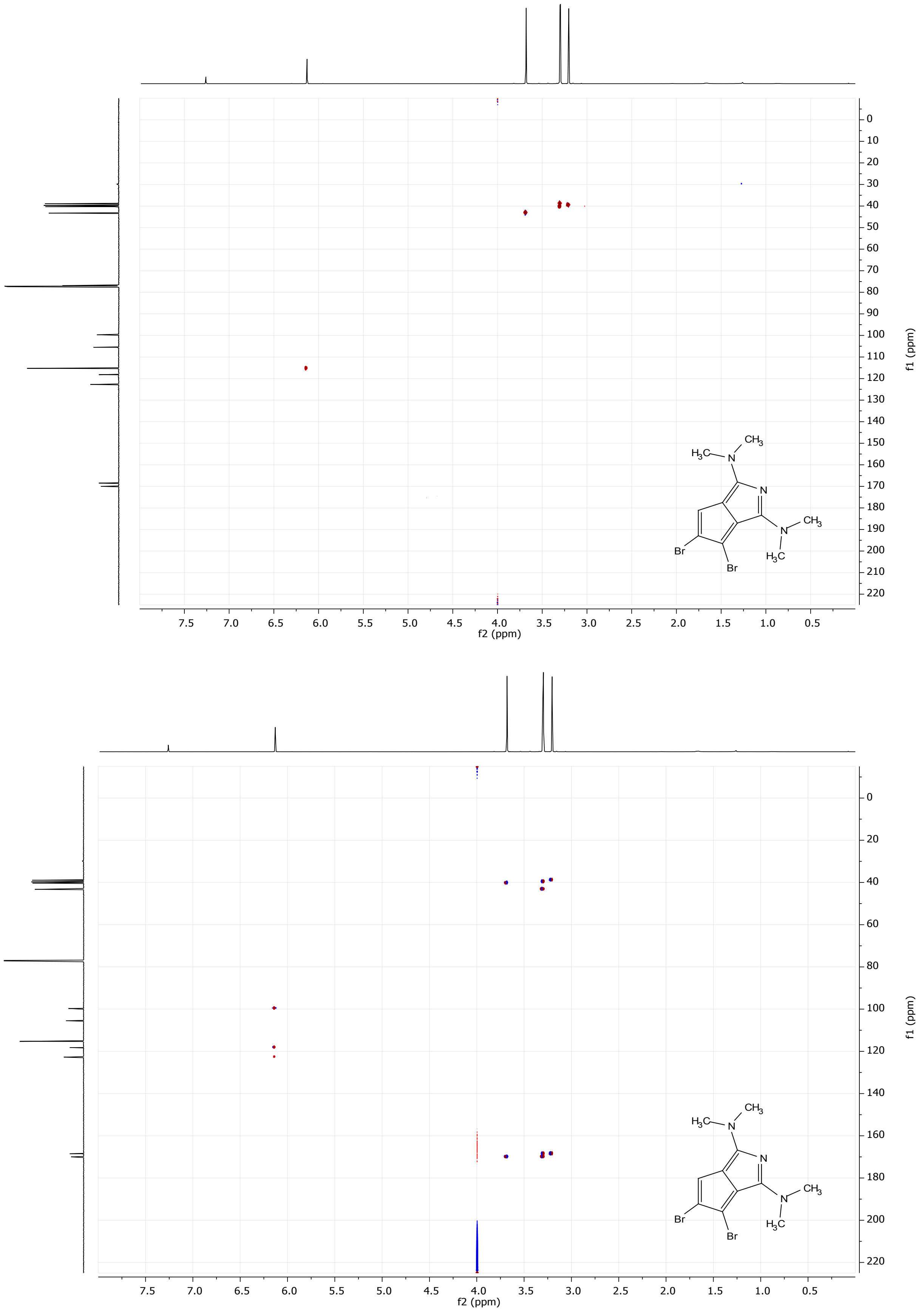

Figure S24. ^{1}H – ^{13}C HSQC and ^{1}H – ^{13}C HMBC spectra of **18** in $CDCl_3$.

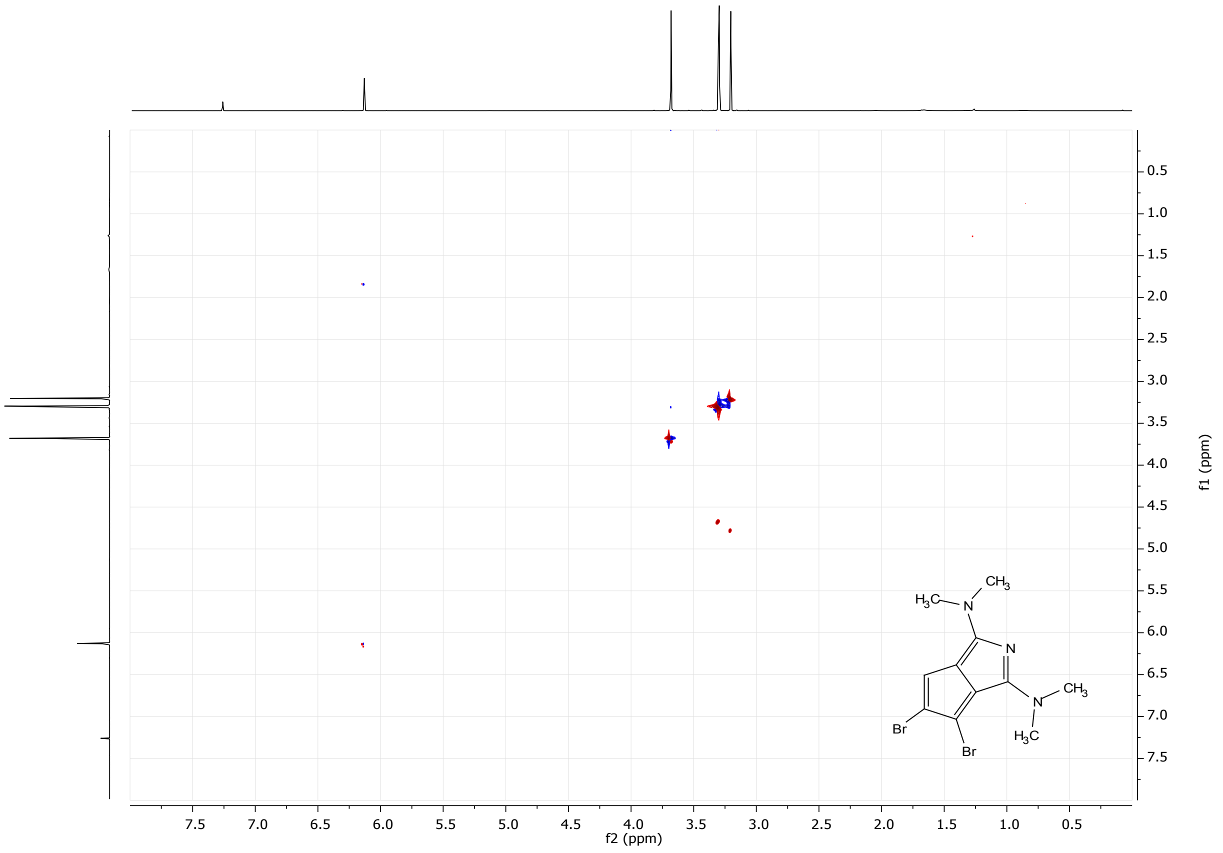

Figure S25. COSY spectrum of **18** in $CDCl_3$

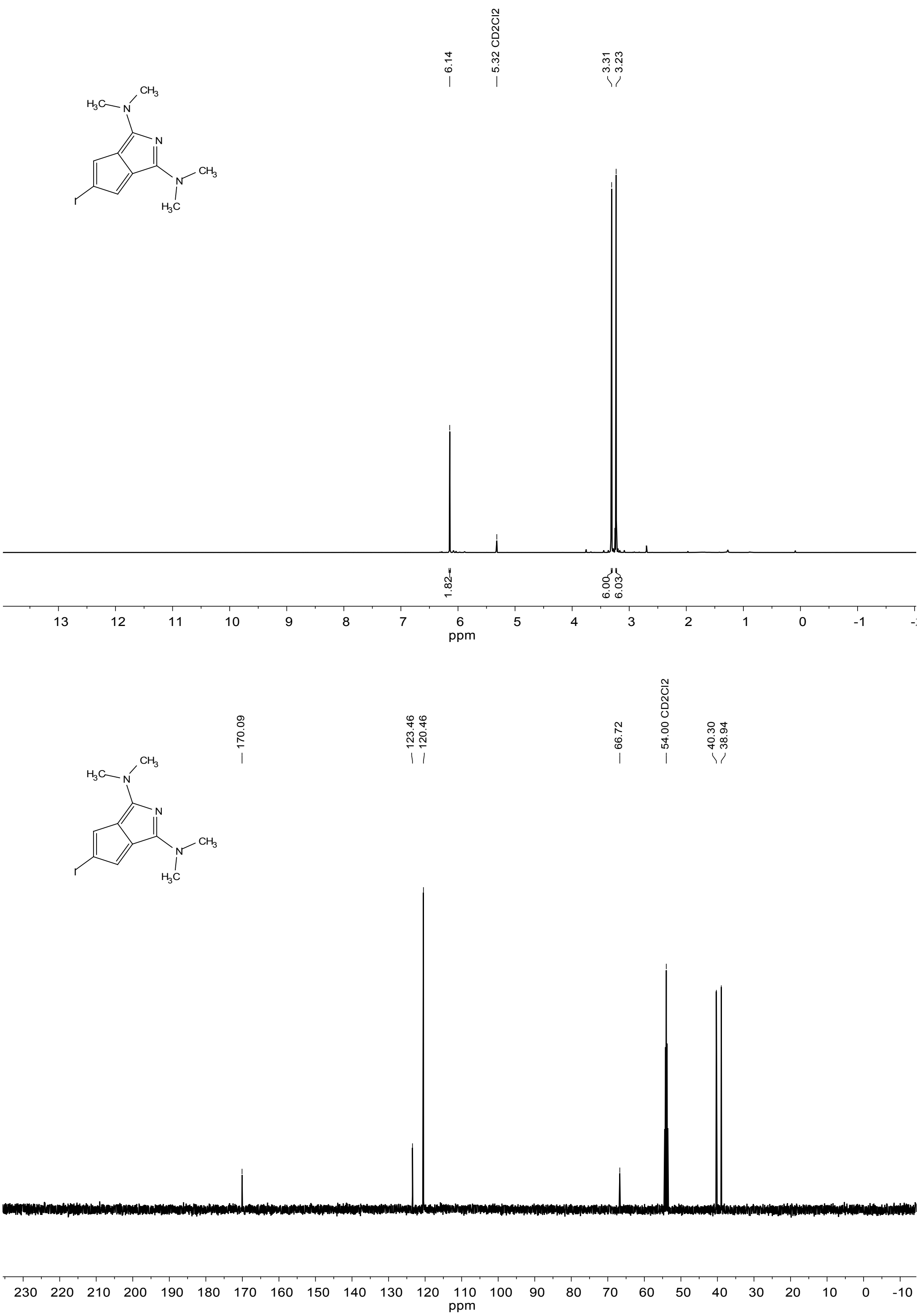

Figure S26. ^{1}H NMR (top, CD_2Cl_2, 500 MHz) and $^{13}C\{^1H\}$ NMR (bottom, CD_2Cl_2, 126 MHz) spectra of **20**.

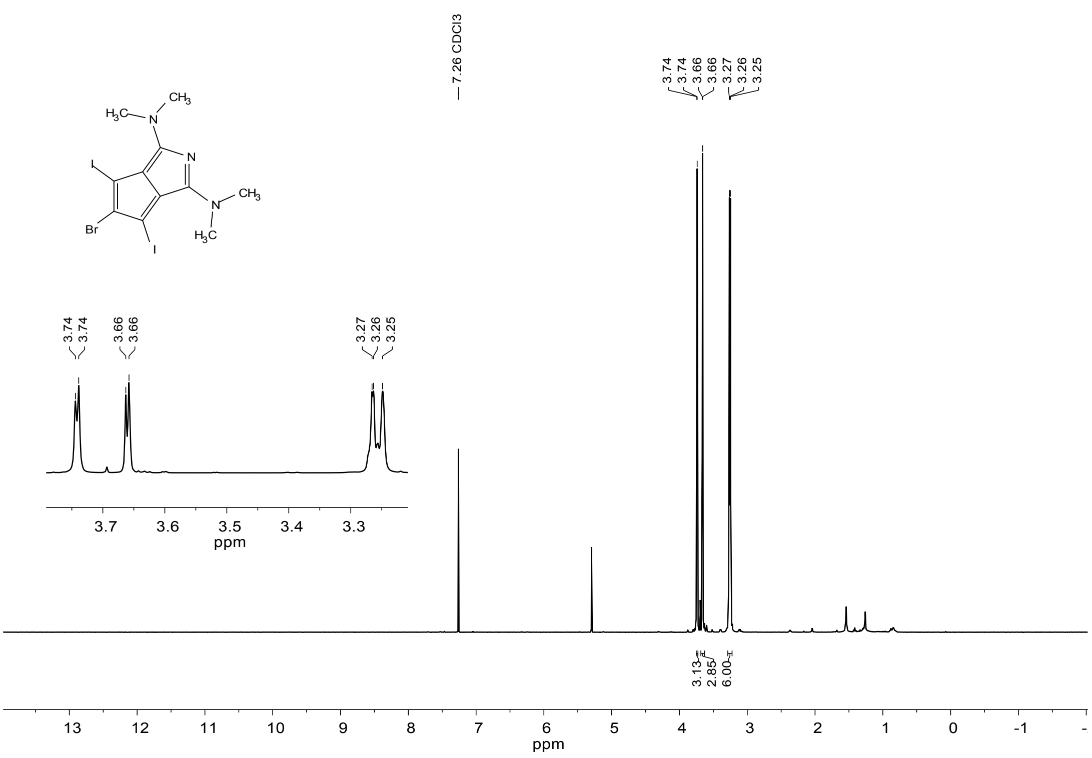

Figure S27. ^{1}H NMR ($CDCl_3$, 500 MHz) spectrum of **21**.

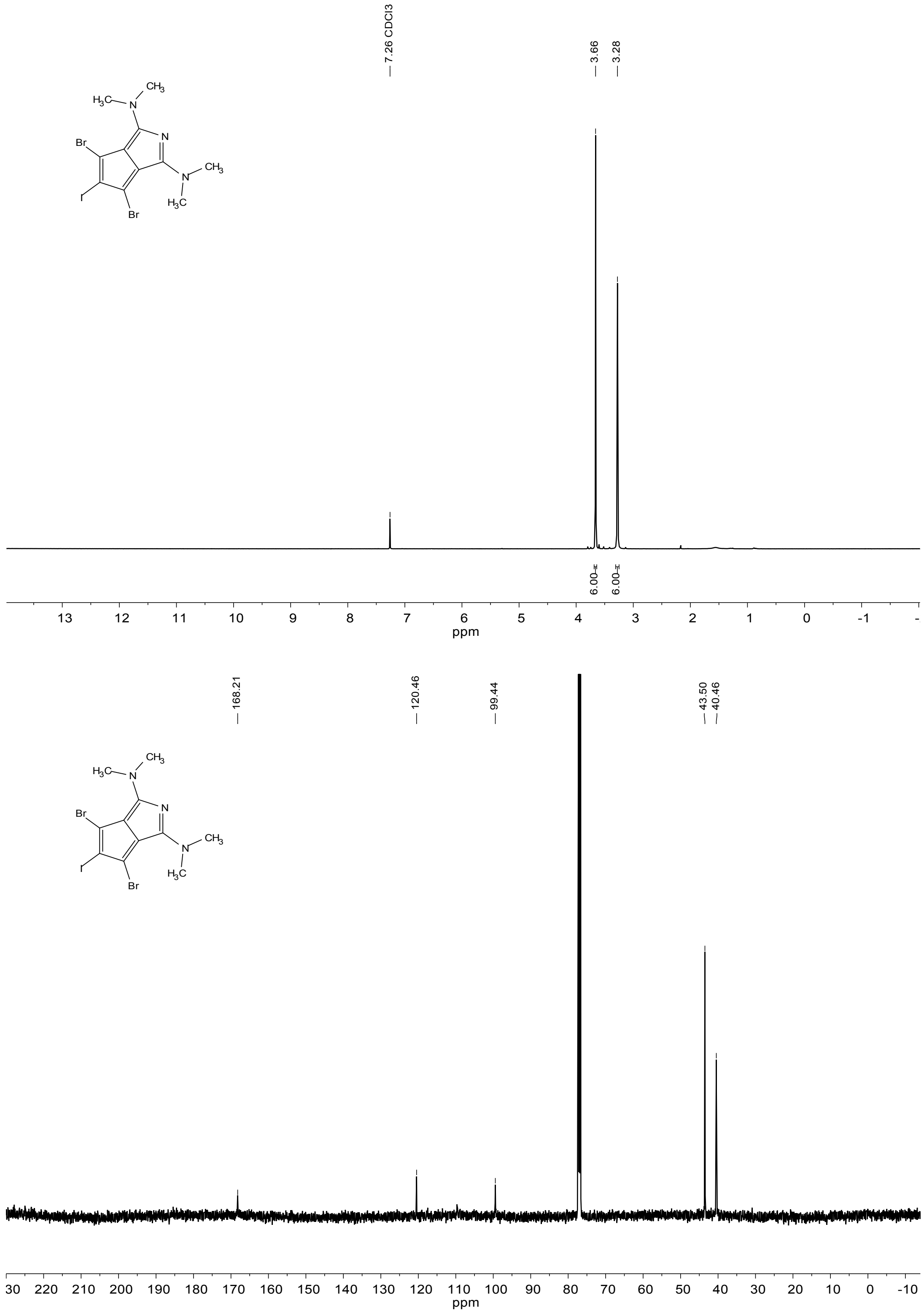

Figure S28. ^1H NMR (top, $CDCl_3$, 500 MHz) and $^{13}C\{^1H\}$ NMR (bottom, $CDCl_3$, 126 MHz) spectra of **22**.

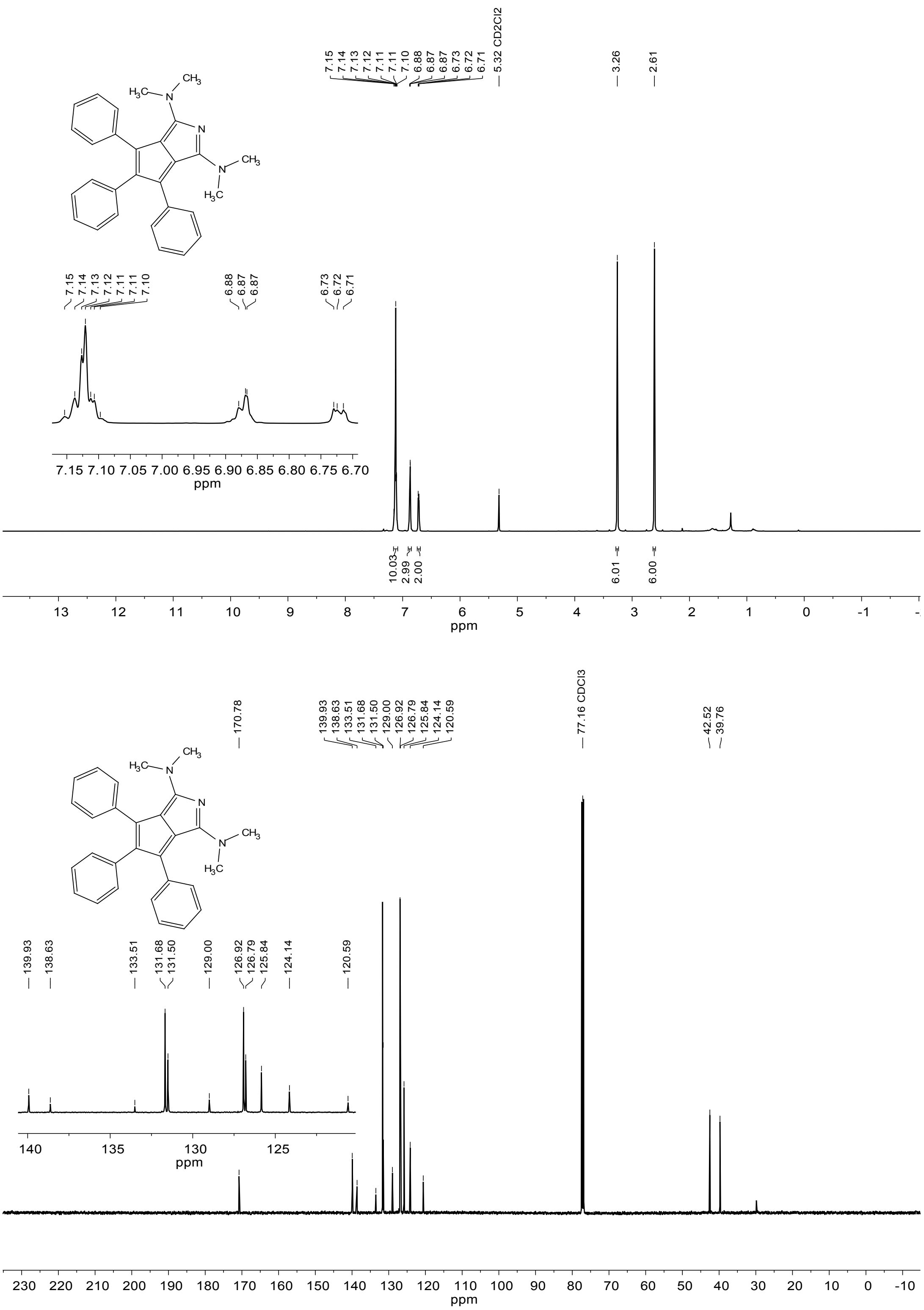

Figure S29. ^1H NMR (top, CD_2Cl_2, 500 MHz) and $^{13}C\{^1H\}$ NMR (bottom, $CDCl_3$, 126 MHz) spectra of **23**.

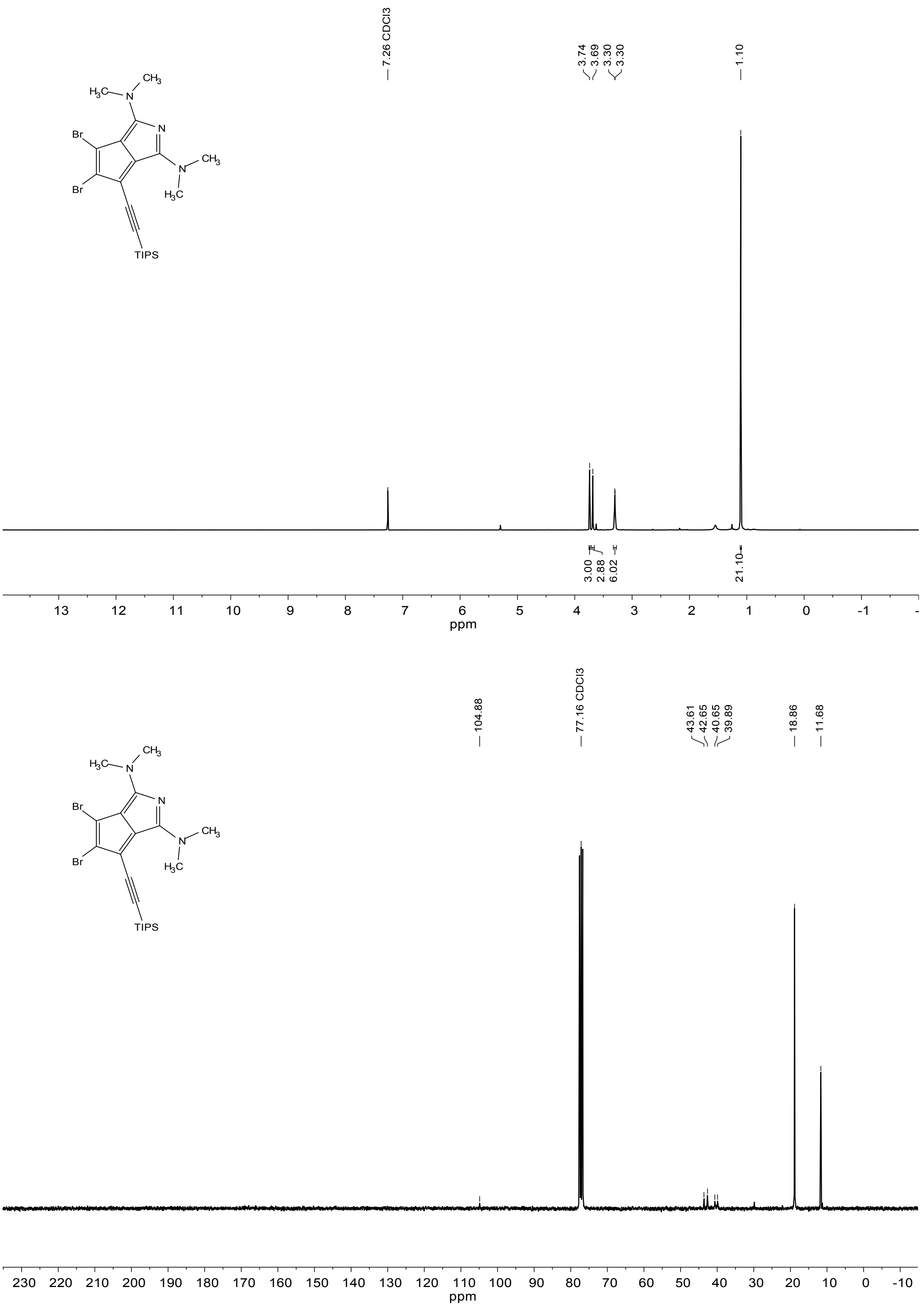

Figure S30. ^1H NMR (top, $CDCl_3$, 500 MHz) and $^{13}C\{^1H\}$ NMR (bottom, $CDCl_3$, 126 MHz) spectra of **25**.

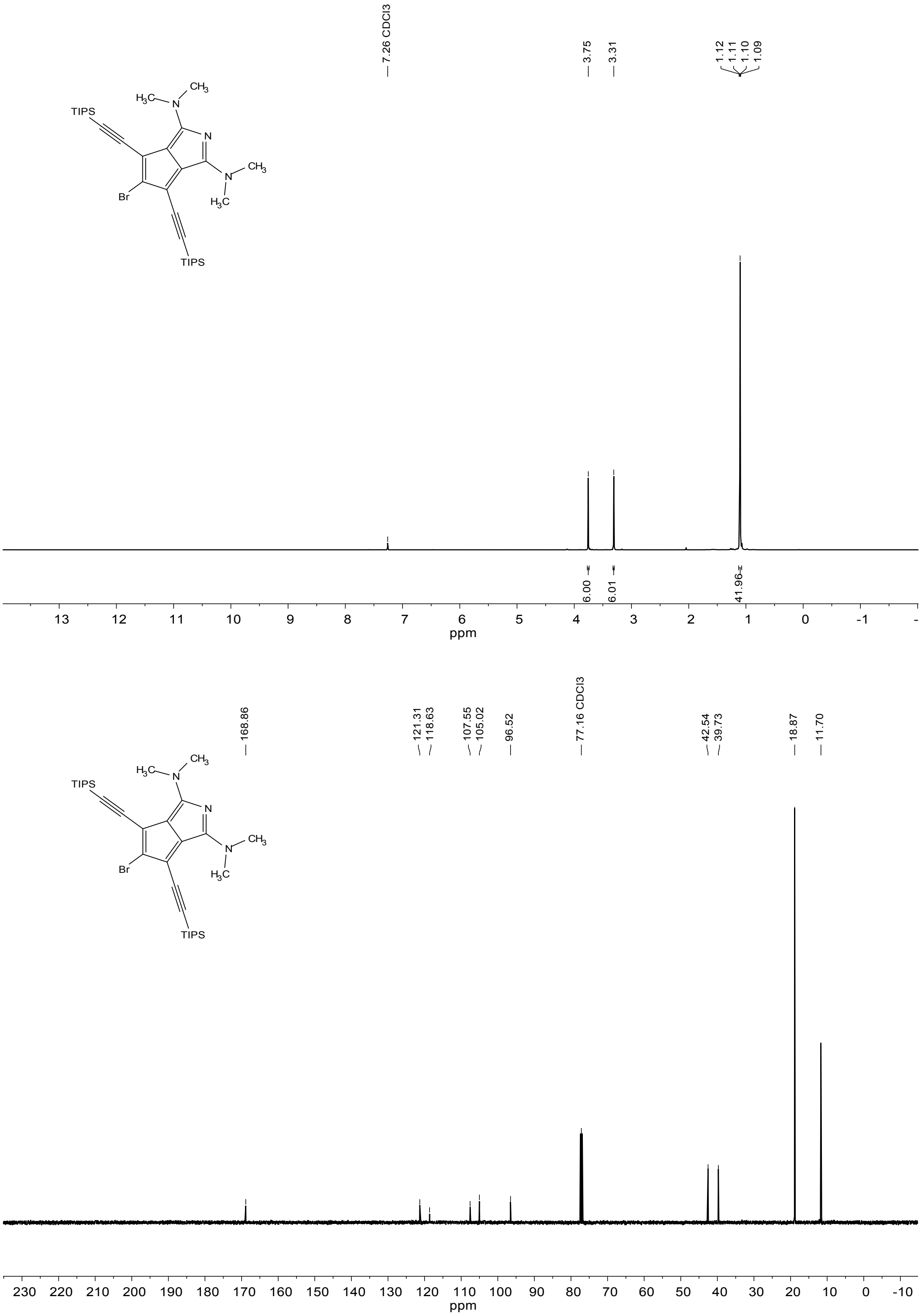

Figure S31. ^{1}H NMR (top, $CDCl_3$, 500 MHz) and $^{13}C\{^{1}H\}$ NMR (bottom, $CDCl_3$, 126 MHz) spectra of **26**.

S4 HRMS spectra

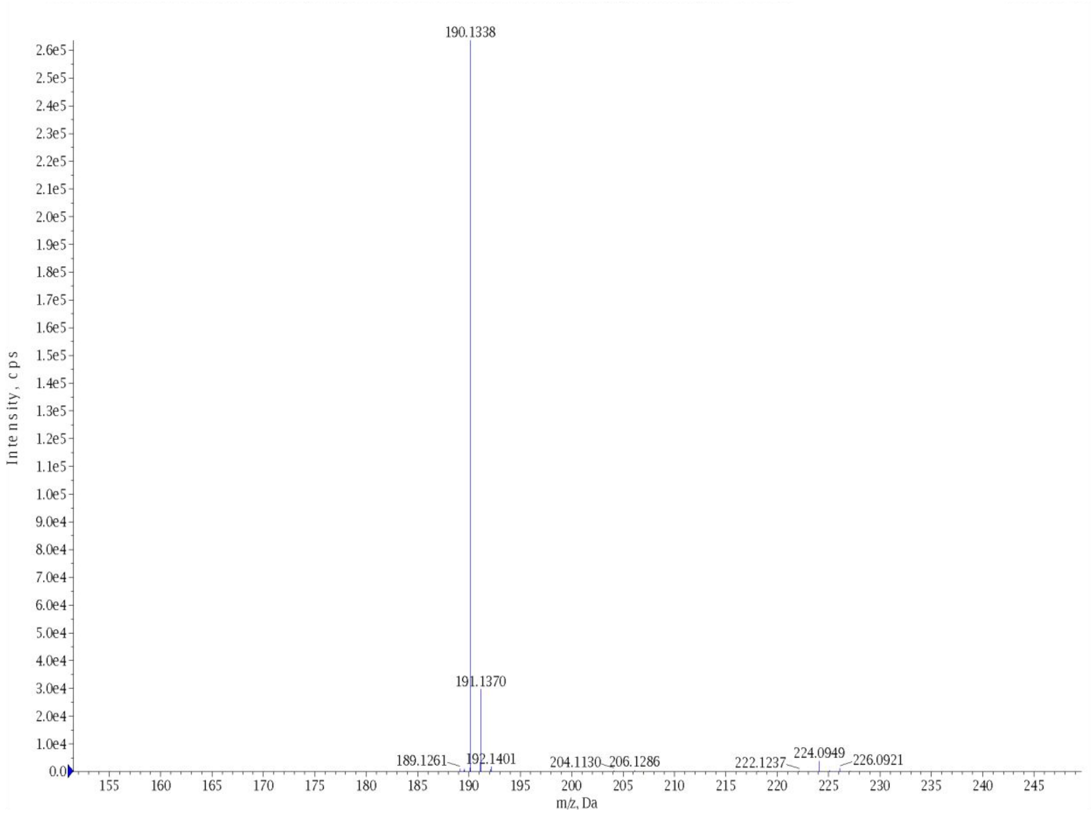

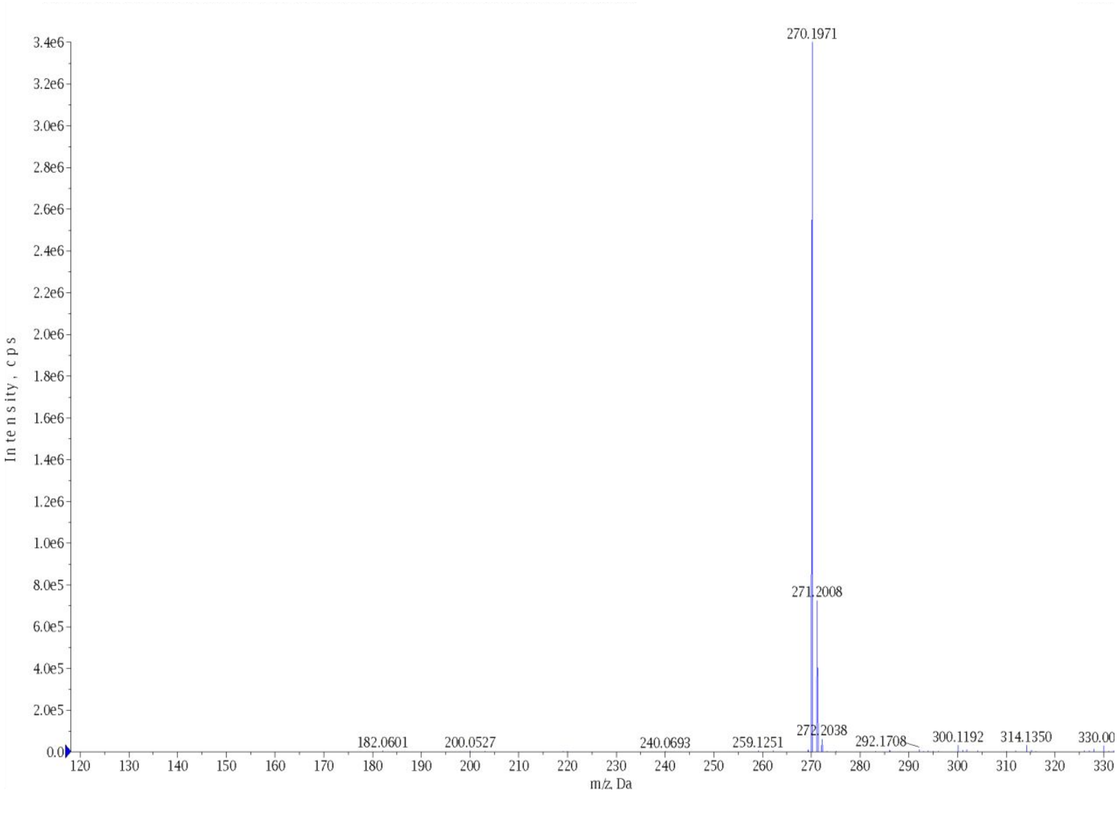

Figure S32. HRMS spectra of **3** (top) and **8** (bottom).

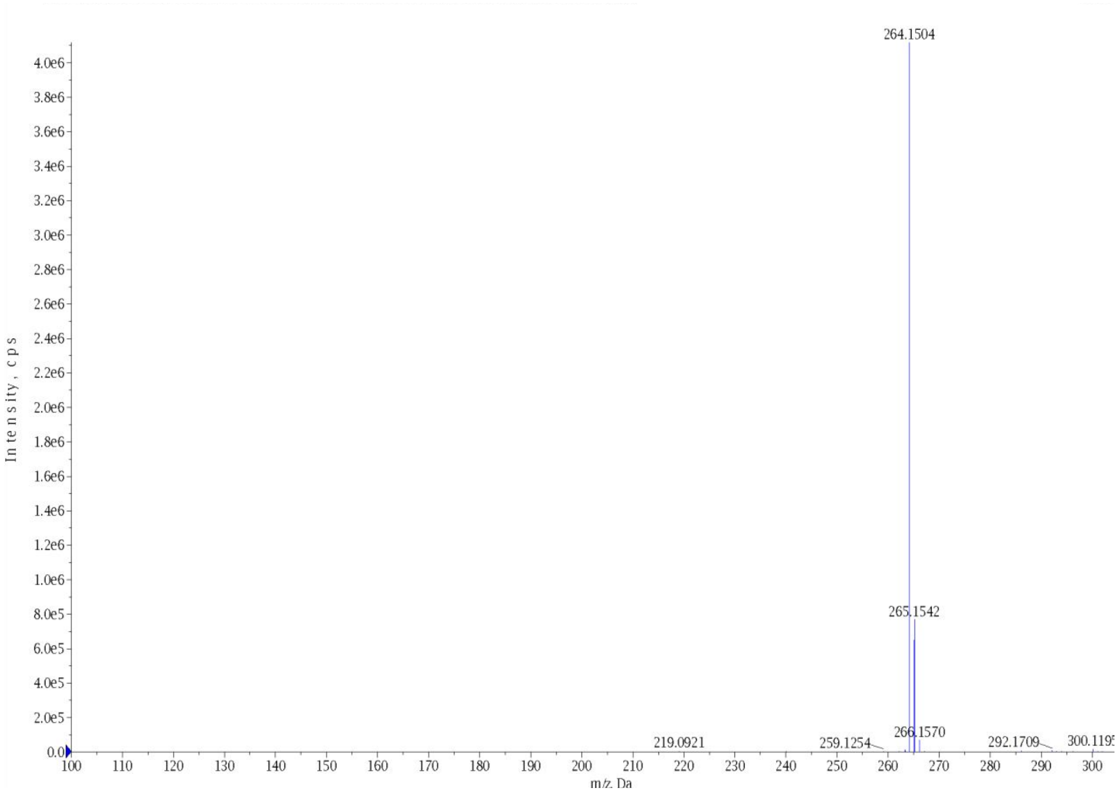

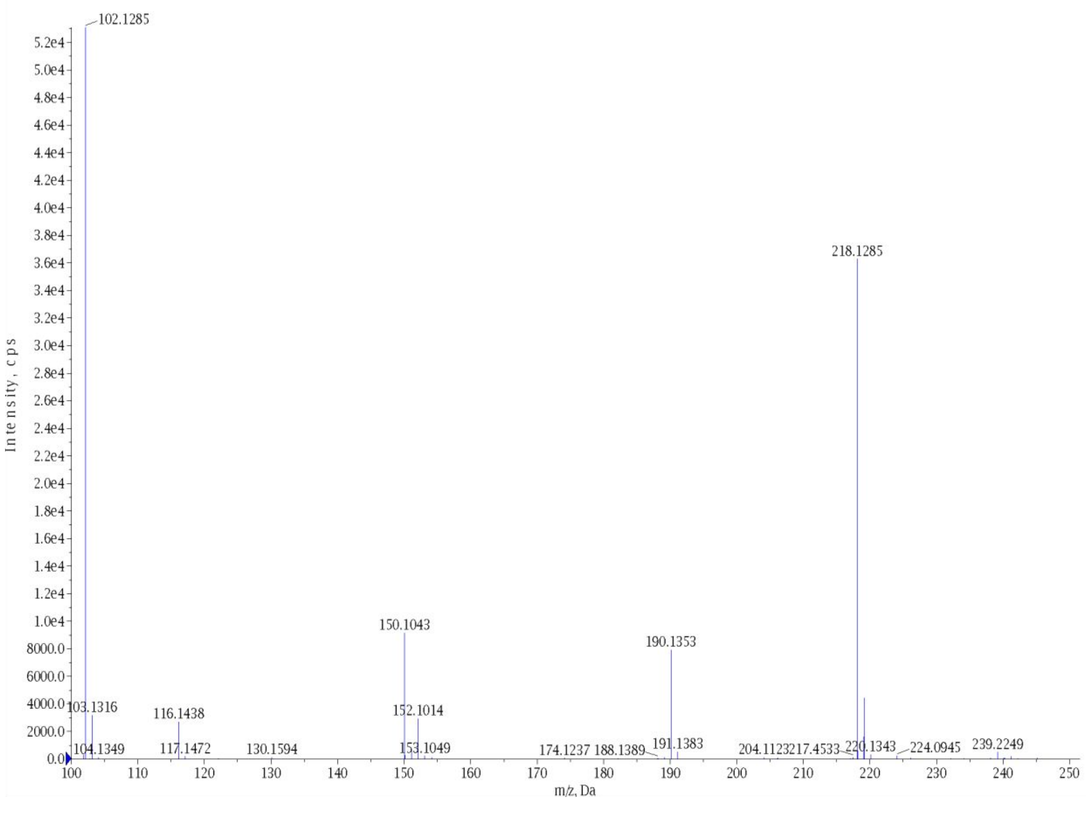

Figure S33. HRMS spectra of **11** (top) and **12** (bottom).

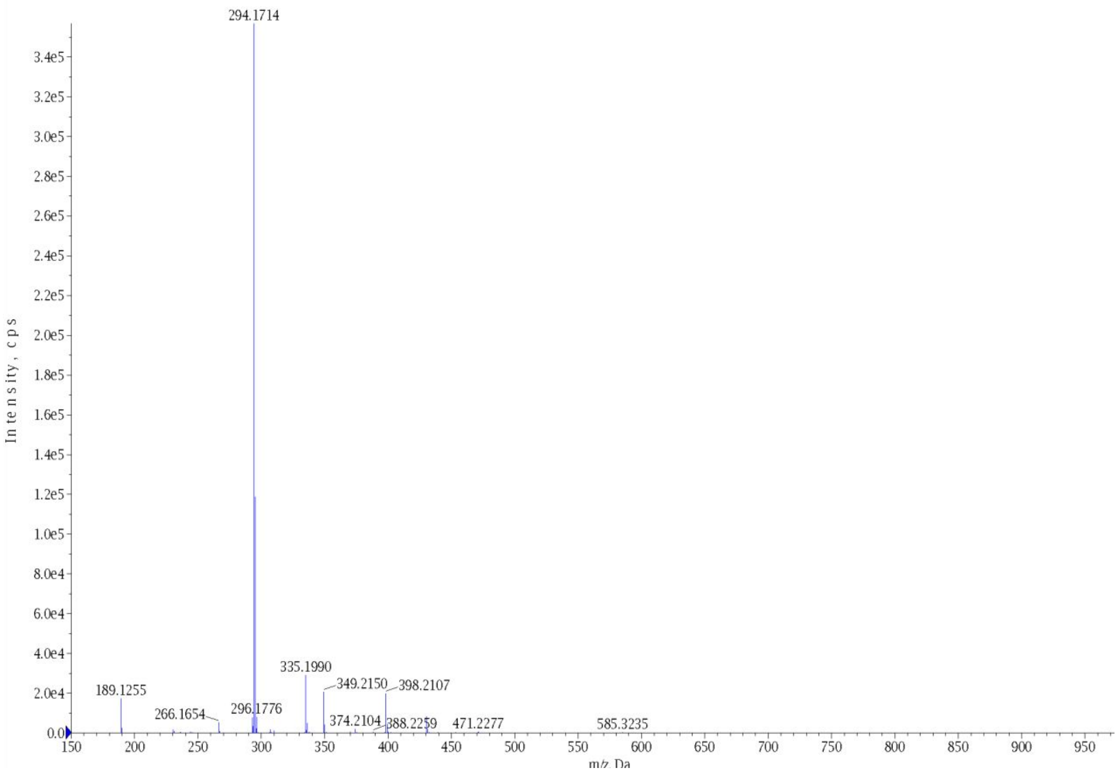

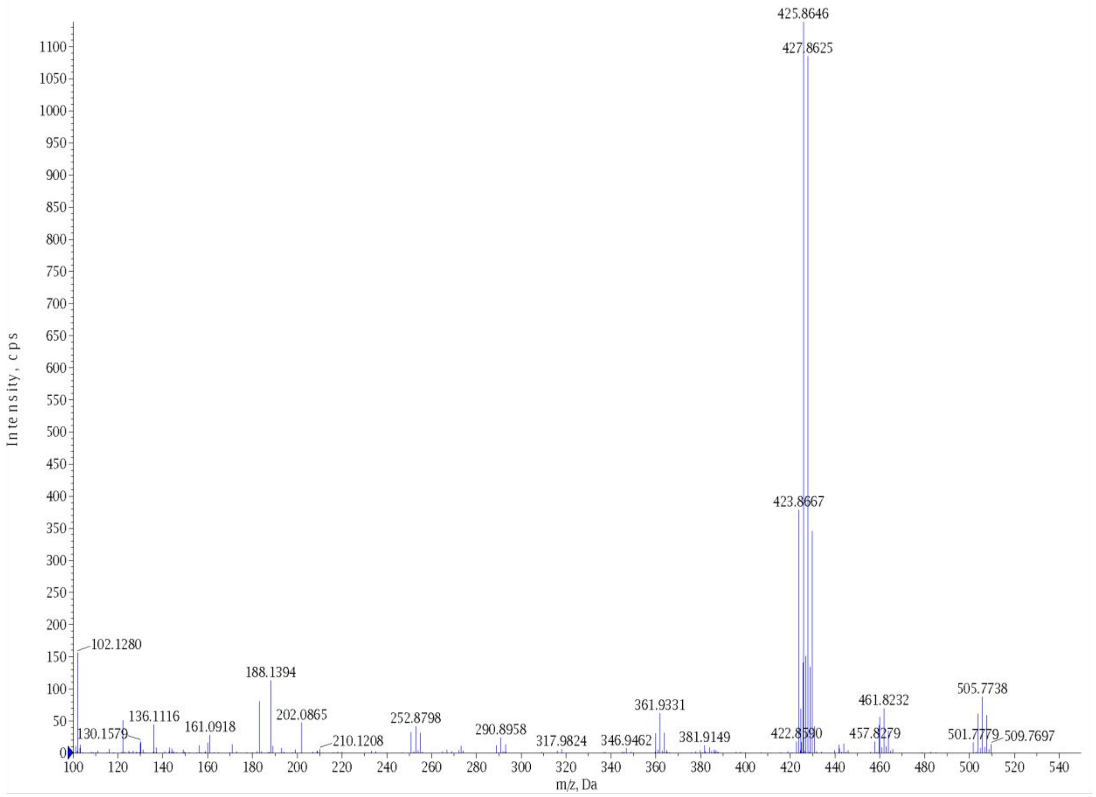

Figure S34. HRMS spectra of **13** (top) and **14** (bottom).

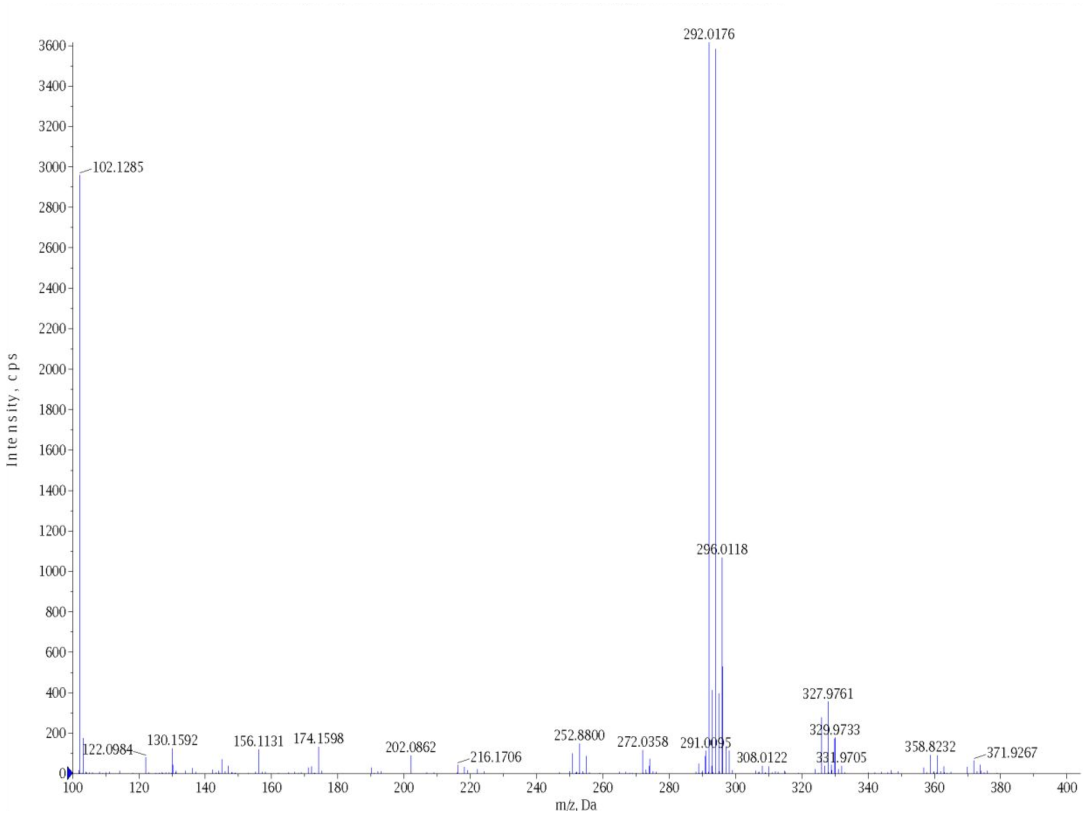

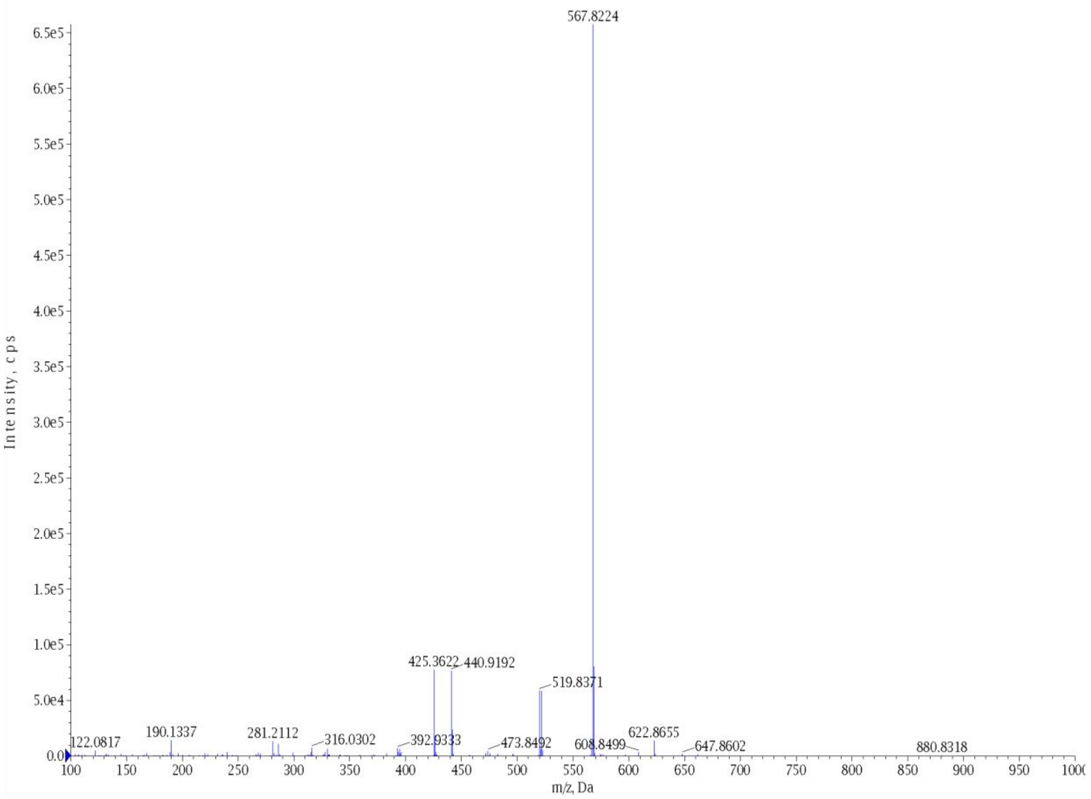

Figure S35. HRMS spectra of **15** (top) and **16** (bottom).

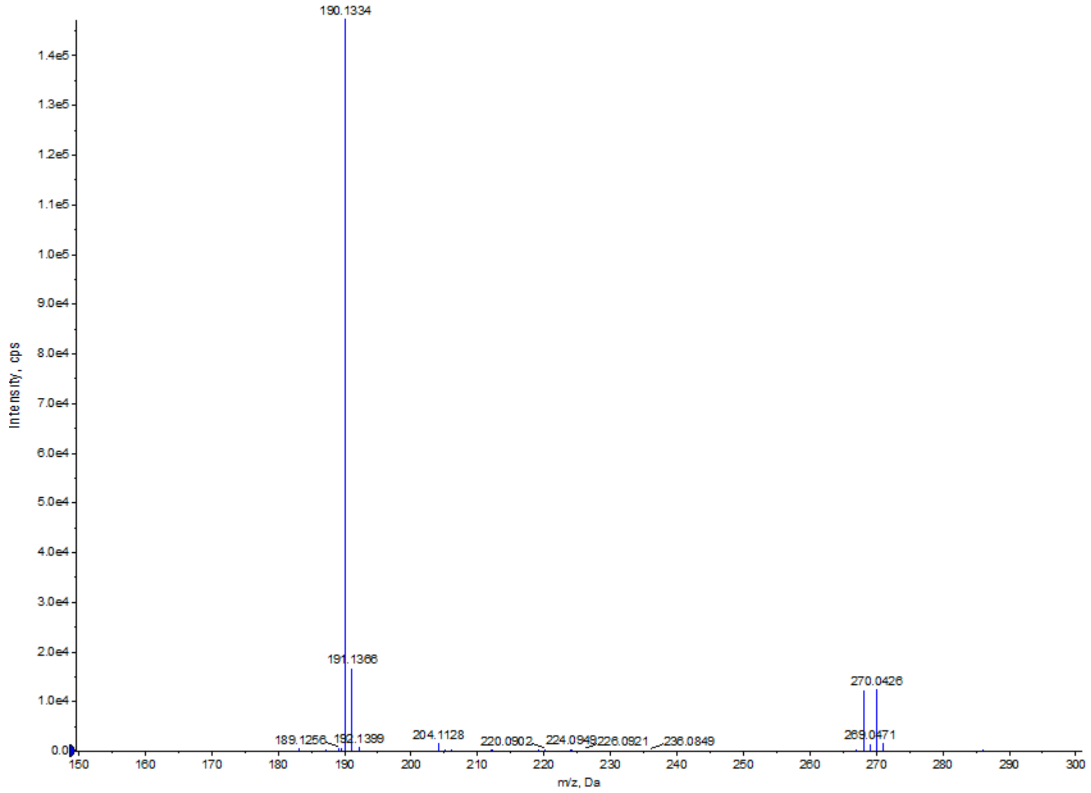

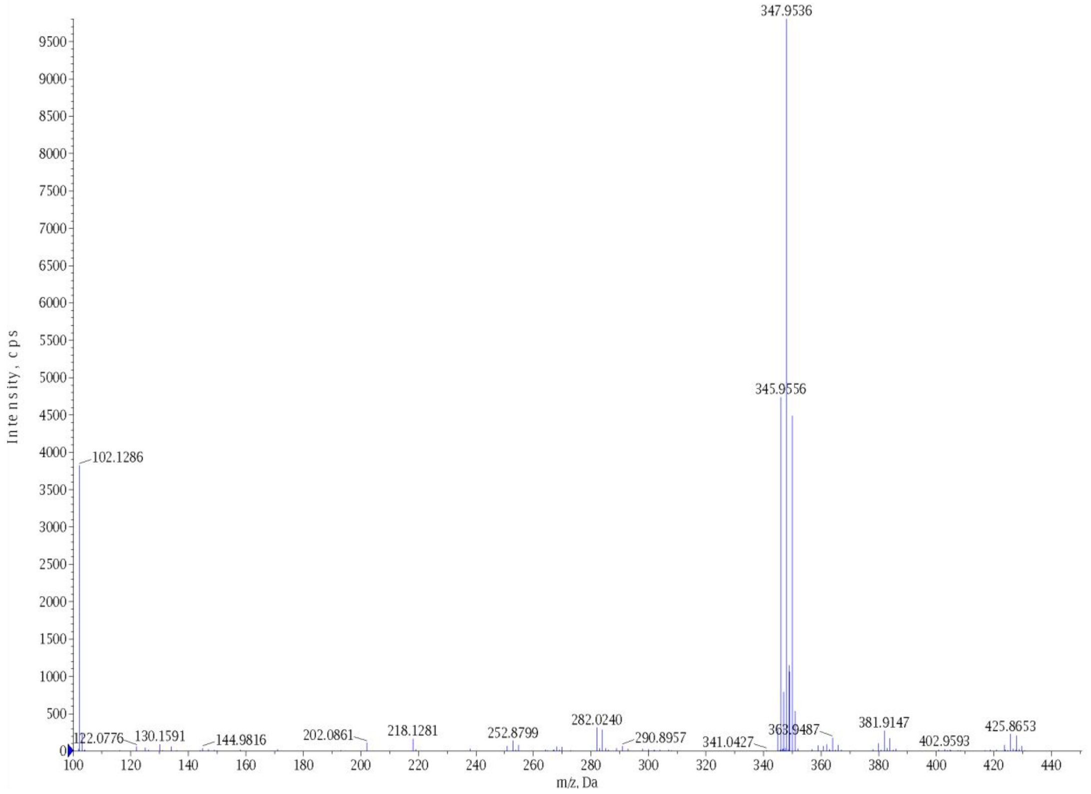

Figure S36. HRMS spectra of **17** (top) and **18** (bottom).

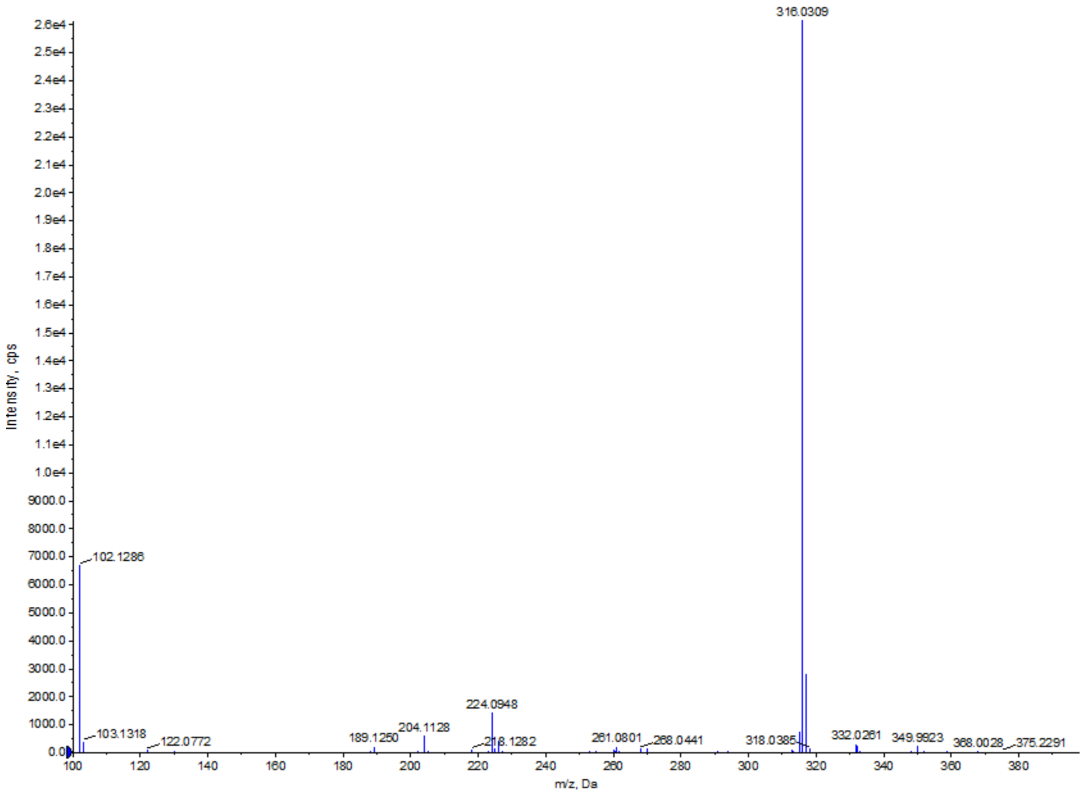

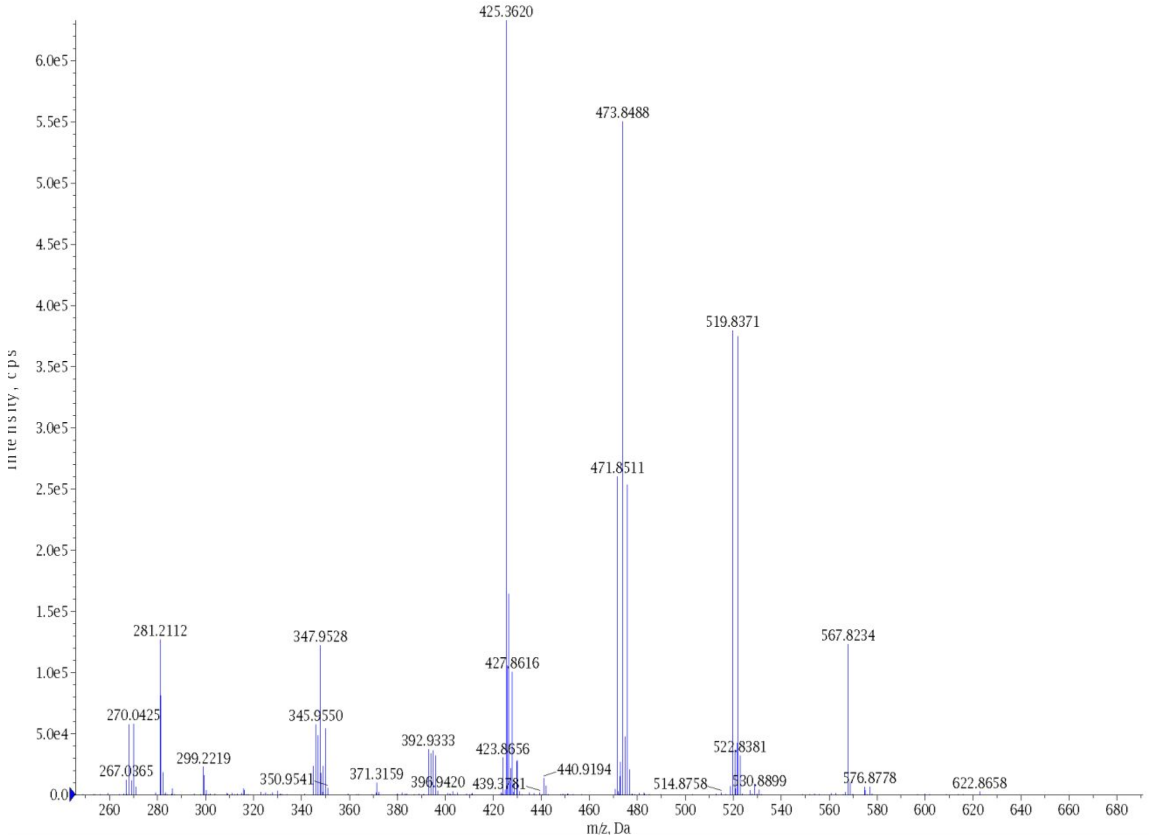

Figure S37. HRMS spectra of **20** (top) and **21** (bottom).

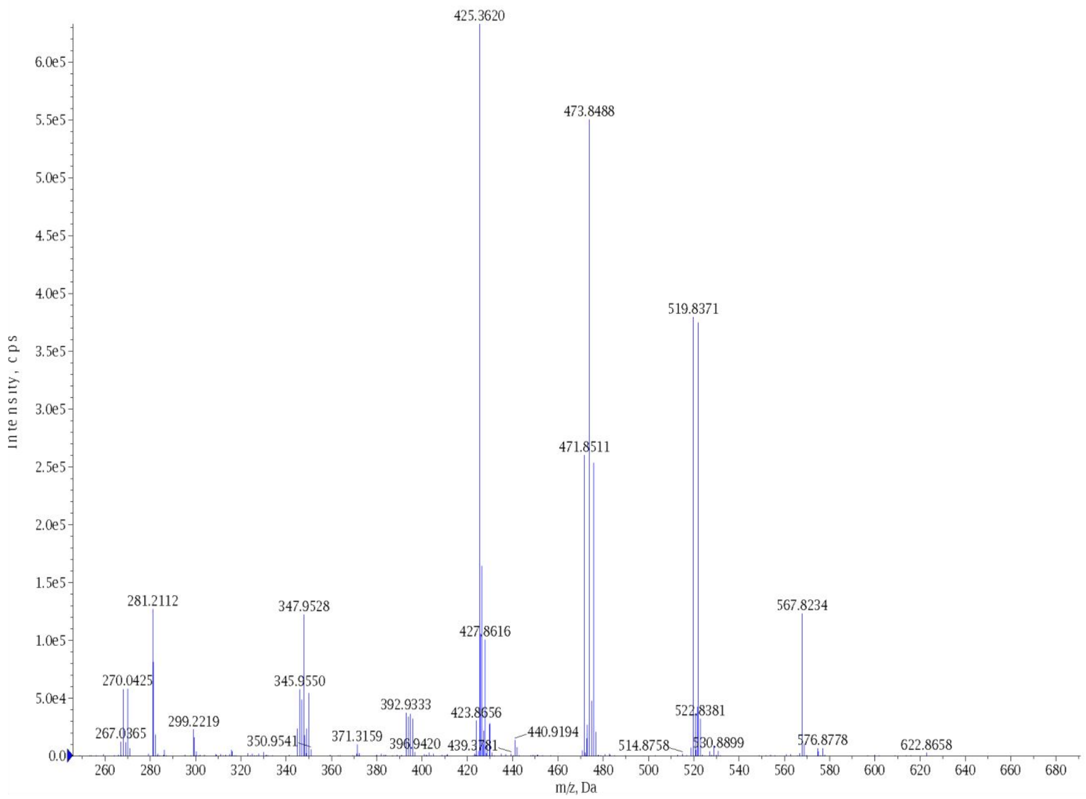

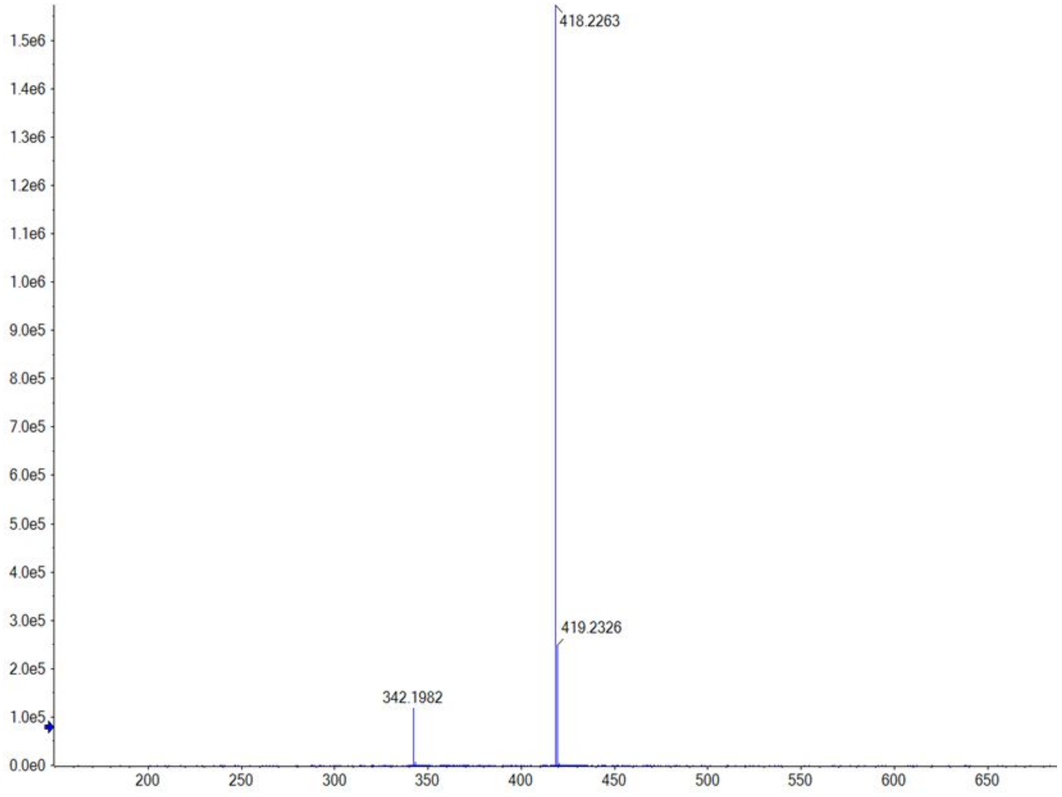

Figure S38. HRMS spectra of **22** (top) and **23** (bottom).

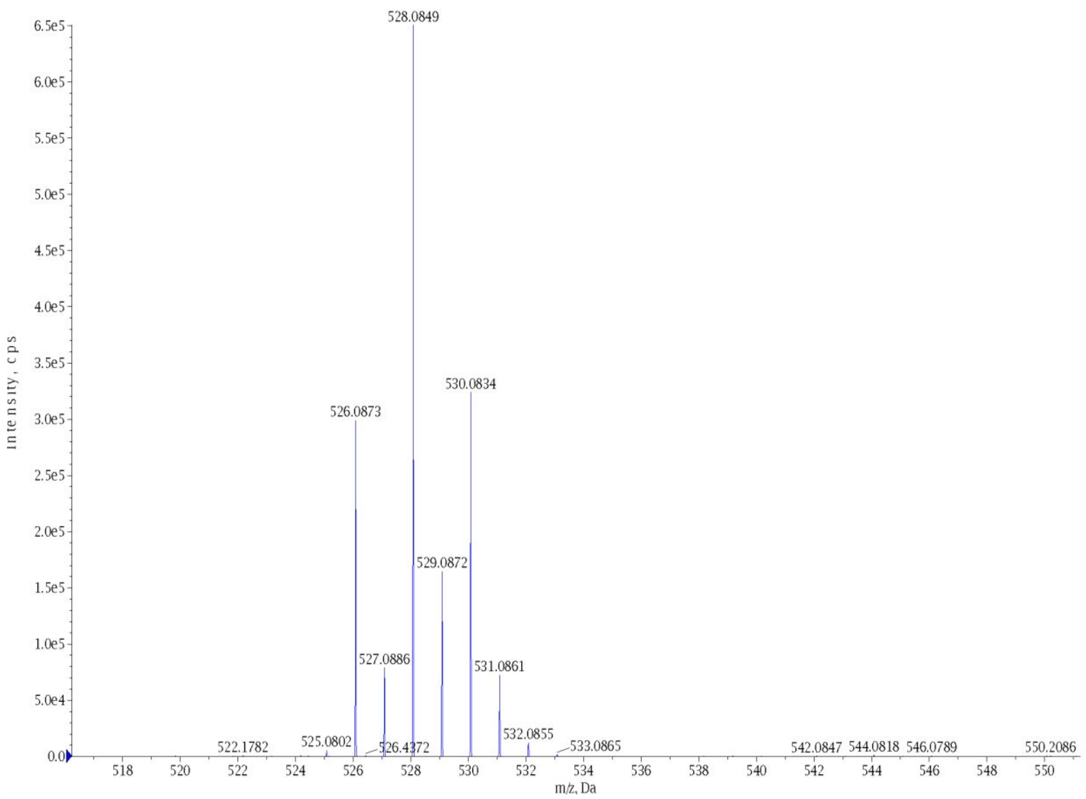

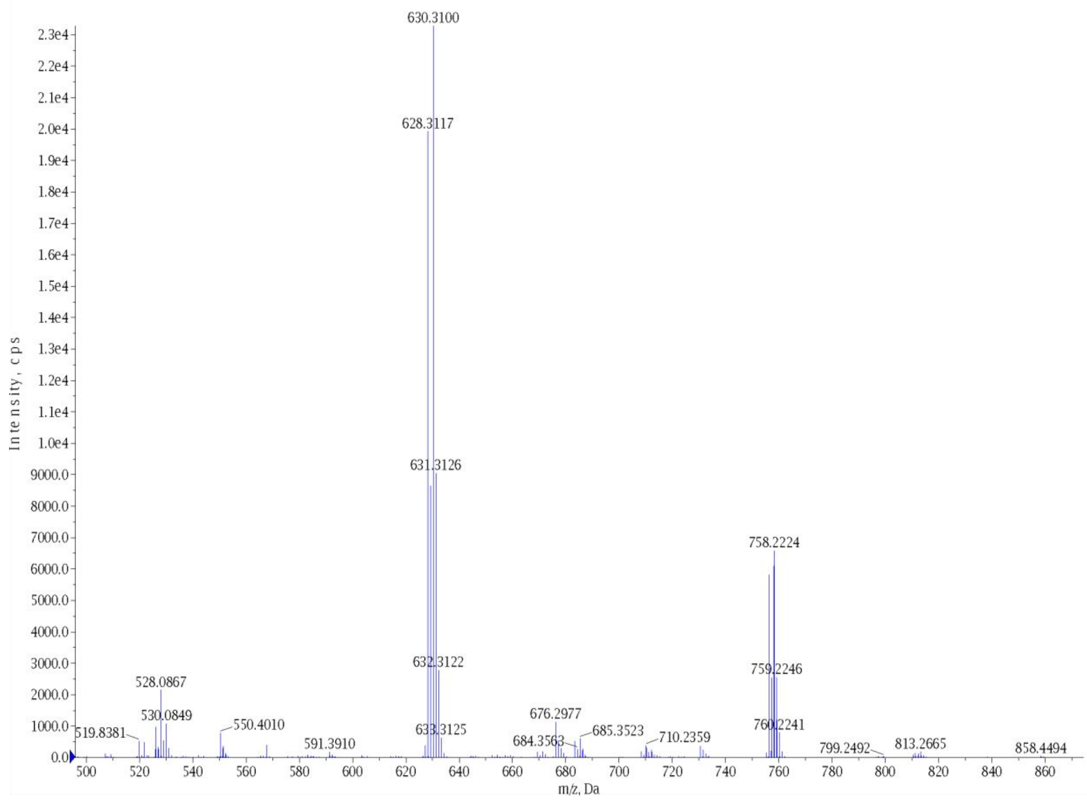

Figure S39. HRMS spectra of **25** (top) and **26** (bottom).

S5 Cartesian coordinates

1

Pentalene

E = -308.447609393 Hartree

Number of imaginary frequencies = 0

C	-1.223066	1.219776	0.000066
C	0.038280	0.727945	-0.000047
C	-0.038256	-0.727973	-0.000045
C	-1.465667	-1.095249	0.000008
C	1.465622	1.095219	-0.000035
C	2.170151	-0.061005	0.000000
C	1.223075	-1.219740	0.000026
H	1.869981	2.097237	0.000124
H	3.246990	-0.158162	0.000098
H	1.533367	-2.257263	-0.000018
H	-1.870162	-2.097230	0.000067
H	-1.533486	2.257288	-0.000061
C	-2.170096	0.061021	0.000000
H	-3.246950	0.158169	-0.000054

2

Cyclopenta[*c*]pyrrole

E = -324.492353838 Hartree

Number of imaginary frequencies = 0

C	-1.264557	-1.154229	0.000147
C	0.020269	-0.723285	-0.000055
C	-0.042384	0.719377	-0.000071
C	-1.477688	1.030436	-0.000078
N	-2.196390	-0.051052	-0.000008
C	1.439534	-1.100473	-0.000092
C	2.157456	0.054941	-0.000007
C	1.227631	1.210175	0.000099
H	1.838421	-2.104608	-0.000081
H	3.235028	0.141100	0.000134
H	1.541297	2.246605	0.000222
H	-1.937683	2.012026	-0.000046
H	-1.663903	-2.159400	0.000162

3

N[1],*N*[1],*N*[3],*N*[3]-Tetramethylcyclopenta[*c*]pyrrole-1,3-diamine

E =-592.568299265 Hartree

Number of imaginary frequencies = 0

C	-1.084364	-0.388997	-0.032424
C	-0.710383	1.002471	-0.035996
C	0.710291	1.002479	-0.035931
C	1.084316	-0.389103	-0.032417
N	0.000016	-1.214669	-0.019487
C	-1.153396	2.339942	0.032279
C	-0.000017	3.153999	0.065261
C	1.153411	2.339813	0.032327
N	-2.326210	-0.894720	-0.047813
N	2.326235	-0.894698	-0.047843
C	-3.485306	-0.014588	-0.047890
C	-2.571030	-2.327073	0.073182
C	3.485244	-0.014482	-0.047914
C	2.571136	-2.327005	0.073173
H	-2.170148	2.705058	0.068839
H	0.000030	4.234344	0.116221
H	2.170171	2.704864	0.069052
H	-3.739887	0.319094	0.964997
H	-3.290982	0.859442	-0.668836
H	-4.338524	-0.553727	-0.462325
H	-1.618750	-2.848512	0.043717
H	-3.078647	-2.548877	1.018774
H	-3.204588	-2.667844	-0.751320
H	3.739703	0.319378	0.964951
H	4.338568	-0.553626	-0.462128
H	3.290940	0.859437	-0.669024
H	1.618929	-2.848566	0.043560
H	3.204849	-2.667767	-0.751225
H	3.078633	-2.548829	1.018842

S1

N[1],*N*[1],*N*[3],*N*[3]-Tetramethylpentalene-1,3-diamine

E = -576.494482825 Hartree

Number of imaginary frequencies = 0

C	-1.147363	-0.426875	-0.051992
C	-0.710020	0.952648	-0.056822
C	0.710046	0.952646	-0.056798
C	1.147382	-0.426888	-0.051941
C	-1.149849	2.291941	0.021385
C	0.000025	3.110962	0.059515
C	1.149897	2.291912	0.021419
N	-2.424003	-0.858753	-0.066996
N	2.424009	-0.858785	-0.066839
C	-3.521323	0.097266	0.007615
C	-2.720619	-2.278888	0.068456
C	3.521349	0.097229	0.007544
C	2.720495	-2.278970	0.068437
H	-2.166455	2.657290	0.058679
H	0.000035	4.190701	0.113415
H	2.166512	2.657237	0.058744
H	-3.614712	0.532867	1.009684
H	-3.357670	0.905408	-0.706577
H	-4.454172	-0.406202	-0.245050
H	-2.095640	-2.860941	-0.611622
H	-2.548740	-2.634642	1.091760
H	-3.763854	-2.455217	-0.191819
H	3.614833	0.532956	1.009548
H	4.454172	-0.406270	-0.245147
H	3.357635	0.905289	-0.706732
H	2.096453	-2.860811	-0.612711
H	3.764090	-2.455157	-0.190453
H	2.547220	-2.635108	1.091366
C	0.000016	-1.266736	-0.033314
H	0.000032	-2.342115	-0.007267

3H$^+$-a

1,3-Bis(dimethylamino)cyclopenta[*c*]pyrrol-2-ium

E = -592.602119620 Hartree

Number of imaginary frequencies = 0

C	1.137824	-0.356691	0.013669
C	0.715182	0.986796	0.039387
C	-0.715224	0.986788	0.039451
C	-1.137826	-0.356719	0.013731
N	-0.000001	-1.173975	-0.044705
C	1.151461	2.320193	-0.058855
C	-0.000035	3.123282	-0.106716
C	-1.151524	2.320179	-0.058740
N	2.345653	-0.865723	0.023592
N	-2.345642	-0.865781	0.023681
C	3.478347	0.056676	0.145563
C	2.588916	-2.303680	-0.101475
C	-3.478344	0.056625	0.145528
C	-2.588828	-2.303741	-0.101561
H	2.172285	2.685816	-0.092977
H	-0.000048	4.206031	-0.170615
H	-2.172352	2.685807	-0.092685
H	3.626899	0.598618	-0.797203
H	3.281570	0.771646	0.952035
H	4.376668	-0.515562	0.385085
H	1.875218	-2.746336	-0.804255
H	3.593112	-2.454250	-0.503983
H	2.522798	-2.801120	0.875188
H	-3.626623	0.598744	-0.797182
H	-4.376746	-0.515620	0.384719
H	-3.281758	0.771449	0.952176
H	-1.875213	-2.746232	-0.804532
H	-2.522491	-2.801329	0.875011
H	-3.593093	-2.454320	-0.503887
H	0.000012	-2.123232	0.305229

3H$^+$-b

3-(Dimethylamino)-*N*,*N*-dimethylcyclopenta[*c*]pyrrol-1-aminium

E = -592.559245831 Hartree

Number of imaginary frequencies = 0

C	1.152947	-0.407081	0.060595
C	0.679760	0.979887	-0.045186
C	-0.754813	0.900363	0.029847
C	-0.975467	-0.445426	0.161468
N	0.095550	-1.257354	0.180828
C	1.041850	2.295663	-0.155613
C	-0.185453	3.081195	-0.154367
C	-1.267323	2.254882	-0.039496

N	2.401064	-0.798207	0.052472
N	-2.266732	-1.140509	0.261179
C	3.464646	0.194990	-0.079828
C	2.773532	-2.206294	0.182302
C	-3.388308	-0.232393	0.642567
C	-2.548223	-1.874504	-1.022866
H	2.040015	2.716536	-0.229685
H	-0.221137	4.162800	-0.230002
H	-2.303179	2.576250	-0.005592
H	3.345610	0.750729	-1.018213
H	3.433167	0.894674	0.765026
H	4.427258	-0.320393	-0.085683
H	1.868439	-2.804837	0.287993
H	3.328699	-2.516697	-0.710205
H	3.410492	-2.329529	1.065579
H	-3.538746	0.473927	-0.178283
H	-4.288828	-0.833298	0.792558
H	-3.115936	0.301549	1.556151
H	-1.702101	-2.535315	-1.222271
H	-3.477928	-2.437452	-0.904216
H	-2.645357	-1.126156	-1.814295
H	-2.128544	-1.847983	0.993242

14

4,5,6-Tribromo-N^1,N^1,N^3,N^3-tetramethylcyclopenta[*c*]pyrrole-1,3-diamine

E = -8313.18497577 Hartree

Number of imaginary frequencies = 0

C	-2.218161	1.089460	-0.009248
C	-0.827620	0.716816	-0.194213
C	-0.827477	-0.717050	-0.194235
C	-2.217976	-1.089930	-0.009245
N	-3.014052	-0.000296	0.132570
C	0.509425	1.146139	-0.090216
C	1.330505	0.000131	-0.053221
C	0.509666	-1.146064	-0.090178
N	-2.779578	2.305066	0.006332
N	-2.779191	-2.305617	0.006406
C	-2.132453	3.496955	-0.526502
C	-4.189120	2.466596	0.356960
C	-2.131760	-3.497521	-0.525990
C	-4.188695	-2.467418	0.357088
H	-1.888784	4.205942	0.269475
H	-1.223104	3.231750	-1.054580
H	-2.817646	3.981134	-1.229395
H	-4.524665	1.609054	0.931533
H	-4.299854	3.379874	0.946941
H	-4.805233	2.553013	-0.544954
H	-1.888114	-4.206241	0.270239
H	-2.816734	-3.982033	-1.228866
H	-1.222359	-3.232319	-1.053969
H	-4.524478	-1.609773	0.931363
H	-4.804757	-2.554313	-0.544813
H	-4.299188	-3.380535	0.947364
Br	1.210683	-2.901274	0.076923
Br	3.220168	0.000364	0.023563
Br	1.209911	2.901529	0.077128

17

5-Bromo-N^1,N^1,N^3,N^3-tetramethylcyclopenta[*c*]pyrrole-1,3-diamine

E = -3166.11350735 Hartree

Number of imaginary frequencies = 0

C	1.657004	-1.084593	-0.035313
C	0.263193	-0.709399	-0.071913
C	0.263199	0.709411	-0.071922
C	1.656979	1.084604	-0.035316
N	2.480033	-0.000006	-0.004383
C	-1.070821	-1.163034	-0.029379
C	-1.864261	0.000015	-0.012641
C	-1.070837	1.163047	-0.029394
N	2.158434	-2.325322	-0.036444
N	2.158437	2.325319	-0.036444
C	1.276915	-3.484841	-0.062132
C	3.588883	-2.573697	0.109449
C	1.276927	3.484846	-0.062144
C	3.588884	2.573680	0.109477
H	-1.449582	-2.172838	0.000174
H	-1.449587	2.172856	0.000128
H	0.911423	-3.734850	0.940345
H	0.424360	-3.293361	-0.712916
H	1.829444	-4.338967	-0.455608
H	4.112015	-1.622066	0.110396
H	3.787370	-3.101116	1.048763
H	3.945850	-3.190581	-0.720545
H	0.911410	3.734845	0.940327

H	1.829472	4.338973	-0.455596
H	0.424387	3.293377	-0.712950
H	4.112009	1.622046	0.110417
H	3.945867	3.190577	-0.720501
H	3.787359	3.101083	1.048803
Br	-3.774186	-0.000005	0.036201

23

N[1],*N*[1],*N*[3],*N*[3]-Tetramethyl-4,5,6-triphenylcyclopenta[*c*]pyrrole-1,3-diamine

E = -1285.89219402 Hartree

Number of imaginary frequencies = 0

C	1.078655	-2.706945	0.153158
C	0.711667	-1.308955	0.035760
C	-0.711404	-1.309053	-0.036206
C	-1.078197	-2.707193	-0.153224
N	0.000248	-3.520556	0.000021
C	1.164272	0.033154	0.016032
C	-0.000035	0.857487	0.000032
C	-1.164226	0.032924	-0.016123
N	2.272243	-3.263639	0.414085
N	-2.271824	-3.263966	-0.413881
C	3.429624	-2.507257	0.868389
C	2.434824	-4.714991	0.419470
C	-3.429175	-2.507622	-0.868399
C	-2.434314	-4.715315	-0.419003
H	4.188470	-2.423432	0.084588
H	3.136271	-1.510175	1.179146
H	3.870741	-3.025447	1.725257
H	1.598687	-5.175717	-0.097021
H	3.372312	-4.968874	-0.083445
H	2.476080	-5.095626	1.446507
H	-3.870605	-3.026321	-1.724789
H	-4.187796	-2.423030	-0.084481
H	-3.135624	-1.510837	-1.179964
H	-1.597702	-5.175889	0.096856
H	-3.371373	-4.969272	0.084681
H	-2.476377	-5.096026	-1.445983
C	2.551073	0.543529	-0.054488
C	3.052113	1.437702	0.904152
C	3.396394	0.166303	-1.110493
C	4.353986	1.924924	0.817827
H	2.412392	1.747884	1.721914
C	4.697057	0.657387	-1.200651
H	3.017122	-0.506516	-1.872199
C	5.183245	1.537350	-0.234424
H	4.720708	2.611710	1.572987
H	5.328919	0.358568	-2.030424
H	6.194864	1.921499	-0.303679
C	-0.000190	2.340341	0.000106
C	-0.669860	3.065697	0.998384
C	0.669328	3.065963	-0.998090
C	-0.672601	4.458594	0.998159
H	-1.189470	2.528385	1.783320
C	0.671774	4.458853	-0.997691
H	1.189043	2.528851	-1.783095
C	-0.000488	5.163247	0.000280
H	-1.197408	4.994362	1.782053
H	1.196460	4.994839	-1.781517
H	-0.000597	6.247688	0.000340
C	-2.551111	0.543118	0.054340
C	-3.052221	1.437111	-0.904427
C	-3.396408	0.165919	1.110359
C	-4.354147	1.924211	-0.818193
H	-2.412509	1.747268	-1.722207
C	-4.697125	0.656885	1.200435
H	-3.017087	-0.506769	1.872158
C	-5.183383	1.536682	0.234090
H	-4.720917	2.610869	-1.573445
H	-5.328980	0.358103	2.030225
H	-6.195043	1.920733	0.303293

24

N[1],*N*[1],*N*[3],*N*[3]-Tetramethyl-5-phenylcyclopenta[*c*]pyrrole-1,3-diamine

E = -823.683058594 Hartree

Number of imaginary frequencies = 0

C	2.129203	-1.081259	-0.078378
C	0.736598	-0.707018	-0.102488
C	0.734694	0.712626	-0.046290
C	2.126161	1.087228	0.013732
N	2.950847	0.002644	0.003390
C	-0.596137	-1.152017	-0.084462
C	-1.429706	-0.001799	-0.031460
C	-0.599202	1.151653	-0.000030
N	2.636085	-2.320232	-0.130846
N	2.629507	2.327696	0.064521
C	1.758907	-3.479673	-0.209542

C	4.065419	-2.570335	0.018171
C	1.748848	3.486976	0.088250
C	4.058785	2.568485	0.228618
H	-0.954910	-2.170393	-0.121435
H	-0.959864	2.165850	0.092618
H	1.398000	-3.780423	0.780910
H	0.902855	-3.258998	-0.846273
H	2.312199	-4.312054	-0.646735
H	4.586199	-1.618074	0.055069
H	4.259087	-3.128377	0.940954
H	4.430738	-3.158400	-0.829055
H	1.389352	3.699890	1.101648
H	2.298680	4.355636	-0.276748
H	0.891713	3.319333	-0.563121
H	4.582272	1.618145	0.181936
H	4.418892	3.227994	-0.566654
H	4.254838	3.046109	1.194973
C	-2.898365	-0.003617	-0.002110
C	-3.641089	1.092380	-0.478400
C	-3.620258	-1.100281	0.503968
C	-5.032336	1.095127	-0.443837
H	-3.118680	1.943535	-0.900571
C	-5.011606	-1.103459	0.527157
H	-3.080979	-1.951768	0.903632
C	-5.729635	-0.004256	0.056362
H	-5.575389	1.955261	-0.821412
H	-5.538218	-1.963964	0.926544
H	-6.813537	-0.004632	0.078696

25

4,5-Dibromo-N^1,N^1,N^3,N^3-tetramethyl-6-((triisopropylsilyl)ethynyl)cyclopenta[*c*]pyrrole-1,3-diamine

E = -6460.49295031 Hartree

Number of imaginary frequencies = 0

C	3.706406	1.577754	-0.028753
C	2.784544	0.461211	-0.170568
C	1.488075	1.049274	-0.187454
C	1.720330	2.475022	-0.029246
N	3.045547	2.754528	0.085043
C	2.617214	-0.940063	-0.104825
C	1.239452	-1.202434	-0.113492
C	0.506688	0.019606	-0.149116
N	5.045457	1.586164	-0.020422
N	0.843759	3.484578	-0.002119
C	5.864919	0.478888	-0.494150
C	5.776122	2.817192	0.274342
C	-0.564528	3.342869	-0.339060
C	1.281459	4.853990	0.258681
H	6.403643	0.003125	0.330267
H	5.251782	-0.264404	-0.990972
H	6.594822	0.866876	-1.211375
H	5.137015	3.499712	0.825475
H	6.657692	2.565660	0.869252
H	6.103032	3.304565	-0.650914
H	-1.190065	3.562413	0.531497
H	-0.815332	4.052770	-1.133891
H	-0.784248	2.338368	-0.681159
H	2.297728	4.845622	0.638447
H	1.245042	5.445423	-0.662644
H	0.608493	5.308380	0.991152
Br	0.444499	-2.920155	-0.077844
C	-0.900882	0.076850	-0.110717
C	-2.119856	0.066997	-0.071086
Si	-3.958558	-0.030657	0.032233
C	-4.555991	1.054058	1.506795
H	-5.512542	0.603824	1.809568
C	-4.453225	-1.861855	0.317462
H	-5.553047	-1.858742	0.296408
C	-4.666115	0.496654	-1.672871
H	-4.311116	-0.301776	-2.337726
C	-4.835024	2.528804	1.159078
H	-5.591266	2.643117	0.379978
H	-5.193155	3.066915	2.044669
H	-3.927950	3.038718	0.818674
C	-3.603429	0.988054	2.718080
H	-3.407066	-0.032342	3.050627
H	-2.636075	1.438572	2.478064
H	-4.026722	1.538458	3.566590
C	-6.206578	0.477430	-1.724819
H	-6.560955	0.673539	-2.743360
H	-6.619904	-0.487806	-1.418637
H	-6.647132	1.243648	-1.079234
C	-4.105161	1.813276	-2.240906
H	-4.431559	2.682724	-1.664469
H	-3.012178	1.809560	-2.255554
H	-4.448206	1.961802	-3.271590
C	-3.965710	-2.782590	-0.818654
H	-4.299431	-3.812142	-0.645213
H	-4.346644	-2.479254	-1.797364
H	-2.873534	-2.795754	-0.875401

C	-4.023159	-2.432563	1.681791
H	-4.471906	-1.891390	2.518226
H	-4.333414	-3.480203	1.769998
H	-2.936239	-2.403575	1.802935
Br	3.915318	-2.313691	0.073439

26

5-Bromo-N^1,N^1,N^3,N^3-tetramethyl-4,6-bis((triisopropylsilyl)ethynyl)cyclopenta[*c*]pyrrol-1,3-diamine

E = -4607.80164946 Hartree

Number of imaginary frequencies = 0

C	1.040254	3.477079	-0.001874
C	0.679891	2.067561	-0.084065
C	-0.734888	2.043986	-0.079263
C	-1.140059	3.441356	0.006743
N	-0.062629	4.263775	0.061793
C	1.167155	0.726186	-0.086294
C	0.008685	-0.089849	-0.091259
C	-1.176854	0.686098	-0.079924
N	2.246251	4.055430	0.011221
N	-2.363664	3.981196	0.029950
C	3.497848	3.342695	-0.191161
C	2.383216	5.504179	0.145696
C	-3.594077	3.228169	-0.153888
C	-2.546134	5.425013	0.163781
H	4.136004	3.447155	0.692088
H	3.327089	2.288769	-0.375247
H	4.024452	3.772138	-1.049838
H	1.425822	5.936922	0.415359
H	3.126182	5.721898	0.918223
H	2.726361	5.938612	-0.799430
H	-4.227085	3.322590	0.734164
H	-4.141895	3.631738	-1.011893
H	-3.391992	2.178107	-0.327928
H	-1.597025	5.890817	0.406055
H	-2.929405	5.842935	-0.773271
H	-3.275064	5.621186	0.955351
Br	0.044709	-1.984587	-0.108331
C	-2.469552	0.124095	-0.054045
C	-3.563307	-0.414908	-0.029315
C	2.477133	0.206315	-0.068291
C	3.584624	-0.303758	-0.049031
Si	-5.183283	-1.293899	0.025104
Si	5.215092	-1.162949	-0.033646
C	-6.268130	-0.517793	1.414239
H	-6.945642	-1.328805	1.718566
C	-4.858500	-3.145656	0.404740
H	-5.851298	-3.617279	0.359240
C	-5.952271	-1.190127	-1.730928
H	-5.246089	-1.774090	-2.335912
C	-7.146053	0.668021	0.970740
H	-7.835883	0.404614	0.166521
H	-7.747560	1.031301	1.812393
H	-6.537887	1.509689	0.623808
C	-5.444797	-0.107576	2.652278
H	-4.838327	-0.922845	3.049654
H	-4.761368	0.711879	2.411905
H	-6.107919	0.235462	3.455236
C	-7.323802	-1.884976	-1.839320
H	-7.668789	-1.898210	-2.879662
H	-7.291004	-2.922869	-1.495899
H	-8.091233	-1.368866	-1.253958
C	-5.989707	0.219002	-2.350800
H	-6.699615	0.874611	-1.839923
H	-5.007052	0.696804	-2.319923
H	-6.298056	0.165883	-3.401589
C	-3.972269	-3.814999	-0.664118
H	-3.838186	-4.878946	-0.437068
H	-4.402792	-3.749434	-1.666768
H	-2.979332	-3.357607	-0.697128
C	-4.287410	-3.414826	1.809563
H	-4.962069	-3.083264	2.602577
H	-4.121683	-4.488859	1.953064
H	-3.324806	-2.914782	1.952131
C	6.358564	-0.374195	1.299015
H	7.029508	-1.195920	1.587273
C	5.957620	-1.039063	-1.802250
H	7.021847	-1.296856	-1.703367
C	4.891357	-3.003627	0.389277
H	4.254086	-3.356723	-0.431465
C	5.587184	0.056411	2.562408
H	4.896738	0.875483	2.340805
H	4.994808	-0.754282	2.991001
H	6.281437	0.405045	3.336156
C	7.253764	0.780460	0.813174
H	7.923943	1.106401	1.617351
H	7.878151	0.497444	-0.037950
H	6.661249	1.652231	0.517234
C	6.183618	-3.844009	0.375676
H	5.953940	-4.907240	0.509695

H	6.741116	-3.747042	-0.560566
H	6.856529	-3.557892	1.190863
C	4.110057	-3.233227	1.695690
H	3.182167	-2.657483	1.723593
H	3.847690	-4.292239	1.802505
H	4.704739	-2.962136	2.573659
C	5.317926	-2.045846	-2.779483
H	5.757296	-1.940455	-3.778190
H	5.459588	-3.083299	-2.468657
H	4.241514	-1.872426	-2.878375
C	5.867936	0.378680	-2.401269
H	6.355372	1.133070	-1.780723
H	6.343864	0.408058	-3.388392
H	4.823571	0.677393	-2.531579

27

N[1],*N*[1],*N*[3],*N*[3]-Tetramethyl-5-((triisopropylsilyl)ethynyl)cyclopenta[*c*]pyrrole-1,3-diamine

E = -1313.41822891 Hartree

Number of imaginary frequencies = 0

C	-4.248646	1.045787	-0.002248
C	-2.852641	0.684858	-0.075335
C	-2.842054	-0.737435	-0.137184
C	-4.232550	-1.123939	-0.097363
N	-5.060941	-0.047092	-0.005506
C	-1.528039	1.140238	-0.039919
C	-0.689454	-0.011053	-0.088588
C	-1.510761	-1.174513	-0.139838
N	-4.761526	2.280498	0.057769
N	-4.727137	-2.366534	-0.146922
C	-3.891039	3.448574	0.071299
C	-6.191545	2.508648	0.236978
C	-3.839611	-3.518339	-0.236118
C	-6.153811	-2.630558	0.008104
H	-1.160470	2.153388	0.027212
H	-1.128672	-2.184313	-0.159955
H	-3.518951	3.661473	1.079900
H	-3.042508	3.291579	-0.593655
H	-4.454992	4.312526	-0.282639
H	-6.707066	1.553554	0.201358
H	-6.379752	2.989676	1.202945
H	-6.566469	3.160542	-0.557532
H	-3.464020	-3.814285	0.749971
H	-4.390994	-4.355703	-0.665688
H	-2.993747	-3.290639	-0.883826
H	-6.683017	-1.683674	0.059047
H	-6.519030	-3.212301	-0.843382
H	-6.335475	-3.200626	0.925644
C	0.723627	0.000669	-0.074593
C	1.943855	0.013601	-0.062131
Si	3.775736	0.046080	-0.027738
C	4.418095	-1.680552	-0.572652
H	4.250810	-1.688207	-1.657342
C	4.383316	0.484914	1.741164
H	5.477548	0.383251	1.700345
C	4.400762	1.382394	-1.269591
H	5.377017	1.708701	-0.882498
C	4.072852	1.935492	2.156172
H	2.995029	2.127208	2.156613
H	4.439299	2.129719	3.171365
H	4.544198	2.666900	1.495262
C	3.481132	2.618872	-1.328919
H	3.320270	3.075187	-0.350202
H	3.910343	3.385018	-1.985793
H	2.498443	2.350207	-1.726536
C	3.861103	-0.489608	2.813908
H	4.228479	-0.202195	3.806443
H	2.767482	-0.485123	2.852325
H	4.181360	-1.518333	2.633945
C	5.932237	-1.845991	-0.337862
H	6.294912	-2.783815	-0.774859
H	6.516182	-1.033300	-0.781533
H	6.170669	-1.874875	0.730257
C	3.638996	-2.880043	-0.002727
H	3.975858	-3.812578	-0.471738
H	3.787039	-2.990564	1.075074
H	2.565366	-2.782932	-0.179304
C	4.623758	0.845093	-2.696526
H	4.971797	1.647653	-3.357627
H	5.368184	0.046079	-2.734150
H	3.694319	0.454919	-3.124685

S6 References

1. CrystalClear SM 1.4.0 Rigaku/MSC Inc., 2008.
2. NUMABS: T. Higashi, (1998), rev. 2002. (Rigaku/MSC Inc.)
3. Sheldrick, G. M. A Short History of SHELX. *Acta Cryst.* **2008**, *A64*, 112–122.
4. Sheldrick, G. M. Crystal Structure Refinement with SHELXL. *Acta Cryst.* **2015**, *C71*, 3–8.
5. Farrugia, L. J. WinGX and ORTEP for Windows: An Update. *J. Appl. Crystallogr.* **2012**, *45*, 849–854.
6. Spek, A. L. Structure Validation in Chemical Crystallography. *Acta Cryst.* **2009**, *D65*, 148–155.
7. Dolomanov, O. V.; Bourhis, L. J.; Gildea, R. J.; Howard, J. A. K.; Puschmann, H. OLEX2: A Complete Structure Solution, Refinement and Analysis Program. *J. Appl. Cryst.* **2009**, *42*, 339–341.
8. Macrae, C. F.; Edgington, P. R.; McCabe, P.; Pidcock, E.; Shields, G. P.; Taylor, R.; Towler, M.; van de Streek, J. Mercury: Visualization and Analysis of Crystal Structures. *J. Appl. Cryst.* **2006**, *39*, 453-457.
9. Frisch, M. J.; Trucks, G. W.; Schlegel, H. B.; Scuseria, G. E.; Robb, M. A.; Cheeseman, J. R.; Scalmani, G.; Barone, V.; Mennucci, B.; Petersson, G. A.; Nakatsuji, H.; Caricato, M.; Li, X.; Hratchian, H. P.; Izmaylov, A. F.; Bloino, J.; Zheng, G.; Sonnenberg, J. L.; Hada, M.; Ehara, K.; Toyota, K.; Fukuda, R.; Hasegawa, J.; Ishida, M.; Nakajima, T.; Honda, Y.; Kitao, O.; Nakai, H.; Vreven, T.; Montgomery, J. A.; Peralta, J. E. Jr.; Ogliaro, F.; Bearpark, M.; Heyd, J. J.; Brothers, E.; Kudin, K. N.; Staroverov, V. N.; Keith, T.; Kobayashi, R.; Normand, J.; Raghavachari, K.; Rendell, A.;Burant, J. C.; Iyengar, S. S.; Tomasi, J.; Cossi, M.; Rega, N.; Millam, J. M.; Klene, M.; Knox, J. E.; Cross, J. B.; Bakken, V.; Adamo, C.; Jaramillo, J.; Gomperts, R.; Stratmann, R. E.; Yazyev, O.; Austin, A. J.; Cammi, R.; Pomelli, C.; Ochterski, J. W.; Martin, R. L.; Morokuma, K.; Zakrzewski, V. G.; Voth, G. A.; Salvador, P.; Dannenberg, J. J.; Dapprich, S.; Daniels, A. D.; Farkas, O.; Foresman, J. B.; Ortiz, J. V.; Cioslowski, J.; Fox, D. J. Gaussian 09, Revision E.01, Gaussian, Inc., Wallingford CT, 2013.
10. Karas, L. J.; Jalife. S.; Viesser, R. V.; Soares, J. V.; Haley, M. M.; Wu, J. I. Tetra-*tert*-butyl-*s*-indacene is a Bond Localized C_{2h} Structure and a Challenge for Computational Chemistry *Angew. Chem. Int. Ed.* **2023**, e202307379.

11. Stephens, P. J.; Devlin, F. J.; Chabalowski, C. F.; Frisch, M. J. Ab Initio Calculation of Vibrational Absorption and Circular Dichroism Spectra Using Density Functional Force Fields. *J. Phys. Chem.* **1994**, *98*, 11623–11627.
12. Yanai, T.; Tew, D.; Handy, N. A new hybrid exchange-correlation functional using the Coulomb-attenuating method (CAM-B3LYP) *Chem. Phys. Lett.*, **2004**, *393*, 51–57.
13. Chai, J.-D.; Head-Gordon, M. Long-range corrected hybrid density functionals with damped atom-atom dispersion corrections *Phys. Chem. Chem. Phys.*, **2008**, *10*, 6615–6620.
14. Zhao, Y.; Truhlar, D. G. The M06 suite of density functionals for main group thermochemistry, thermochemical kinetics, noncovalent interactions, excited states, and transition elements: two new functionals and systematic testing of four M06-class functionals and 12 other functionals *Theor. Chem. Acc.*, **2008**, *120*, 215–241.
15. Peverati, R.; Truhlar, D. G. Improving the Accuracy of Hybrid Meta-GGA Density Functionals by Range Separation *J. Phys. Chem. Lett.* **2011**, 2, 2810–2817.
16. Peverati, R.; Truhlar, D. G. M11-L: A Local Density Functional That Provides Improved Accuracy for Electronic Structure Calculations in Chemistry and Physics *J. Phys. Chem. Lett.* **2012**, *3*, 117–124.
17. Krishnan, R.; Binkley, K. S.; Seeger, R.; Pople, J. Self-Consistent Molecular Orbital Methods. XX. A Basis Set for Correlated Wave Functions. *J. Chem. Phys.* **1980**, *72*, 650–654.
18. Gershoni-Poranne, R.; Stanger, A. Magnetic criteria of aromaticity *Chem. Soc. Rev.* **2015**, *44,* 6597–6615.
19. Krygowski, T. M.; Cyrański, M. K. Structural aspects of aromaticity *Chem. Rev.* **2001**, *101*, 1385–1420.
20. Feixas, F.; Matito, E.; Poater, J.; Sola, M. Quantifying aromaticity with electron delocalisation measures *Chem. Soc. Rev.* **2015**, *44*, 6434–6451.
21. Mucsi, Z.; Viskolcz, B.; Csizmadia, I. G. A Quantitative scale for the degree of aromaticity and antiaromaticity: a comparison of theoretical and experimental enthalpies of hydrogenation *J. Phys. Chem. A*, **2007**, *111*, 1123–1132.
22. Pino-Rios, R.; Inostroza, D.; Cárdenas-Jirón, G.; Tiznado, W. Orbital-weighted dual descriptor for the study of local reactivity of systems with (quasi-) degenerate states *J. Phys. Chem. A* **2019**, *123*, 10556–10562.
23. Zubarev, D. Y.; Boldyrev, A. I. Developing paradigms of chemical bonding: adaptive natural density partitioning Phys. *Chem. Chem. Phys.* **2008**, *10*, 5207–5217.

24. Herges, R.; Geuenich, D. Delocalization of Electrons in Molecules. J. *Phys. Chem. A* **2001**, *105*, 3214–3220.
25. Geuenich, D.; Hess, K.; Köhler F.; Herges, R. Anisotropy of the Induced Current Density (ACID), a General Method to Quantify and Visualize Electronic Delocalization. *Chem. Rev.* **2005**, *105*, 3758–3772.
26. Gershoni-Poranne, R.; Stanger, A. The NICS-XY-Scan: Identification of Local and Global Ring Currents in Multi-Ring Systems. *Chem. Eur. J.* **2014**, *20*, 5673–5688.
27. Stanger, A. Obtaining Relative Induced Ring Currents Quantitatively from NICS. *J. Org. Chem.* **2010**, *75*, 2281–2288.
28. Stanger, A.; Rahalkar, A. Aroma, https://chemistry.technion.ac.il/en/team/amnon-stanger/
29. Krygowski, T. M. Crystallographic Studies of Inter- and Intramolecular Interactions Reflected in Aromatic Character of Pi-Electron Systems. *J. Chem. Inf. Model.* **1993**, *33*, 70– 78.
30. A data set collection of computational results is available in the ioChem-BD repository and can be accessed via https://doi.org/10.19061/iochem-bd-6-284; Álvarez-Moreno, M.; de Graaf, C.; Lopez, N.; Maseras, F.; Poblet, J.M.; Bo, C. *J. Chem. Inf. Model.* **2015**, *55*, 95−103.
31. Zhang, G.; Musgrave, C. B. Comparison of DFT Methods for Molecular Orbital Eigenvalue Calculations *J. Phys. Chem. A* **2007**, *111*, 1554–1561.
33. Thery, V.; Barra, C.; Simeoni, A.; Pecaut, J.; Tomas-Mendivil E.; Martin, D. Bending Enamine Patterns of Stabilized Pentalenes into "Polymethine Ylides". *Org. Lett.* **2023**, *25*, 560−564.
33. Marenich, A. V.; Cramer, C. J.; Truhlar D.G. Universal Solvation Model Based on Solute Electron Density and on a Continuum Model of the Solvent Defined by the Bulk Dielectric Constant and Atomic Surface Tensions *J. Phys. Chem. B* **2009**, *113*, 6378–6396.
